# Complexity and entropy representation for machine component diagnostics

**DOI:** 10.1371/journal.pone.0217919

**Published:** 2019-07-09

**Authors:** Srinivasan Radhakrishnan, Yung-Tsun Tina Lee, Sudarsan Rachuri, Sagar Kamarthi

**Affiliations:** 1 Mechanical and Industrial Engineering, Northeastern University, Boston, Massachusetts, United States of America; 2 Systems Integration Division, NIST, Gaithersburg, Maryland, United States of America; 3 Advanced Manufacturing Office, EERE, Department of Energy, Washington DC, United States of America; Universidade Federal de Alagoas, BRAZIL

## Abstract

The Complexity-entropy causality plane (CECP) is a parsimonious representation space for time series. It has only two dimensions: normalized permutation entropy ($H_{S}$) and Jensen-Shannon complexity ($C_{JS}$) of a time series. This two-dimensional representation allows for detection of slow or rapid drifts in the condition of mechanical components monitored through sensor measurements. The CECP representation can be used for both predictive analytics and visual monitoring of changes in component condition. This method requires minimal pre-processing of raw signals. Furthermore, it is insensitive to noise, stationarity, and trends. These desirable properties make CECP a good candidate for machine condition monitoring and fault diagnostics. In this work we study the effectiveness of CECP on three rotary component condition assessment applications. We use CECP representation of vibration signals to differentiate various machine component health conditions for rotary machine components, namely roller bearing and gears. The results confirm that the CECP representation is able to detect, with high accuracy, changes in underlying dynamics of machine component degradation states. From class separability perspective, the CECP representation is able to generate linearly separable classes for the classification of different fault states. This classification performance improves with increasing signal length. For signal length of 16,384 data points, the fault classification accuracy varies from 90% to 100% for bearing applications, and from 85% to 100% for gear applications. We observed that the optimum parameter for CECP representatino depends on the application. For bearing applications we found that embedding dimension *D* = 4, 5, 6, and embedding delay *τ* = 1, 2, 3 are suitable for good fault classification. For gear applications we find that embedding dimension *D* = 4, 5, and embedding delay *τ* = 1, 5 are suitable for fault classification.

## Introduction

Prognostics and health management (PHM) and its functions, diagnostics and prognostics, are key areas of interest for smart manufacturing. While, diagnostics detects and identifies a failure mode within a component, prognostics predicts the degradation trends of a component and estimates the remaining useful life (RUL) of the component. Table 1 lists the common machine component problems addressed by PHM [1]. This paper investigates a novel sensor data representation method that allows for automatic as well as visual detection of health condition of bearings and gears, which are the most prevalent components among the ones listed in Table 1.

**Table 1:** Common machine component and machine subsystem problems in PHM [1].

Machine Component/Subsystem	Problems
Bearing	Outer-race, inner- race, roller, cage failures
Gear	Manufacturing error, missing tooth, tooth pitting, gear crack, gear fatigue/wear
Shaft	Unbalance, bend, crack, misalignment, rub
Pump (machine subsystem)	Valve impact,score, fracture,pistons lap, defective bearing and revolving crank, hydraulic problem
Alternator (machine subsystem)	Stator faults, rotor electrical faults, rotor mechanical faults

*Bearings* perform a key function of converting sliding friction into rolling friction in rotary machines. A bearing consists of an inner ring, an outer ring, a set of ball rollers or cylindrical rollers (usually termed as rolling elements), and a cage. The rolling elements are placed inside a cage which is then set between the inner ring and outer ring. A bearing fault can occur in any of the aforementioned four components of a roller bearing. The causes for faults in bearings include, increase of operating loads, shaft imbalance or bent shaft, surface defects, surface roughness, surface contamination, and presence of particles on inner or outer raceway [2]. El-Thalji and Jantunen [3] presented dynamics of wear progression of roller bearings.

*Gears*, like bearings, serve a critical function in a rotary machine. Like bearings, gears operate in tough operating conditions involving static and impact forces. Gears are subjected to wear in the form of cracking, pitting, and scaling which eventually culminates into a chipped tooth or broken tooth condition. When such a fault state is reached, gears do not perform as expected and hence they are, like bearings, ought to be continuously monitored for their health condition.

For both bearings and gears, sensor-based monitoring methods are viable options for fault detection and estimation of RUL. The common indirect measurements include vibration, acoustic emission, and power sensors. The general steps for implementing machine learning models for machine component fault detection are signal acquisition, signal processing, feature extractions, and building a machine learning model for classification or regression.

Most of the sensor data generated in manufacturing are structured time series data. Dimension reduction is an inherent challenge, particularly when real-time data processing is required and data streams are generated continuously at high volume and high rate [4]. Dimension reduction through parsimonious signal representation and feature extraction are necessary for diagnostics and prognostics of mechanical components. Time domain features commonly include root mean square (RMS), average, kurtosis, crest factor, autoregressive model coefficients [2]. Studies have found that RMS extracted from vibration measurements exhibits a strong correlation with bearing wear [2, 5–8]. Similarly, kurtosis and crest factor are sensitive to the signal shape; kurtosis is also sensitive to rotational speed and the frequency bandwidth of a signal [2]. Fast-Fourier transform (FFT) is the most widely applied method for frequency domain analysis. However due to the non-stationary nature of the signals in PHM applications, time-frequency domain analysis is preferred. Wigner–Ville distribution (WVD), wavelet transform (WT) [9], discrete wavelet transform (DWT) [10], and short time Fourier transform (STFT) are some of the methods of choice for bearing fault diagnosis. Several studies have used time domain analysis for gear fault detection [11–14]. RMS and kurtosis are found to be sensitive to gear faults, especially cracked gears [14]. Both time domain and frequency domain features have been explored to represent signals for gear fault detection [15–18] and time-frequency domain [19–21] has been used for improving accuracy, enhancing robustness, and reducing sensitivity to noise.

This paper presents a different approach to signal processing for diagnostics and prognostics of bearings and gears. It investigates a complexity-entropy causality plane (CECP) based sensor data representation that is parsimonious and effective for detection of faults in bearings and gears. In Section 1 we reviewed different time, frequency and time-frequency methods for sensor data representation in PHM applications. In section 2 we review different entropic methods used for characterizing time series signals. In Section 3 we present the formulation for the CECP representation and discuss the parameter selection for machine component monitoring applications. In Section 4 we present the results for roller bearing fault detection and helical gear fault detection and discuss the merits of the CECP representation in these applications. In the conclusion section we discuss the advantages of CECP representation over existing time, frequency, and time-frequency based approaches for PHM applications.

## Materials and methods

### Sensor data representation methods

The representation of the sensor data is the process of transforming a raw time series vector *X* = {*x*_1_, *x*_2_, …, *x*_*n*_} into a vector *F* = {*f*_1_, *f*_2_, …, *f*_*k*_} in transformation space such that *k* < *n*, where *n* is the number of data points in a given time series, and *k* is the number of entities in the transformed space. By reducing the dimension, the computational complexity is reduced from O(n) to O(k). The desired properties of a good representation are preservation of local and global characteristics with low information loss and high robustness in the presence of noise and outliers.

The sensor signal analysis using time, frequency, or time-frequency domain are well suited when the underlying system dynamics exhibit a linear behavior. In case of machine components like bearing, gears, or coupled mechanical systems, sensor signals exhibit high complexity and nonlinear characteristics. In recent years, entropic measures have been proven to be effective for feature extraction from complex and nonlinear sensor signals generated by rotary components. Approximate Entropy (ApEn) [22–24] and Sample Entropy (SampEn) [25] were originally developed for characterizing nonlinear time series in biomedical applications. ApEn performance depends on the signal length: shorter the signal length lower the estimation accuracy [25]. SampEn is an improved version of ApEn. For PHM applications, ApEn [26, 27] method has been adopted for monitoring machine health and SampEn [28–30] for bearing fault diagnostics. The multi-scale entropy (MSE) method developed by Costa et al. [31, 32] proved effective in case of machine health monitoring where interaction between multiple components (bearing, gear, and shafts) generate vibration signals that contain multiple intrinsic oscillatory modes in which case single scale entropic methods such as ApEn and SampEn may be less effective for characterization of the measured signals [33, 34]. Unlike ApEn and SampEn, MSE analyzes signals in multiple time scales rather than in a single time scale, since entropy values do not necessarily capture complexity changes. Zhang et al. [33] applied MSE method for bearing fault application.

Permutation entropy, introduced by Bandt and Pompe [35] has been used for analyzing and characterizing nonlinear time series. Studies have adopted permutation entropy for fault detection of mechanical components [36, 37]. However, using permutation entropy alone as a feature limits its ability to distinguish different types of faults. Wu et al. [38] found that permutation entropy on its own as a feature does not fare well with classification algorithms. They used a technique called as multi-scale permutation entropy for feature extraction and a support vector machine (SVM) for fault signals classification. However, using multi-scale permutation entropy, one may end up with as many features as scales. This redundant information may hamper accuracy and further increase analysis time. Li et al. [39] used Laplacian score [40] to select the important features generated by multi-scale permutation entropy technique.

In this work we investigate CECP as a data representation and feature extraction technique for machine fault diagnostic and prognostics applications. The key interest in CECP representation stems from the fact that it can handle both stationary and non-stationary signals, reduce the number of features required for accurate classification or prediction, and allow for visualization of a time series as a point in a two-dimensional space. We investigated the effectiveness of CECP representation to detect machine component faults on publicly available sensor datasets on bearings and gears.

### CECP representation of sensor data

Shannon entropy, *S*[*P*], is a popular measure to compute the information associated with a process described by a probability distribution {*P* = *p*_*i*_ : *i* = 1, 2, …*M*}. However, for differentiating a simple process from a complex process, which exhibit different organizational properties, *S*[*P*], is ineffective [41, 42]. In addition, Shannon entropy on its own does not capture temporal relationship between measurements in a time series. It requires prior knowledge about the process in the form of an underlying probability distribution function [43] and it nevertheless poorly characterizes highly non-linear processes (e.g. chaotic systems).

To overcome the aforementioned limitations, Bandt and Pompe (BP) [35] provided a method to extract the underlying probability distribution from a time series. The BP method is effective since different permutation patterns that emerge from the time series reflect the dynamics of the underlying process. The BP method is non-parametric, rank based, and the probability of the ordinal patterns is invariant to nonlinear monotonic changes [42, 44]. This renders a good quantifier which is robust against nonlinear drifts and scaling [42] with ability to handle non-stationary time series [44–46]. The BP method for generating probability distributions is a simple symbolization technique that incorporates causality in the evaluation of the probability distributions associated with a time series [43]. For a given time series *X* = {*x*_*t*_ : *t* = 1, 2, …*N*}, at each time instance *s*, a sequence of measurements *X*_*s*_ = {*x*_*s*_, *x*_*s*+*τ*(1)_, …, *x*_*s*+*τ*(*D* − 1)_} is selected, where *D* represents the *embedding dimension* and *τ* represents the *embedding delay*. The embedding dimension *D* reflects the amount of information captures by *X*_*s*_. An ordinal pattern (0 1 … *D* − 1) is assigned to *X*_*s*_ such that (0 1 … *D* − 1) ↦ {*x*_*s*_, *x*_*s*+*τ*(1)_, …, *x*_*s*+*τ*(*D*−1)_}. The ordinal pattern is then shuffled according to the ascending order of measurements in *X*_*s*_. The shuffled ordinal pattern is referred to as permutation pattern and is represented as *π*. In case the elements of *X*_*s*_ are identical, the ordinal pattern is taken without shuffling as the resulting permutation pattern. When the embedded dimension is *D*, *D*! permutation patterns are possible. The relative frequency *p*_*i*_ of each *π*_*i*_ is obtained by dividing the count of permutation pattern *π*_*i*_ in the signal by the total number of permutation patterns (of any type) in the signal. Thus, $p_{i}=|\pi_{i}|/\sum_{i=1}^{D!}\left|\pi_{i}\right|$ for *i* = 1, 2, …, *D*! and *P* = {*p*_*i*_ : *i* = 1, 2, …, *D*!} is the probability distribution of the permutation patterns *π*_*i*_ in the signal. Here |*π*_*i*_| is the count of occurrence of permutation pattern *π*_*i*_. The *permutation entropy* is computed as

$$S\left[P\right]=-\sum_{i=1}^{D!}p_{i}log\left(p_{i}\right)$$ (1)

The permutation entropy as defined above takes maximum value when $p_{i}=p=\frac{1}{D!}$ for all *i*. From this the max *S*[*P*] = log*D*!. Thus the normalized permutation entropy is given by

$$H_{S}=\frac{S[P]}{\text{log}D!}$$ (2)

where $H_{S}\in \left(0,1\right)$.

Entropic measures, including *permutation entropy*, are able to quantify information but they do not capture the structure or patterns in a process [43, 47]. To uncover organizational properties of a process, several statistical complexity measures (SCM) have been developed [42]. Of them, Jensen-Shannon complexity, $C_{JS}$, which combines both information and disequilibrium measures, has potential to effectively detected the underlying dynamics. It is defined as [48, 49]

$$C_{JS}=Q_{J}\left[P,P_{e}\right]H_{S}$$ (3)

where *P*_*e*_ = {1/*D*!, 1/*D*!, …, 1/*D*!} is the uniform distribution and disequilibrium *Q*_*J*_[*P*, *P*_*e*_] is Jensen-Shannon divergence that links two probability distributions, *P* and *P*_*e*_:

$$Q_{J}\left[P,P_{e}\right]=Q_{0}J\left[P,P_{e}\right]$$ (4)

where

$$J\left[P,P_{e}\right]=S\left[\left(P+P_{e}\right)/2\right]-S\left[P\right]/2-S\left[P_{e}\right]/2$$ (5)

and *Q*_0_ is a normalization constant equal to the inverse of the maximum possible value of *J*[*P*, *P*_*e*_], which happens when one of the *p*_*i*_ of distribution *P* is 1 and all other *p*_*i*_ are 0 [42]. The *Q*_0_ is computed using the following equation [50]:

$$Q_{o}=\frac{1}{-\left(\frac{1}{2}\right)[\frac{D!+1}{D!}\text{log}\left(D!+1\right)-2\text{log}\left(2D!\right)+\text{log}\left(D!\right)]}.$$ (6)

The inclusion of factor *Q*_0_ ensures that 0 ≤ *Q*_*J*_[*P*, *P*_*e*_] ≤ 1. The Jensen-Shannon divergence quantifies the difference between two probability distributions, *P* and *P*_*e*_, using a non-trivial function of entropy. Here *P* is a probability distribution that represents the state of the system and *P*_*e*_ is a uniform distribution that serves as a reference [42]. As implied by the second law of thermodynamics, $H_{S}$ serves as the time dimension and the $C_{JS}$ versus $H_{S}$ graph maps the temporal evolution of the SCM [51]. For a given value of $H_{S}$, the values of complexity $C_{JS}$ varies between a minimum and maximum boundary which are termed the limit curves [52].

Signal length *N*, embedding dimension *D*, and embedding delay *τ* affect the value of permutation entropy. A large value of *N* may result in a near constant features on the CECP map, thereby neutralizing the ability of CECP to discern the dynamic changes. On the other hand, a small value of *N* may yield statistically insignificant results. Literature points out that in order to use CECP to differentiate chaotic processes from stochastic processes, it is necessary to satisfy the condition that signal length be relatively much larger than *D*!, i.e., *N* ≫ *D*! [44, 53]. The CECP representation is able to distinguish stochastic processes with different long range correlations when *D* is between 3 and 6 [44–46]. For practical purposes Bandt and Pompe [35] recommended 3 ≤ *D* ≤ 7 and *τ* = 1.

With respect to vibration signals for component fault diagnostics, Yan et al. [36] studied the relationship between *N* and $H_{S}$. After analyzing signals of different lengths (*N* = 32, 64, 128, 256, 512, 1024, and 2048), they reported that variation in $H_{S}$ values for *N* > 256 was insignificant; they observed a stable and near-constant $H_{S}$ value when *N* = 128 or *N* = 256. They further observed that when *D* < 4, permutation entropy was not able to detect the exact dynamic changes in the mechanical vibration signals; a *D* > 8 was computationally expensive; and time delay *τ* > 5 is not conducive for detecting small changes in the signal. In the end, they used *D* = 6 and *τ* = 3 for computing permutation entropy values for component fault diagnostics.

The CECP analysis has been extensively investigated for characterizing correlated stochastic processes [45, 54, 55], and distinguishing chaotic processes from stochastic processes [44, 51]. In econophysics, the CECP was used for quantification of stock market inefficiencies [56], evaluation of efficiency of bond markets [57], and analysis of commodities [58]. In addition, CECP was applied for river flow characterization [42], mountain stream temperature variation analysis [59], and song classification [50].

In this paper, we study the performance of CECP representation for identifying and monitoring faults of rotary machine components in three different applications: ball bearing dataset from the Machinery Failure Prevention Technology (MFPT) Society, bearing dataset from Case Western Reserve University, and gear dataset from the PHM Society.

## Results

### MFPT ball bearing experiment

**Fig 1:**
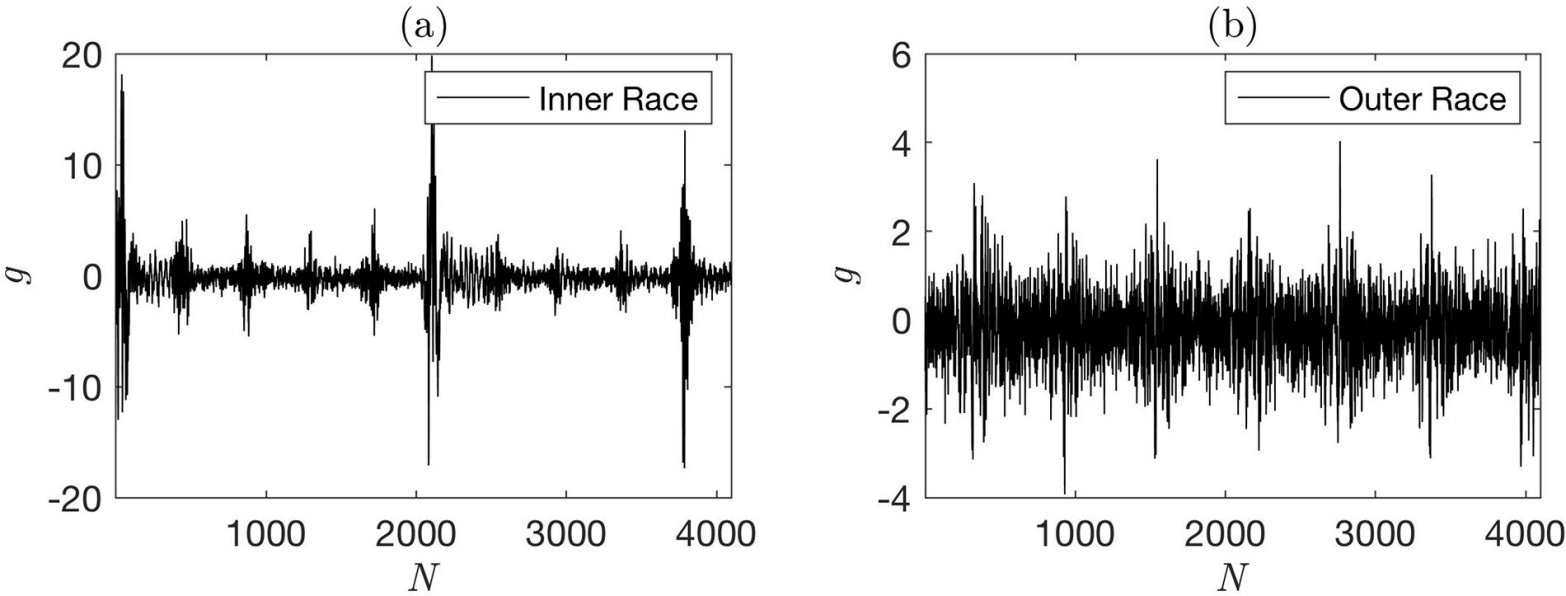
Sample vibration signals representing (a) an inner race fault and (b) an outer race fault.

In this application we consider two classes of vibration signals from ball bearing dataset provided by the MFPT Society [60]. Bechhoefer [60] complied this dataset from a set of bearing experiments. The dataset contains labeled signatures of faulty inner race and outer race of ball bearings. The signatures of faulty inner and outer races were generated at a constant shaft rotational speed of 25 rps (1500 rpm) and at seven different load conditions: 11.33, 22.67, 45.35, 68.0388, 90.71, 113.39, and 136.07 kg (or 25, 50, 100, 150, 200, 250, and 300 lbs). Each vibration signal is recorded for 3 seconds at 48,828 Hz frequency; it resulted in a signal of length 146,484 data points. Fig 1 shows a sample of vibration signals collected from the faulty inner race and outer race of the ball bearing. These sample signals were collected at 45.35 kg (100 lb) load and 25 rps (1500 rpm) shaft rotational speed. The vibration signals for both inner and outer race faults are periodic. The fault characteristic frequencies for inner race fault and outer race fault are outlined by Zhang et al. [61]. We performed Augmented Dickey-Fuller (ADF) test to verify the stationarity of the signals. For both the inner and outer race faults, we obtain a *p* value of 0.01 < 0.05 confirming the stationarity of the signals.

**Fig 2:**
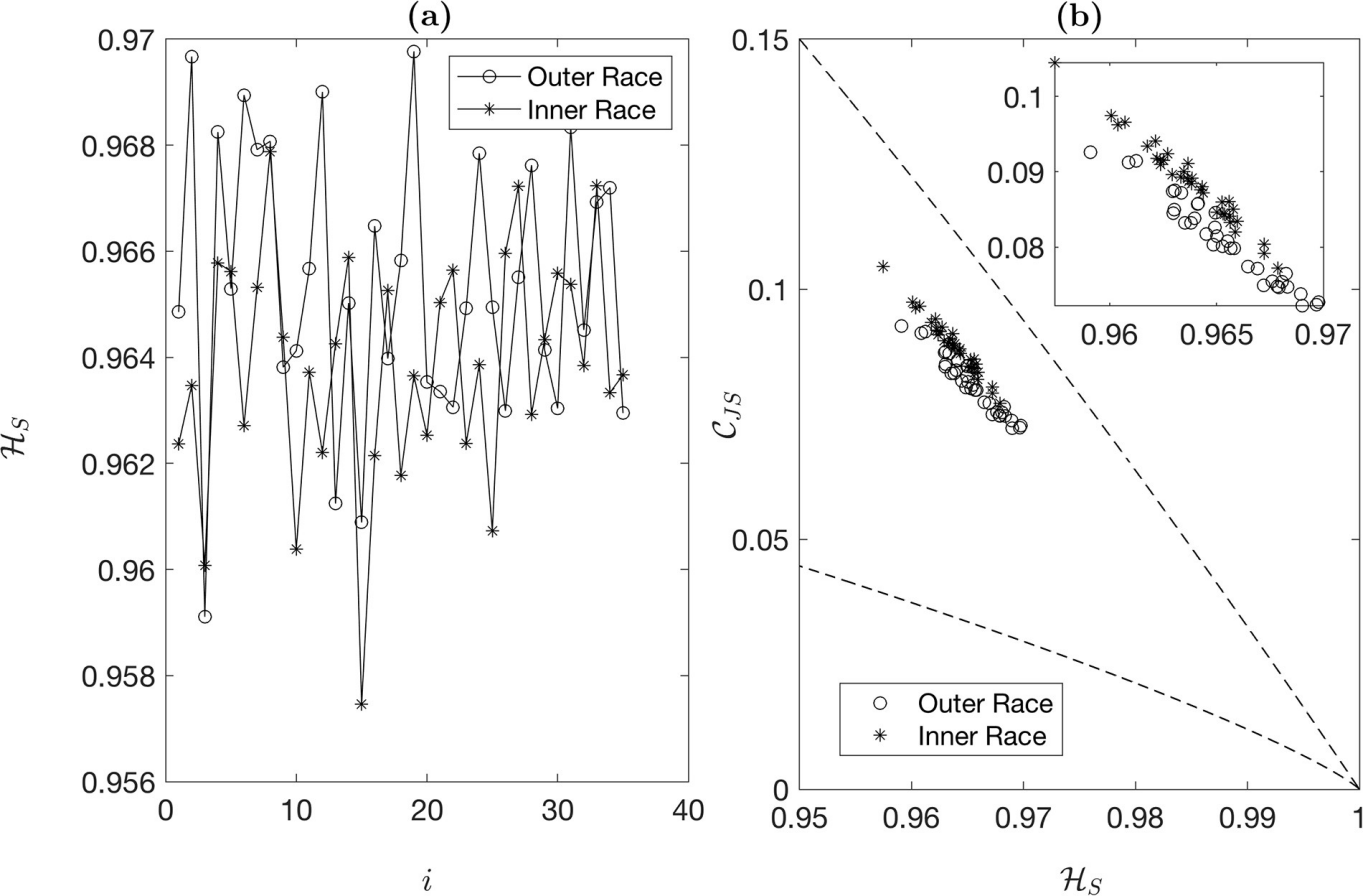
(a) Permutation entropy values of 35 inner race fault signals and 35 outer race fault signals, and (b) CECP values of 35 inner race fault signals and 35 outer race fault signals. The dashed lines represent the lower and upper limit curves. *N* = 4096, *D* = 6, and *τ* = 1. Inset figure is a scaled version of subplot (b).

The raw data includes inner and outer race fault signals of 146,484 data points each. For CECP computation, we segment each signal of 146,484 data points into 35 sub-signals of 4,096 data points each. Fig 2(a) plots $H_{S}$ values of 35 outer race and 35 inner race sub-signals. The overlap of $H_{S}$ for inner race and outer race signals indicates that $H_{S}$ is not an effective parameter to distinguish inner race faults from outer race faults. Fig 2(b) presents a CECP map (i.e., scatter plot of $C_{JS}$ vs. $H_{S}$) of 35 outer race and 35 inner race sub-signals. It is clear from the figure that the CECP representation is able to separate inner race faults from outer race faults in the plot.

**Fig 3:**
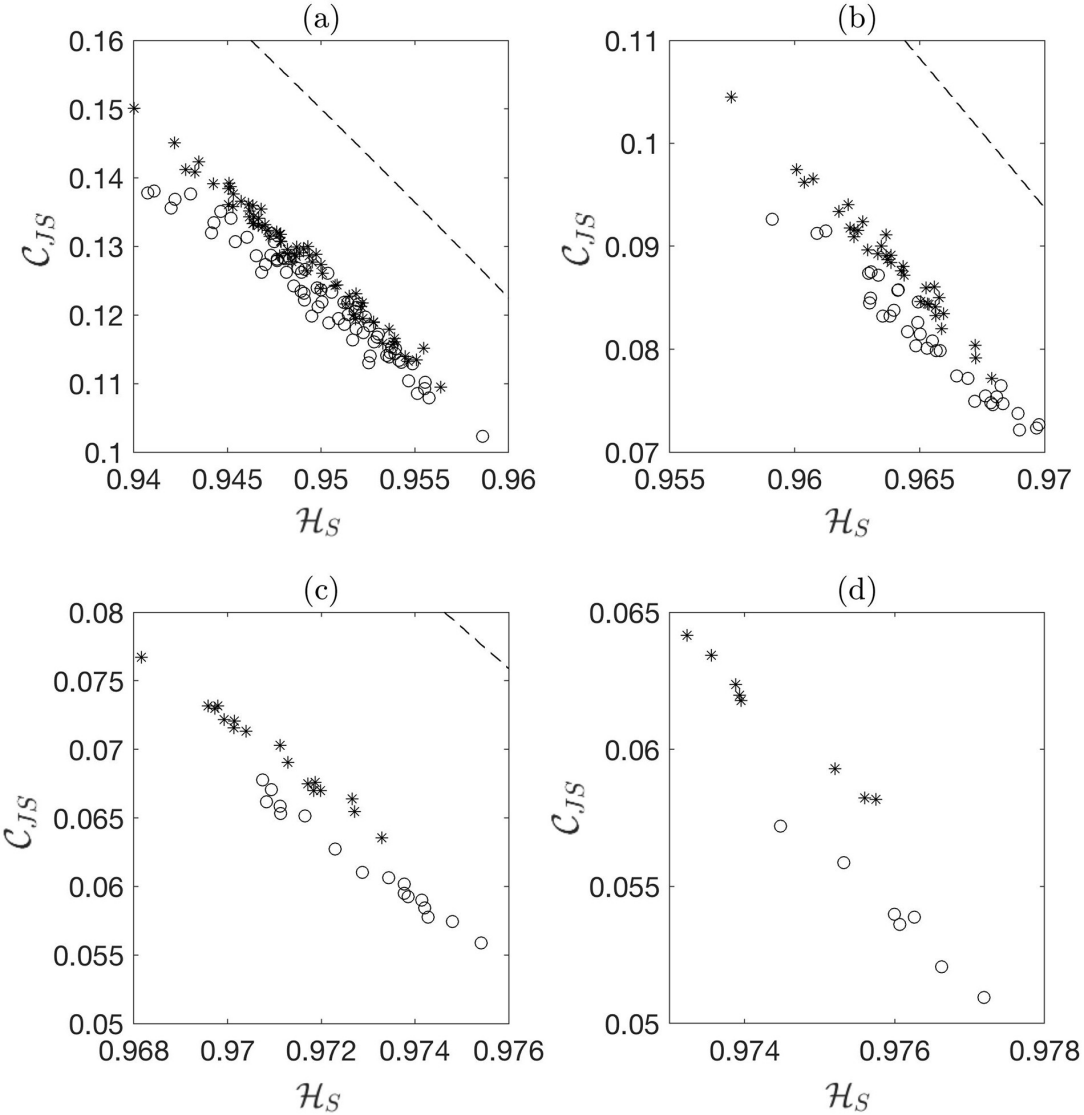
Complexity $C_{JS}$ vs. Permutation Entropy $H_{S}$ feature plane for different values of subsample length *N* for the case of 45.35 kg (100 lb) load and 25 rps (1500 rpm) rotational speed: (a) *N* = 2048, (b) *N* = 4096, (c) *N* = 8192, and (d) *N* = 16384; the CECP parameters are set to *D* = 6 and *τ* = 1. The stars represent the inner race fault and the circles represent outer race fault. The dashed lines represent the lower and upper limit curves. Some figures may not show the limit curves due to axis scale effects.

**Fig 4:**
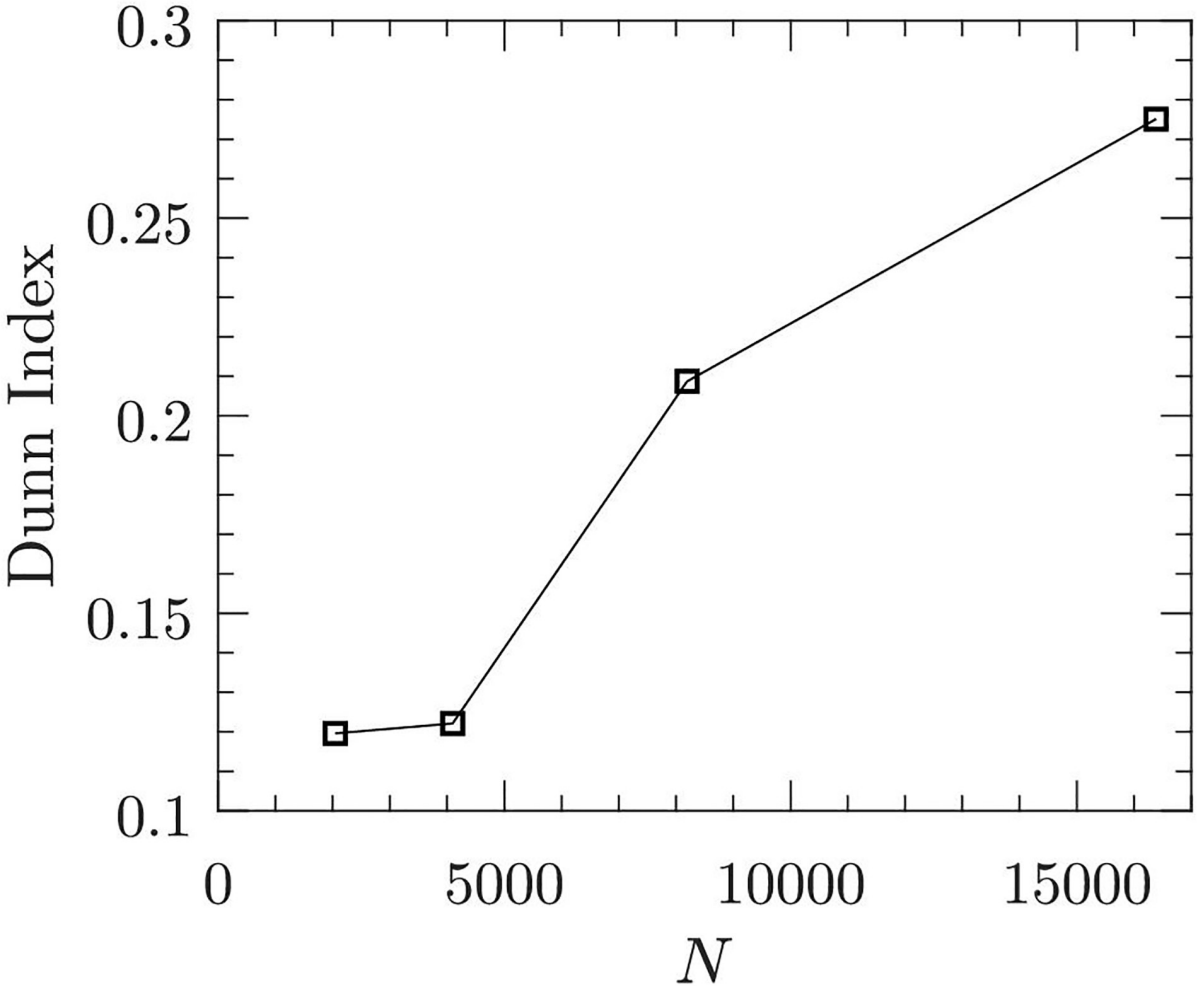
Effect of signal length *N* on Dunn Index. In this example, *N* = 2048, 4096, 8192, and 16384. In this case load = 45.35 kg (100 lb) and rotational speed = 25 rps (1500 rpm).

Fig 3 presents CECP maps for four different signal lengths. It is evident from the figure that the separation distance between fault classes widens and the variance of $H_{S}$ of each class decreases with increasing signal length. This leads to improvement in Dunn index of cluster formation as the signal length increases (see Fig 4). Dunn index, which is the ratio of minimum inter-cluster distance to maximum intra-cluster distance [62], measures the quality of cluster formations: the higher the Dunn index the better the cluster quality. Dunn index for *m* clusters is defined as

$$\text{Dunn}\;\text{Index}=\frac{{\text{min}}_{1\leq i<j\leq m}d\left(C_{i},C_{j}\right)}{{\text{max}}_{1\leq k\leq m}diam\left(C_{k}\right)}$$ (7)

where *d*(*C*_*i*_, *C*_*j*_) is dissimilarity (inter-cluster) between clusters *C*_*i*_ and *C*_*j*_. It is defined as $d\left(C_{i},C_{j}\right)={\text{min}}_{a\in C_{i},b\in C_{j}}E\left(a,b\right)$, where *E*(**a**, **b**) is the Euclidean distance between points **a** and **b**. $diam\left(C_{k}\right)={\text{max}}_{a,b\in C_{k}}E\left(a,b\right)$ is the diameter of cluster *C*_*k*_.

As mentioned in the earlier section, *D* < 4 is not desired for mechanical vibration signals since the permutation entropy is not able to detect exact dynamic changes. On the other hand *D* > 8 is computationally expensive. Similarly, *τ* > 5 is not recommended for vibration signals. We performed sensitivity analysis for all the load conditions by varying *N*, *D*, and *τ*. The results of the sensitivity analysis are presented in S1 File (see Figs S1-S12). We set the parameter values as follows: *N* = 2048, 4096, 8192, 16384, 32768; *D* = 3, 4, 5, 6; and *τ* = 1, 2, 3, 4, 5. In general, CECP analysis is employed for characterizing time series, which is sensitive to parameter choices. However, in the current work we are more interested in using CECP representation for class separation (or classification accuracy) with a correct set of application specific parameters. From the sensitivity analysis we found that for load conditions 11.33 kg and 22.67 kg, parameters *D* = 4, 5, 6, and *τ* = 1, 2, 3, 4 are suitable for achieving good class separability. Similarly, for load conditions 45.35 kg, 68.03 kg, and 90.71 kg, parameters *D* = 4, 5, 6, and *τ* = 1, 4 are appropriate and for load conditions 113.39 kg, and 136.07 kg, parameters *D* = 4, 5, 6, and *τ* = 1, 2, 3 are appropriate. To verify beyond visual observation of separability, we used a SVM to see how the classification accuracy improves with increasing signal length. For demonstration purpose we used *D* = 6 and *τ* = 1. Note that for practical purposes Bandt and Pompe [35] recommended 3 ≤ *D* ≤ 7 and *τ* = 1. We used receiver operating characteristic curve (ROC), area under curve (AUC), and classification accuracy (ACC) to evaluate the performance of the SVM classifier. We employed a linear SVM model with 5-fold cross validation. The results of the SVM are given Table ST1 in S1 File. Figs S13-S15 in S1 File present ROC, AUC and ACC plots. We observed that for all load conditions, the SVM classifier performance improves with increasing signal length, which is consistent with the results of the earlier analysis using Dunn Index.

### Bearing dataset from CWRU

**Fig 5:**
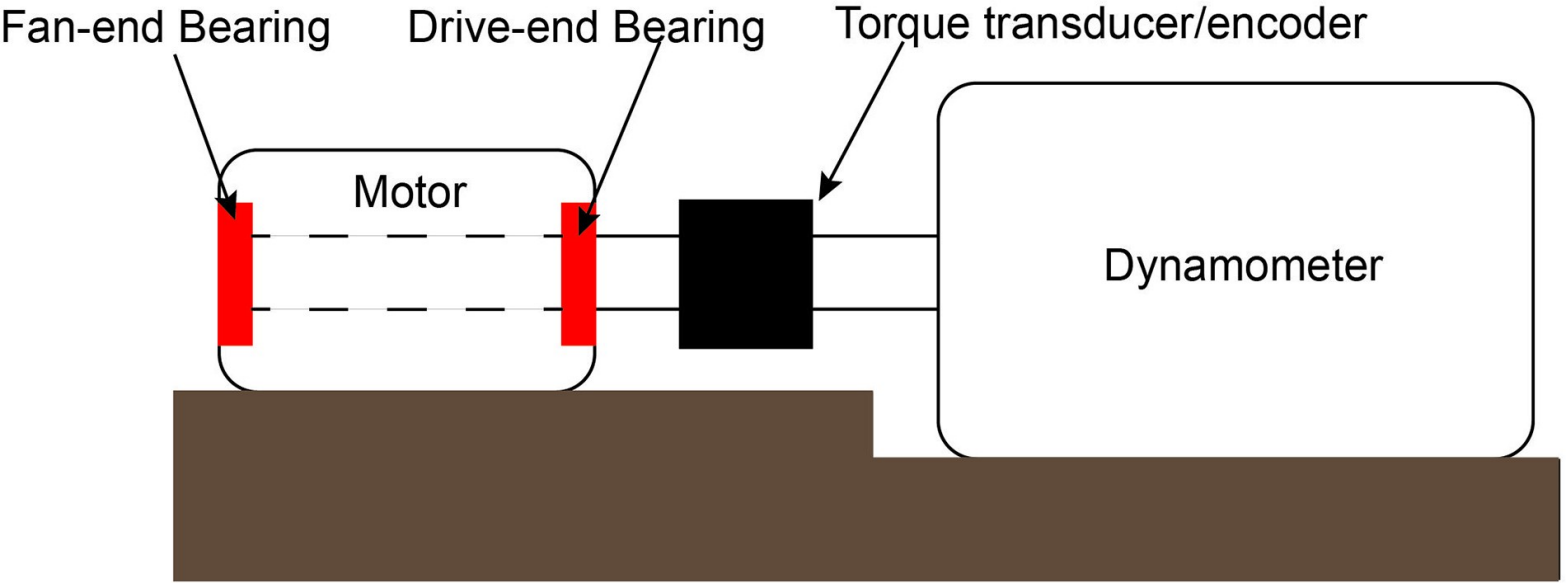
Schematic of CWRU experimental setup for bearing fault data.

Case Western Reserve University (CWRU) bearing dataset [63] includes high quality signals collected at normal and faulty conditions of bearings. Fig 5 shows the setup of the experiment. The testbed consists of a 2-hp motor (left side), a torque transducer/encoder (center), and a dynamometer (right side).

The setup has test bearings located at the drive-end and the fan-end of the motor. The faults were introduced in the inner race and outer race and on a ball for both drive-end bearings and the fan-end bearings using electro-discharge machining.

**Fig 6:**
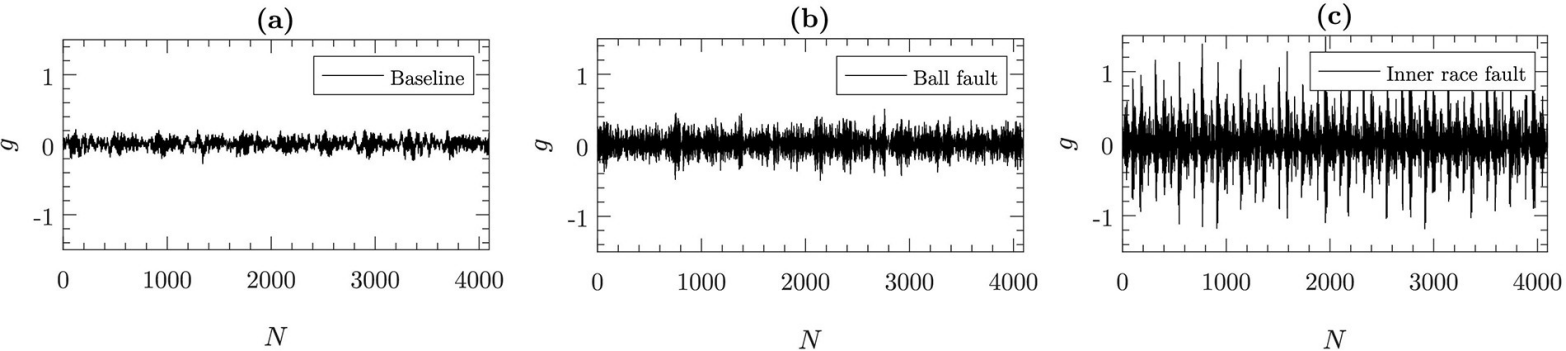
Sample vibration signals representing (a) baseline condition, (b) ball fault, and (c) inner race fault. In this case, the fault diameter for both the ball and inner race is 0.17 mm (0.007 inches), load is 0 kW (0 hp), and rotational speed is 29.95 rps (1797 rpm). The bearing considered is drive-end bearing.

The accelerometers attached to the motor housing using magnetic bases were used to measure vibration signals from the setup. One accelerometer was attached on the drive-end of the motor and another on the fan-end of the motor. For some experiments, an additional accelerometer was attached to the base plate supporting the motor. Vibration signals were collected using a 16-channel DAT recorder. Sensor signals were collected at a frequency of 12,000 Hz. The length of the baseline signals (i.e., signal collected from components at normal condition) was varied between 200,000 and 500,000 data points and the length of fault-related signals was varied between 120,000 and 130,000 data points. Fig 6 shows the sample vibration signals representing the baseline condition, ball fault and inner race fault. The fault characteristics of the CWRU dataset are exhaustively studied in time and frequency domains by Smith et al. [64]. We performed Augmented Dickey-Fuller (ADF) test to verify the stationarity of the signals. For signals in all the three cases (baseline, inner race fault and ball fault) we obtain a *p* value of 0.01 < 0.05 confirming the stationarity of the signals.

Speed and horsepower data were hand-recorded from the torque transducer/encoder. In this case we analyzed only baseline signals, inner race fault signals, and ball fault signals. The experimental parameters are outlined in Table 2. For all the parameter variations, the fault depth was maintained constant at 0.2794 mm (0.011 inches).

**Table 2:** Drive-end bearing and fan-end bearing data for analysis [63].

Fault Diameter (mm)	Motor Load (kW)	Approx. Motor Speed (rps)
0.17 mm (0.007”)	0	29.95 rps (1797 rpm)
	0.73 kW (1 hp)	29.53 rps (1772 rpm)
	1.47 kW (2 hp)	29.16 rps (1750 rpm)
	2.20 kW (3 hp)	28.83 rps (1730 rpm)
0.35 mm (0.014”)	0	29.95 rps (1797 rpm)
	0.73 kW (1 hp)	29.53 rps (1772 rpm)
	1.47 kW (2 hp)	29.16 rps (1750 rpm)
	2.20 kW (3 hp)	28.83 rps (1730 rpm)
0.53 mm (0.021”)	0	29.95 rps (1797 rpm)
	0.73 kW (1 hp)	29.53 rps (1772 rpm)
	1.47 kW (2 hp)	29.16 rps (1750 rpm)
	2.20 kW (3 hp)	28.83 rps (1730 rpm)

We performed sensitivity analysis for all the load conditions by varying *N*, *D*, and *τ*. The results of the sensitivity analysis for a selected set of operating conditions are given in Figs S16-S23 in S1 File. We set the parameter values as follows: *N* = 2048, 4096, 8192, 16384, 32768; *D* = 3, 4, 5, 6; and *τ* = 1, 2, 3, 4, 5. From the sensitivity analysis we noticed that for all operating conditions given in Table 2, for both fan-end and drive-end bearing, *D* = 4, 5, 6 and *τ* = 1, 2 are suitable parameters to obtain good class separability. For both drive-end and fan-end bearings the baseline condition exhibit higher complexity and lower permutation entropy compared to inner race and ball fault conditions. We observed that the fan-end bearing exhibits bigger class separation between baseline and faulty conditions than the drive-end bearing.

To confirm the visual observation of class separability, we used a SVM to see how the classification accuracy improves with increasing signal length. For demonstration purpose we used *D* = 6 and *τ* = 1. We used a linear SVM model with 5-fold cross validation. The results of the SVM for fan-end bearing are given in Table ST3 in S1 File. The results of the SVM for drive-end bearing are given in Table ST4 in S1 File. Figs S24-S25 in S1 File present ROC, AUC and ACC plots. Similar to the results of the MFPT experiment, we observed that for almost all operating conditions the SVM classifier performance improves with increasing signal length.

### Gear dataset from the PHM society

**Fig 7:**
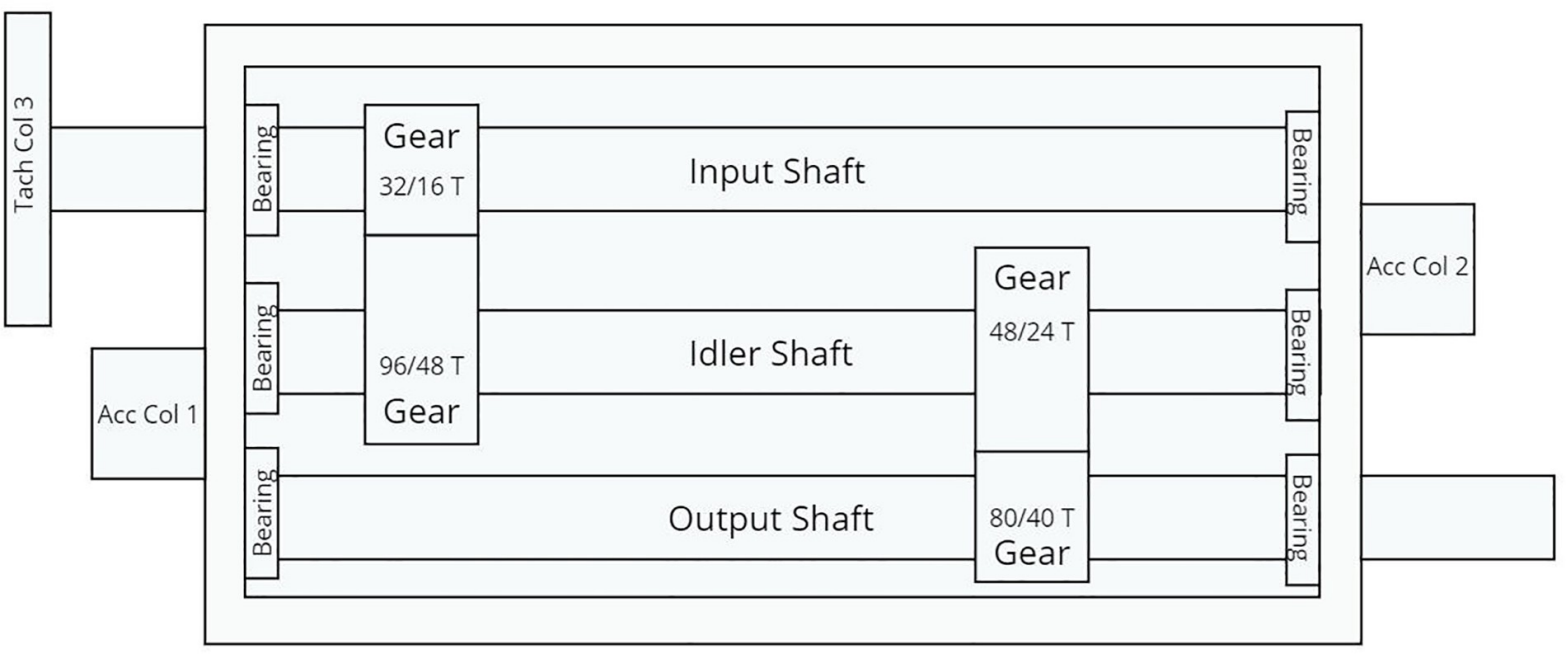
Experimental setup for gear fault detection [65].

We considered a dataset provided by the PHM society that contains labeled data on different types of gear degradation [65]. The experiments were conducted using spur gears and helical gears. For CECP application, we considered experiments with helical gears. Fig 7 shows the experimental setup. The setup is common for both spur gears and helical gears. Fig 7 shows the details of gear teeth for both spur and helical gears. For our analysis we considered only the helical gears.

The setup has an input shaft, an idler shaft and an output shaft on which the gears are mounted. The input side is on the left and the output side is on the right. Two accelerometers are mounted, one on the input side and the other on the output side. The left helical gear on the input shaft has 16 teeth and the left helical gear on the idler shaft has 48 teeth. The right helical gear on idler shaft has 24 teeth and the right helical gear on the output shaft has 40 teeth. Experiments with helical gears were performed six times. All in all, there are two fault categories (chipped tooth and broken tooth) and one baseline category (no known faults).

**Fig 8:**
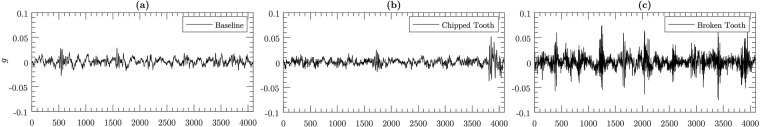
Sample signal representing (a) baseline condition, (b) chipped tooth condition, and (c) broken tooth condition. The sample signals shown here are taken from experimental condition of high load and 50 rps (3000 rpm) rotational speed.

From the PHM dataset we picked the case labled helical 1 which has no known gear defects as the *baseline* case. We selected helical 2 which has a chipped tooth in helical gear with 24 teeth as a *chipped tooth* gear case and helical 5 which has a broken tooth in helical gear with 24 teeth as a *broken tooth* gear case. In all the three cases, we used the vibration signals recorded from accelerometer 2 placed on the output side. The signals were recorded under two different load conditions labeled as low and high and five different rotational speeds, i.e. 30 rps (1800 rpm), 35 rps (2100 rpm), 40 rps (2400 rpm), 45 rps (2700 rpm), and 50 rps (3000 rpm). For each of these settings, two signals were recorded for four seconds each. Thus, for one fault signal, 533,312 data points were generated in eight-second recording at a sampling rate of 66,666 samples per second. Fig 8 shows sample signals of length 4096 data points each representing the baseline and the fault conditions. We performed Augmented Dickey-Fuller (ADF) test to verify the stationarity of the signals. For all the three cases (baseline, chipped tooth and broken tooth) we obtain a *p* value of 0.01 < 0.05 confirming the stationarity of the signals.

**Fig 9:**
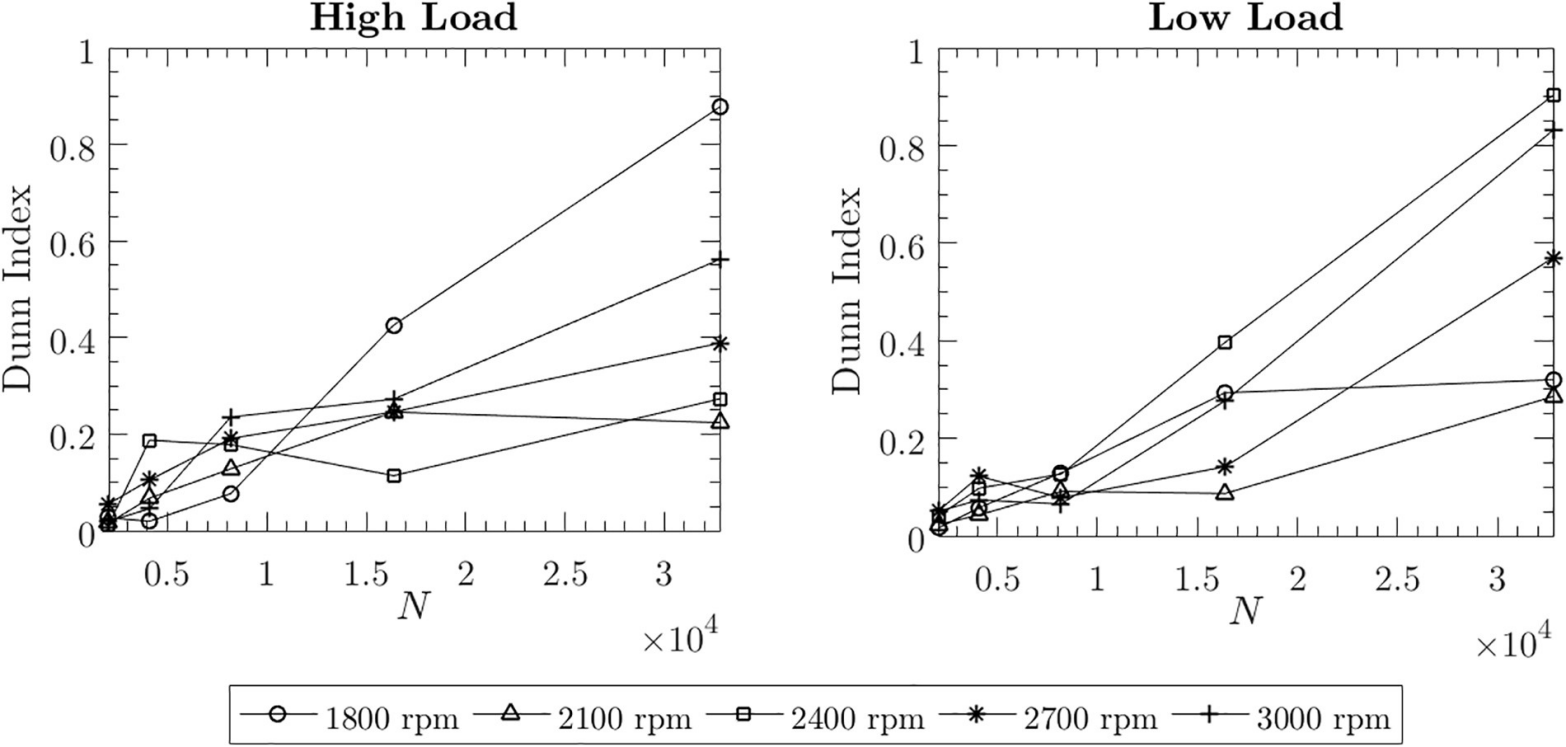
Variation of Dunn Index values with respect to varying signal length. *N* takes values 2048, 4096, 8192, 16384, and 32768. The plots give Dunn Index values for low and high load conditions and different rotational speeds, i.e.30 rps (1800 rpm), 35 rps (2100 rpm), 40 rps (2400 rpm), 45 rps (2700 rpm), and 50 rps (3000 rpm).

We performed sensitivity analysis for all the load conditions by varying *N*, *D*, and *τ*. The results of the sensitivity analysis for a selected set of operating conditions are given in Figs S26-S41 in S1 File. We set the parameter values as follows: *N* = 2048, 4096, 8192, 16384, and 32768, *D* = 3, 4, 5, and 6, and *τ* = 1, 2, 3, 4, and 5. We observed that *D* = 4, 5 and *τ* = 1, 5 are suitable for obtaining good class separability. In addition to visual observation of class separability, we used a SVM to see how the classification accuracy improves with respect to the increasing signal length. For demonstration purpose we used *D* = 6 and *τ* = 1. We used a linear SVM model with 5-fold cross validation. The results of the SVM for low and high load conditions are given in Table ST5 in S1 File. Figs S42-S45 in S1 File give ROC, AUC and ACC plots. Similar to the results of the MFPT and CWRU experiments, we observed that for almost all operating conditions the SVM classifier performance improves with increasing signal length. The same pattern is observed when using the Dunn Index values across all operating conditions (see Fig 9).

## Conclusion

PHM enables effective preventive maintenance (PM), reliability centered maintenance (RCM), and condition based maintenance (CBM) of mechanical components. Advancement in sensor technology, machine learning algorithms, and computing technology have contributed to the advancement of PHM. In a PHM framework, both diagnostics and prognostics use data-driven models for fault detection of machine components. In this approach, sensor signals need a good representation that enables accurate detection of faults and health condition of machine components. The results confirm that CECP representation enables accurate classification of different faults in bearings and gears. It is observed that CECP representation is able to handle signal lengths larger than those demonstrated in existing studies. In case of CECP representation, as the signal length increases, so does the Dunn index and SVM classification accuracy. In other words, as the signal length increases, the ability of CECP representation to detect changes in health condition of components improves. We observed that the optimum parameter values for CECP representation depends on the application. For bearing applications we found that embedding dimension *D* = 4, 5, 6, and embedding delay *τ* = 1, 2, 3 are suitable for good fault classification accuracy. For gear applications, *D* = 4, 5, and delay *τ* = 1, 5 are suitable for good fault classification results. For signal length of 16,384 data points, the fault classification accuracy varies from 90% to 100% for bearing applications, and from 85% to 100% for gear applications. Given that CECP representation has only two parameters, not only can it be used for predictive analytics but also for visualization of sensor signals in a 2-dimensional plane. While predictive models can used for optimizing maintenance decisions, visualization can be used for creating dashboards for monitoring health condition of machine components. From class separability perspective, the CECP repesentation is able to generate linearly separable classes for the classification of different fault states. Beyond separability characteristics, there are several statistical tests that can be performed for permutation entropy and complexity [66, 67] as needed in individual applications.

Real-world manufacturing PHM applications often involve unreliable connectivity in cloud computing. These applications require high bandwidth and cost for transferring data to the cloud. They suffer from high latency which is not desirable for closed-loop interaction between machine state and actuation. In addition, the data transfer and processing operations are subject to compliance, regulation, and cyber security constraints. These constraints create a need for localized edge computing, which pushes the intelligence, processing power, and communication capabilities of an edge gateway directly into devices like PACs (programmable automation controllers). Intelligent PACs collect, analyze, and process data from the physical assets they are tethered to while they run the control system program at the same time. In such an edge computing environment, a signal representation like CECP is highly desirable because of its compactness, lean computation complexity, robustness to different types of signals, and good predictive performance.

## Supporting information

S1 File Sensitivity analysis on CECP parameters and signal length. (PDF)
