# Supplementary material for: Complexity and entropy representation for machine component diagnostics
Source: PLoS One. 2019 Jul 9;14(7):e0217919. doi: 10.1371/journal.pone.0217919 (PMC6615599; doi:10.1371/journal.pone.0217919)
Supplement: S1 File — (PDF) [file pone.0217919.s001.pdf]

S1 Supporting Information: Sensitivity Analysis on CECP Parameters and Signal Length

1.0 Numerical Computations

1.1 $\mathcal{H}_S$ and $\mathcal{C}_{JS}$

The numerical computations of $\mathcal{H}_S$ and $\mathcal{C}_{JS}$ were performed using R computational software. The ordinal pattern distribution is computed using codebook() function from pdc library. The permutation entropy values are computed using permutation_entropy() function from statcomp library. The complexity values are computed using MPR_complexity() function from statcomp library. The upper limit and lower limit curves are computed using limit_curves() function from statcomp library.

1.2 Support Vector Machine (SVM) Calculations

The SVM computations were performed using Matlab computational software. fitcsvm() function was used for binary class problem. Linear SVM model was chosen for the fault classification. The 5 fold cross validation was opted for validation. Receiver Operating Characteristic (ROC), Area Under Curve (AUC), and Accuracy (ACC) were used to evaluate the performance of the

classifier. Thr ROC and AUC computations was performed using perfcurve() function. The validation accuracy (ACC) was computed using kfoldLoss() function. For three class problem fitcecoc() function was used to model a linear SVM. The validation process and evaluation of the classifier performance remains the same as above.

2.0 CECP Plots for MFPT Ball Bearing Experiment

Vibration signals for inner race and outer race fault of roller bearings were generated. The details and the data source is given in the main text.

Operating Conditions

Constant rotational speed of 1500 rpm and seven different load conditions: 11.3398, 22.6796 45.3592, 68.0388, 90.7184, 113.3980, and 136.0776 kgs (or25, 50, 100, 150, 200, 250, and 300 lbs). Each vibration signal is recorded for 3 seconds at 48,828 Hz frequency; it resulted in a signal of length 146,484data points.

Sensitivity Analysis

For each load condition we vary the parameters as given below

Signal Length $n = 2048, 4096, 8192, 16384, 32768,$

Embedding Dimension $D = 3, 4, 5, 6$, and,

Embedding Delay $\tau = 1, 2, 3, 4, 5$.

In the following sections we plot $\mathcal{H}_S$ and $\mathcal{C}_{JS}$ values for load conditions 25, 100 and 300 lb. We also plot the performance of SVM classifiers for the aforementioned three load conditions. For the SVM classification, we consider parameter values $D = 6$ and $\tau = 1$. For SVM we consider signal lengths, $n = 2048, 4096, 8192, 16384$ for training. We did not consider signal length

$n = 32768$ because it yields fewer data points not sufficient for training SVM.

2.1 Sensitivity Analysis

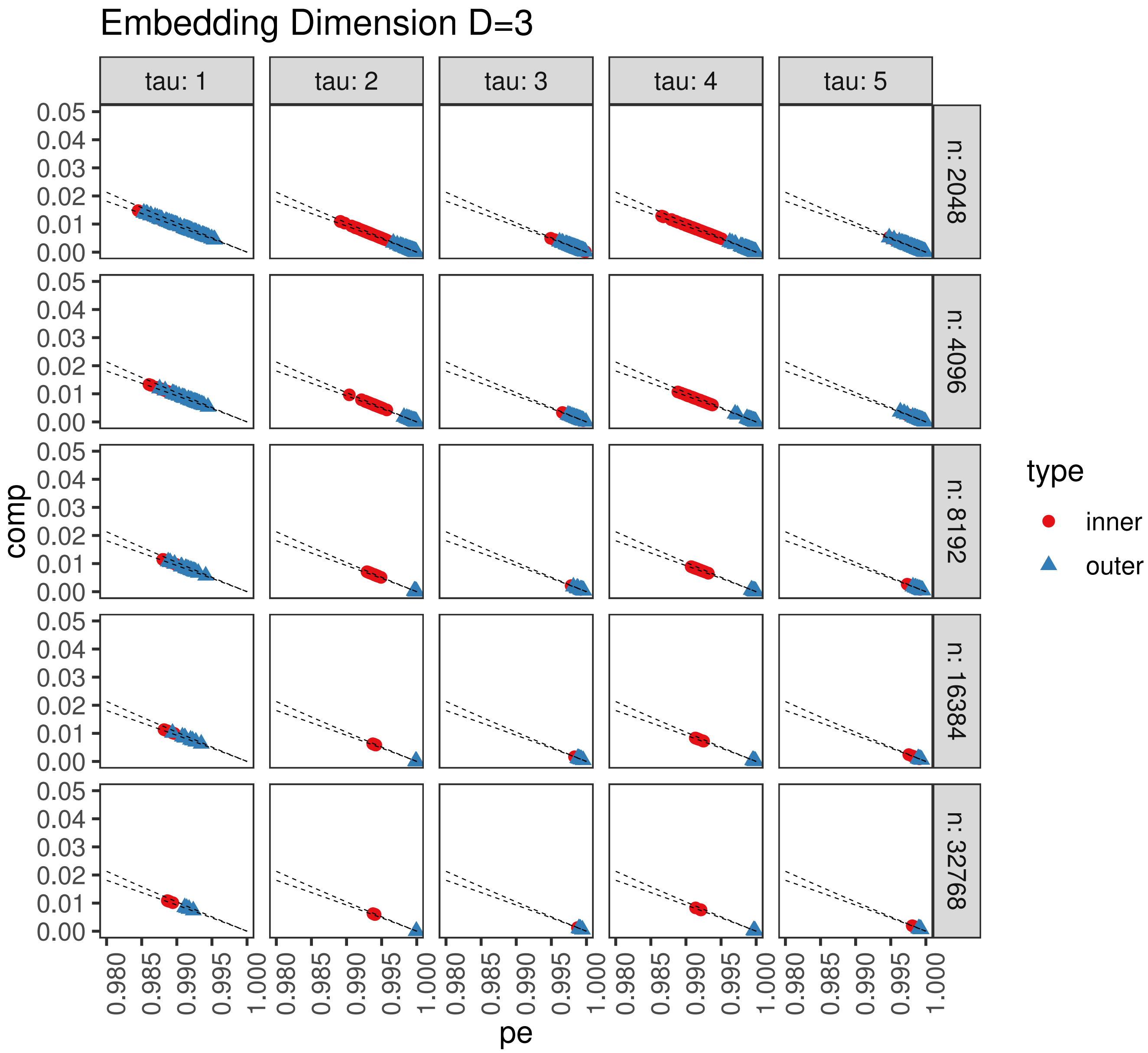

Figure S1: Load = 11.3398 kgs (25 lb), Rotational speed = 1500 rpm. The dashed lines represent the lower and upper limit curves. $D = 3$

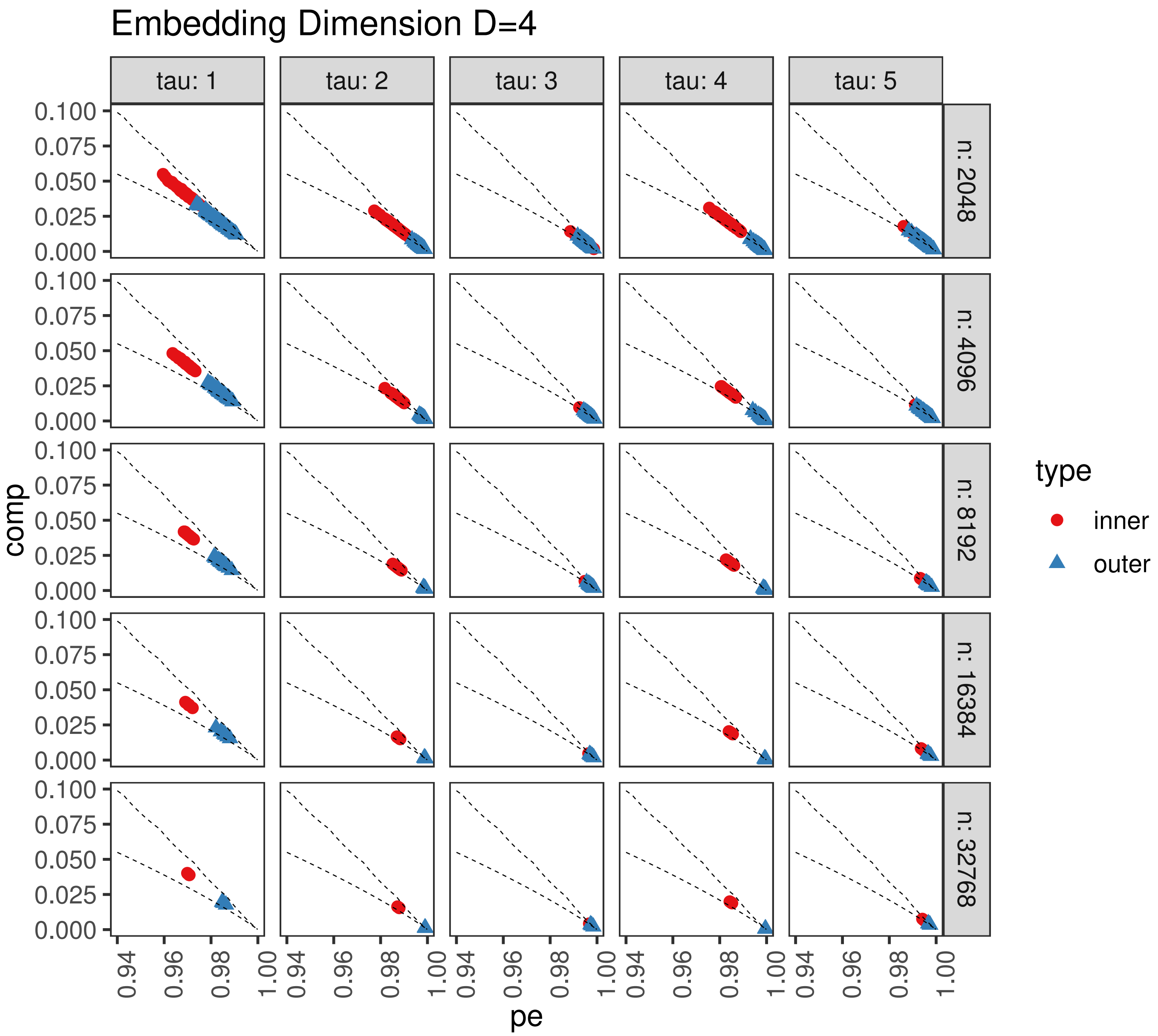

Figure S2: Load = 11.3398 kgs (25 lb), Rotational speed = 1500 rpm. The dashed lines represent the lower and upper limit curves. $D = 4$

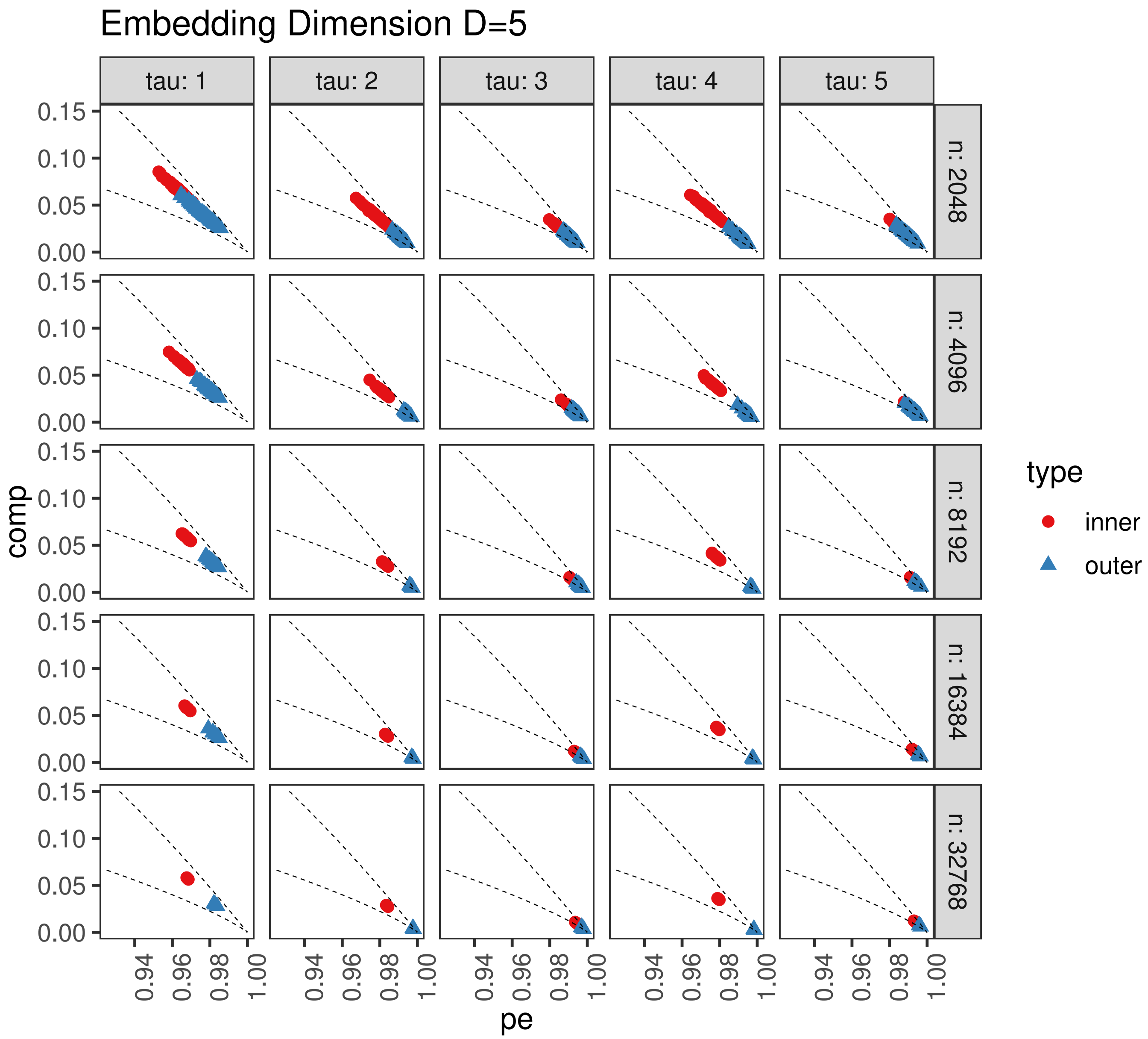

Figure S3: Load = 11.3398 kgs (25 lb), Rotational speed = 1500 rpm. The dashed lines represent the lower and upper limit curves. $D = 5$

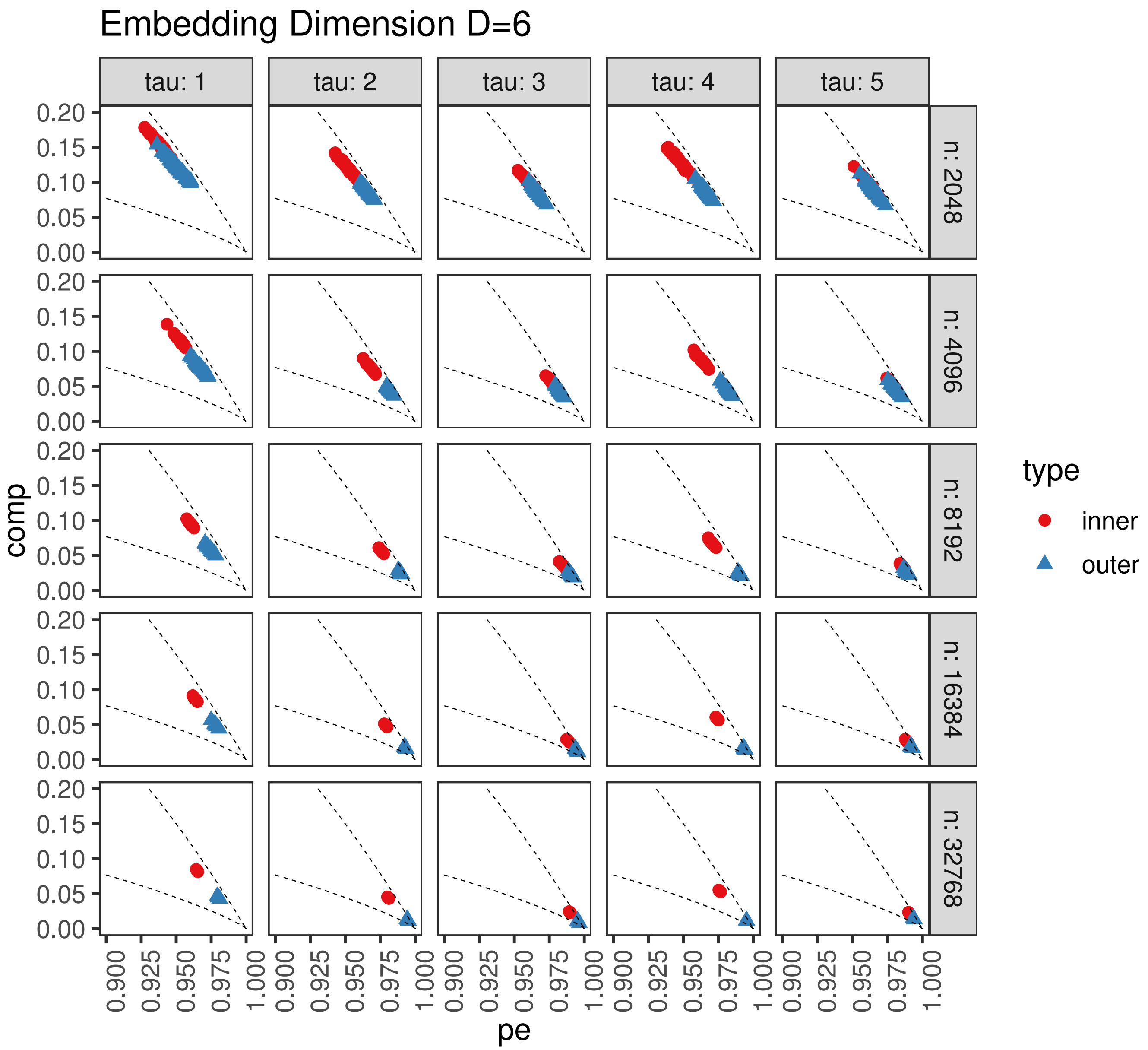

Figure S4: Load = 11.3398 kgs (25 lb), Rotational speed = 1500 rpm. The dashed lines represent the lower and upper limit curves. $D = 6$

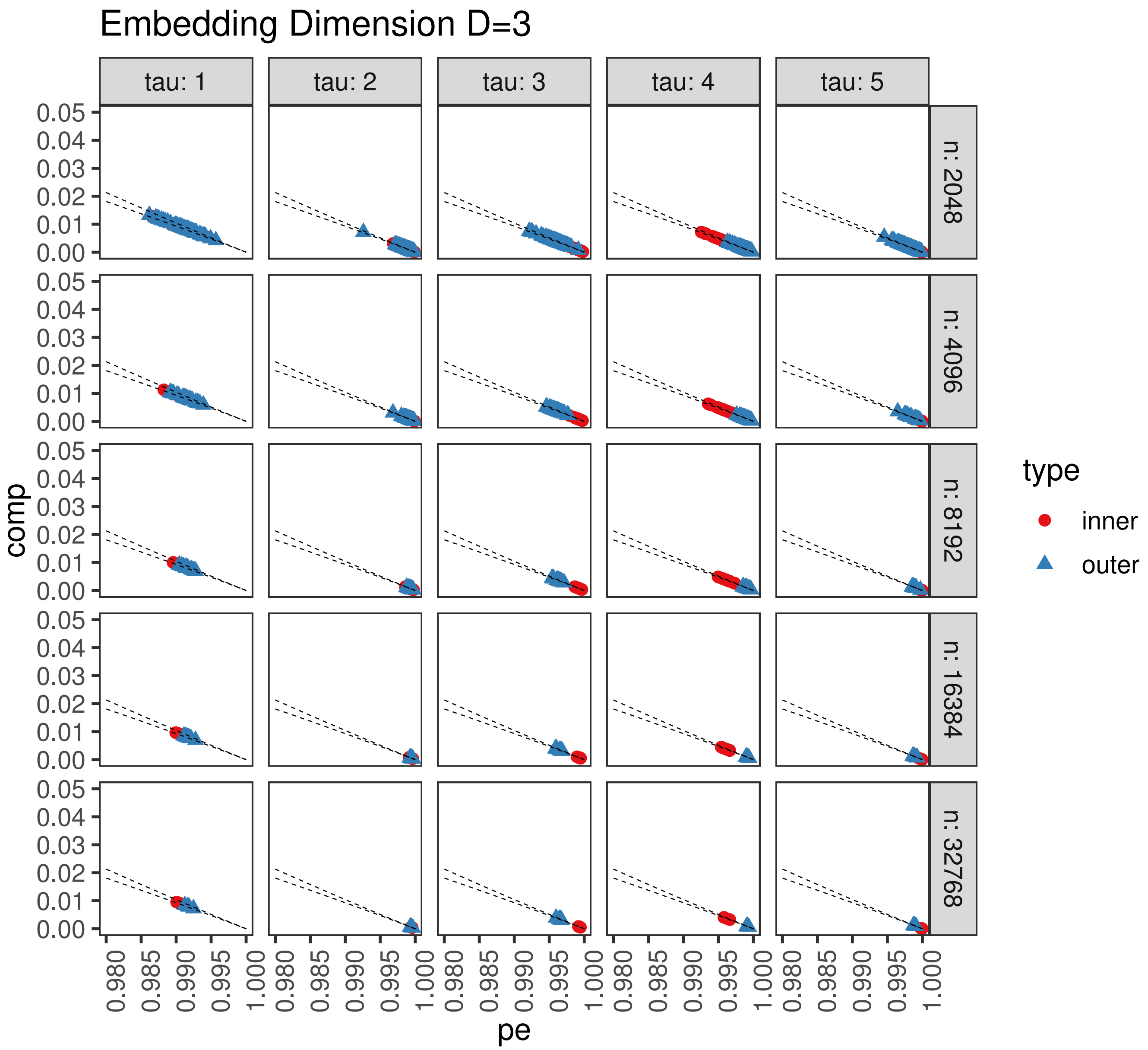

Figure S5: Load = 45.3592 kgs (100 lb), Rotational speed = 1500 rpm. The dashed lines represent the lower and upper limit curves. $D = 3$

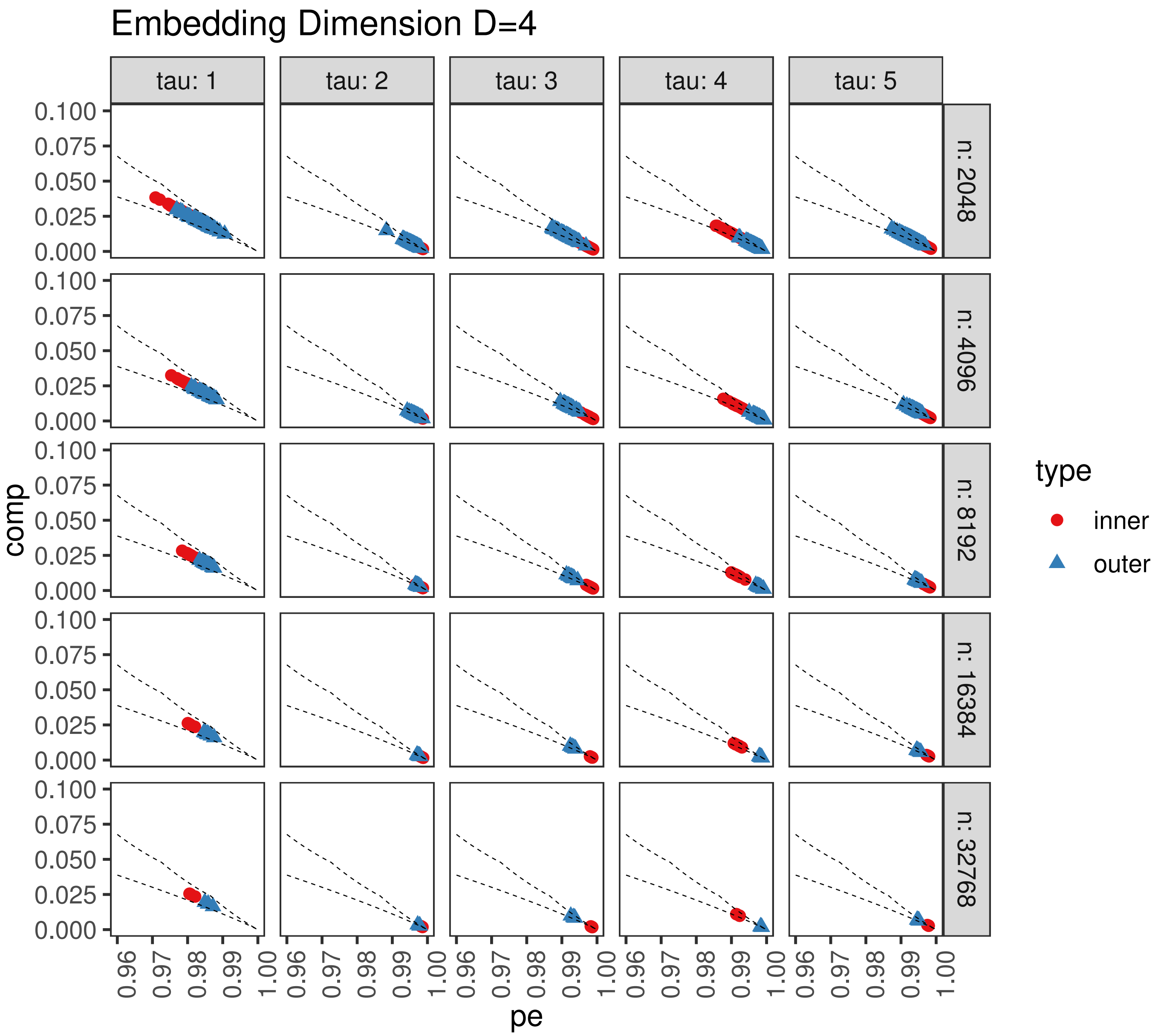

Figure S6: Load = 45.3592 kgs (100 lb), Rotational speed = 1500 rpm. The dashed lines represent the lower and upper limit curves. $D = 4$

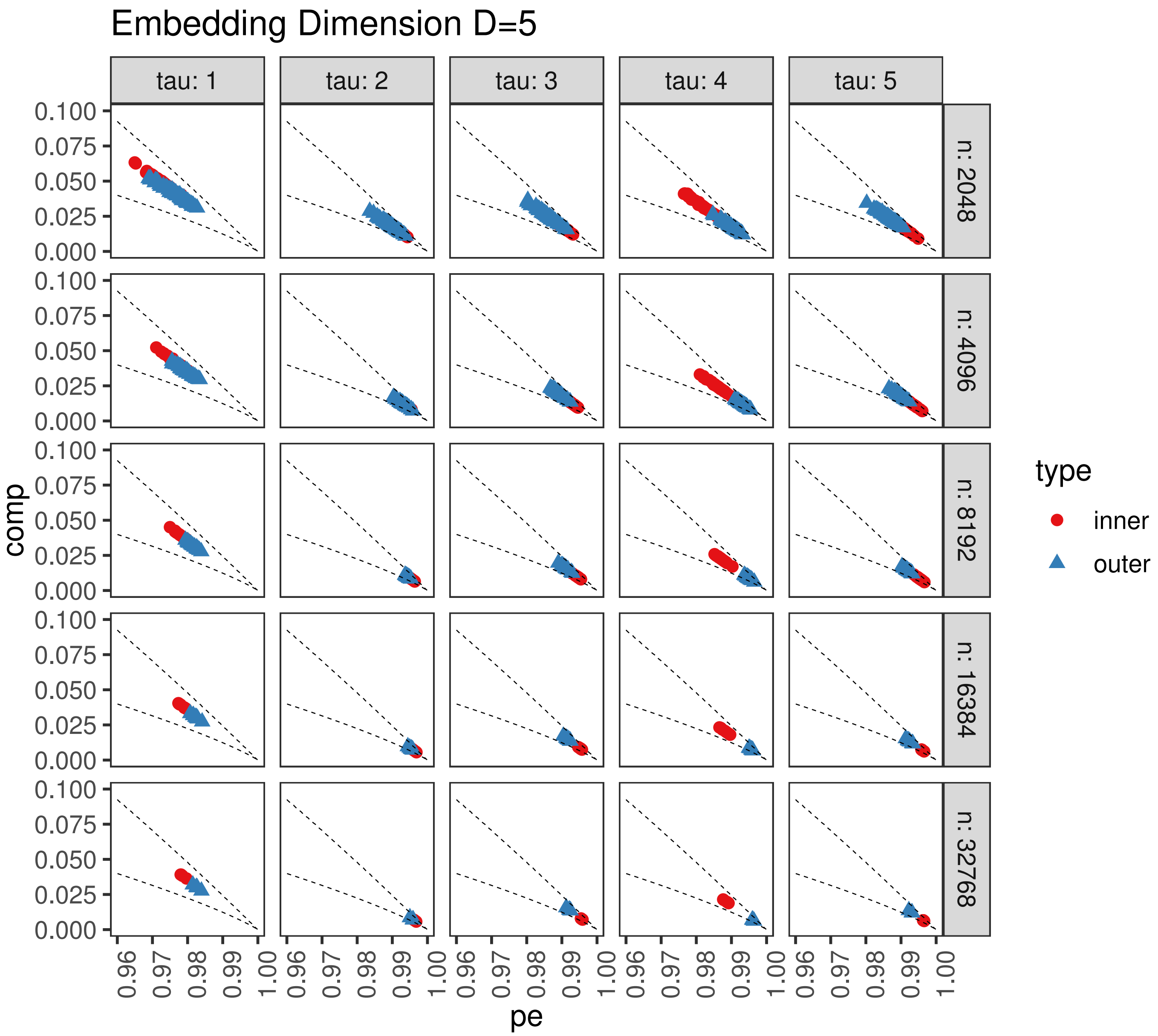

Figure S7: Load = 45.3592 kgs (100 lb), Rotational speed = 1500 rpm. The dashed lines represent the lower and upper limit curves. $D = 5$

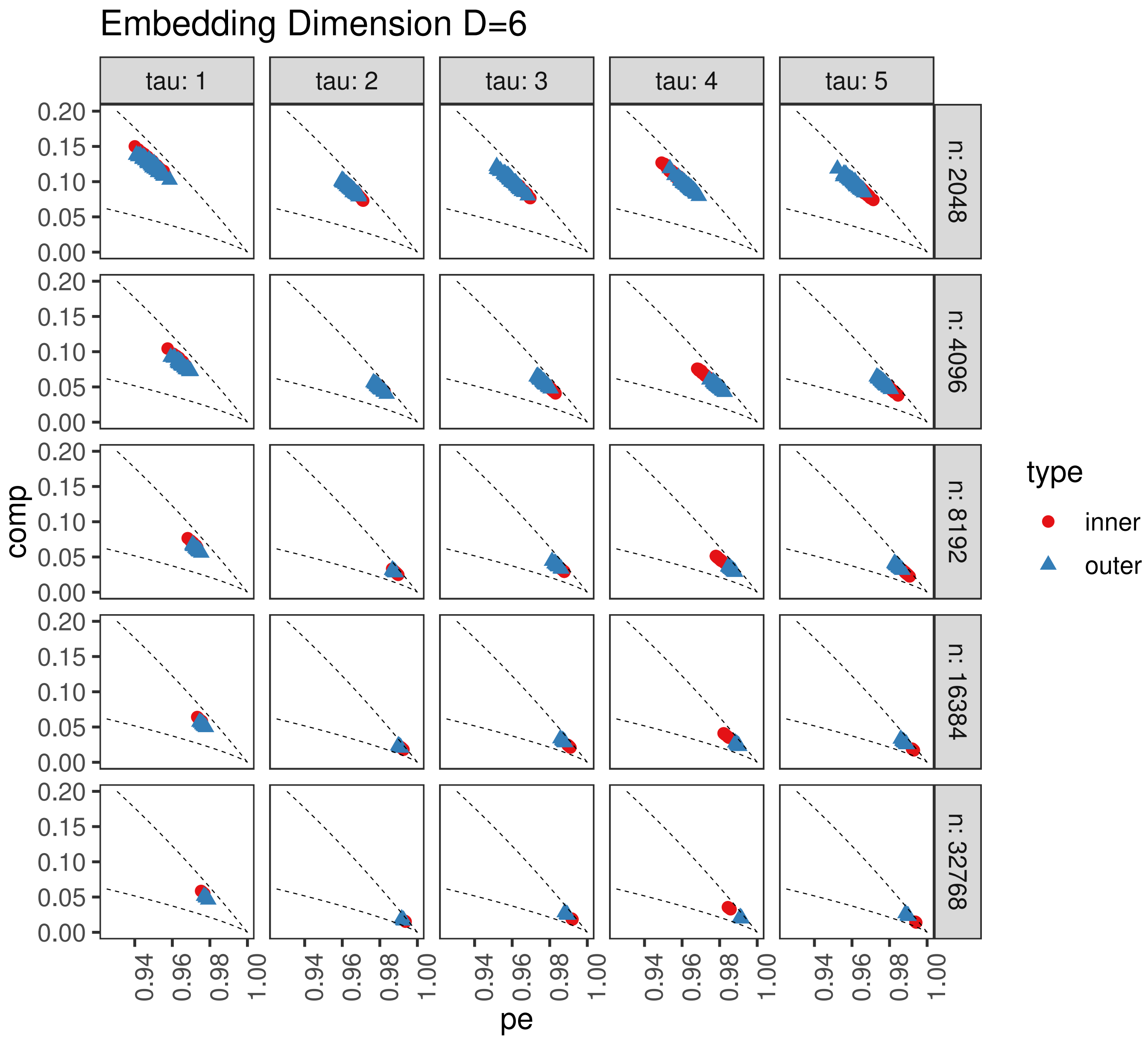

Figure S8: Load = 45.3592 kgs (100 lb), Rotational speed = 1500 rpm. The dashed lines represent the lower and upper limit curves. $D = 6$

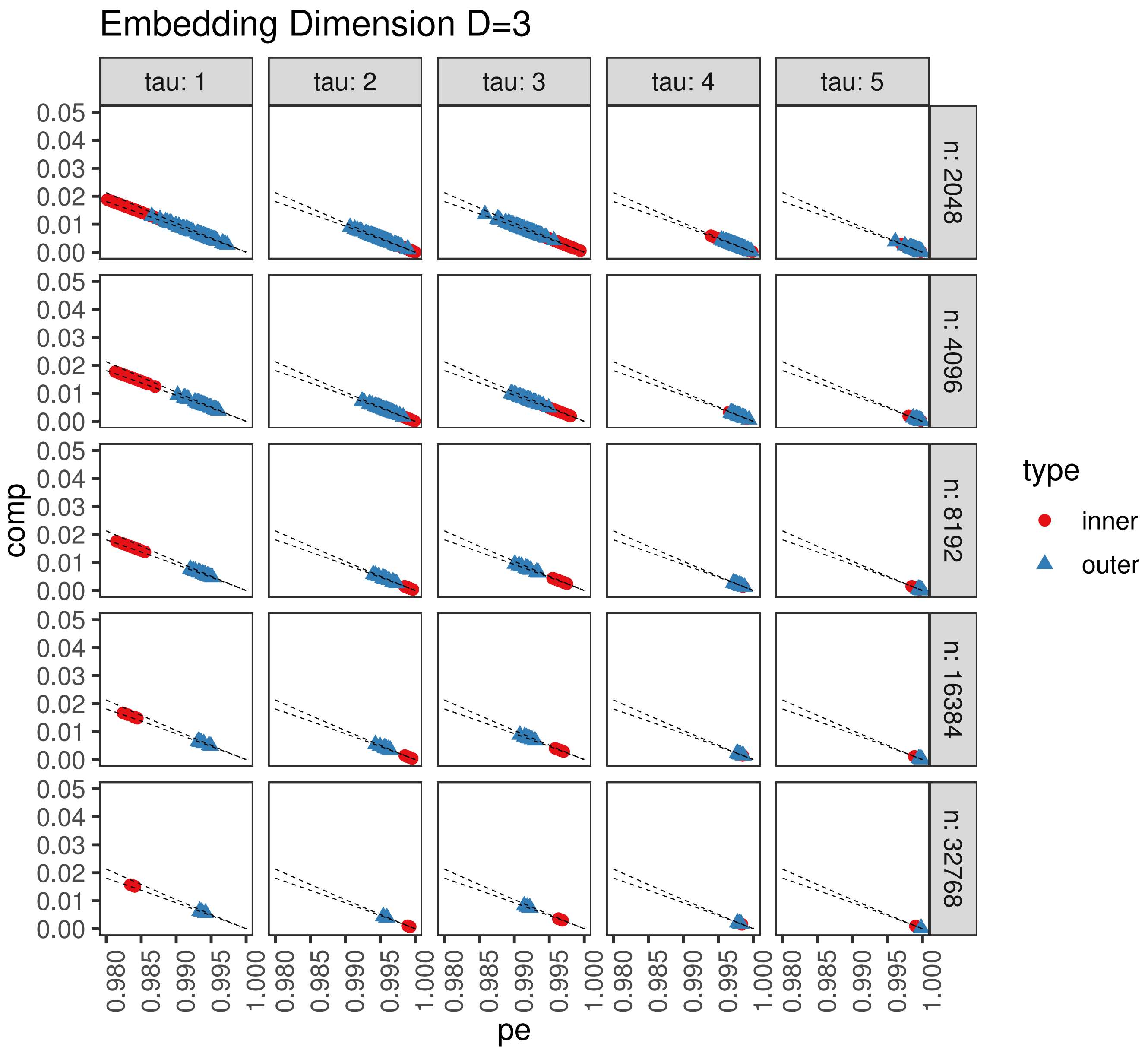

Figure S9: Load = 136.077 kgs (300 lb), Rotational speed = 1500 rpm. The dashed lines represent the lower and upper limit curves. $D = 3$

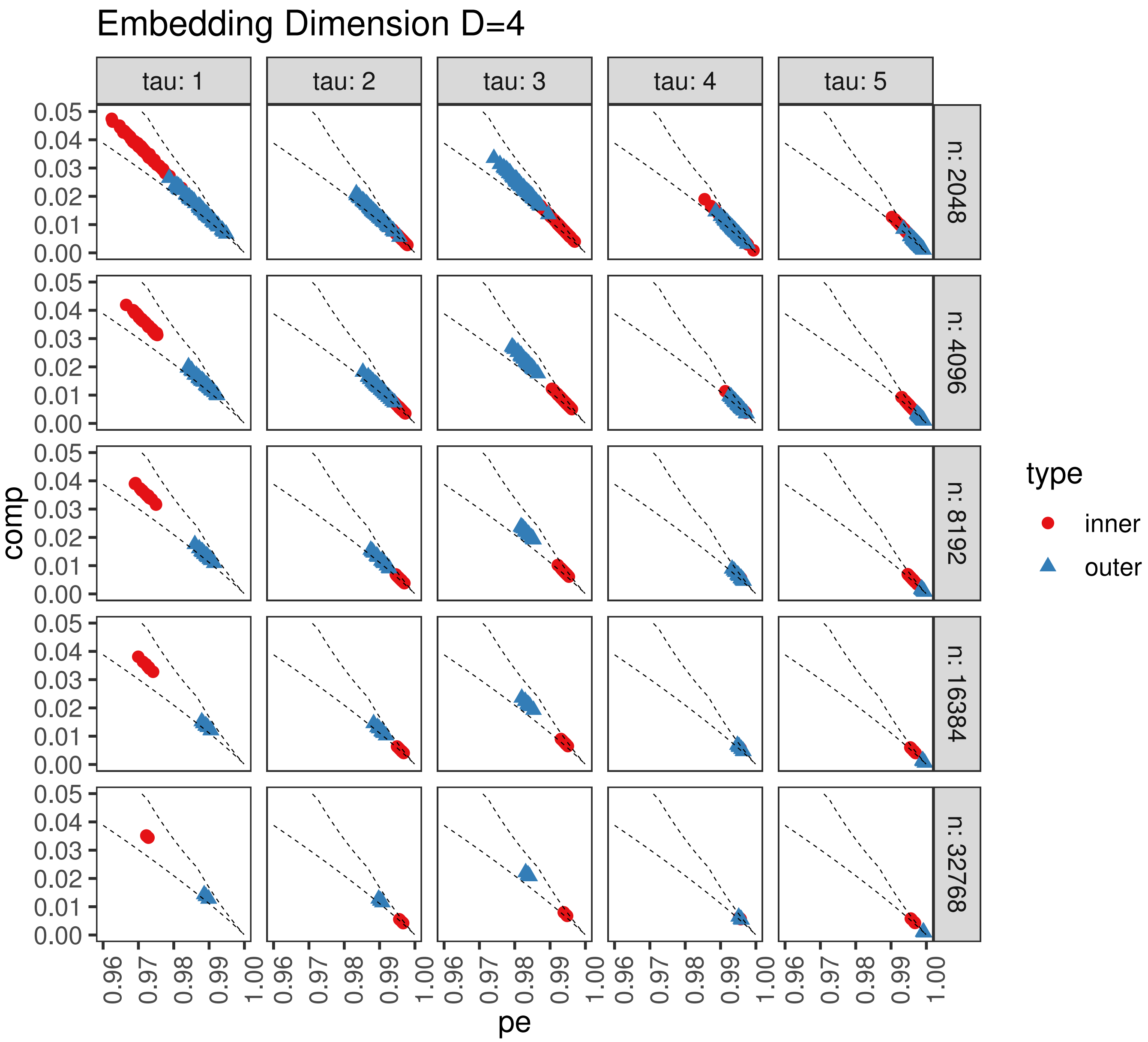

Figure S10: Load = 136.077 kgs (300 lb), Rotational speed = 1500 rpm. The dashed lines represent the lower and upper limit curves. $D = 4$

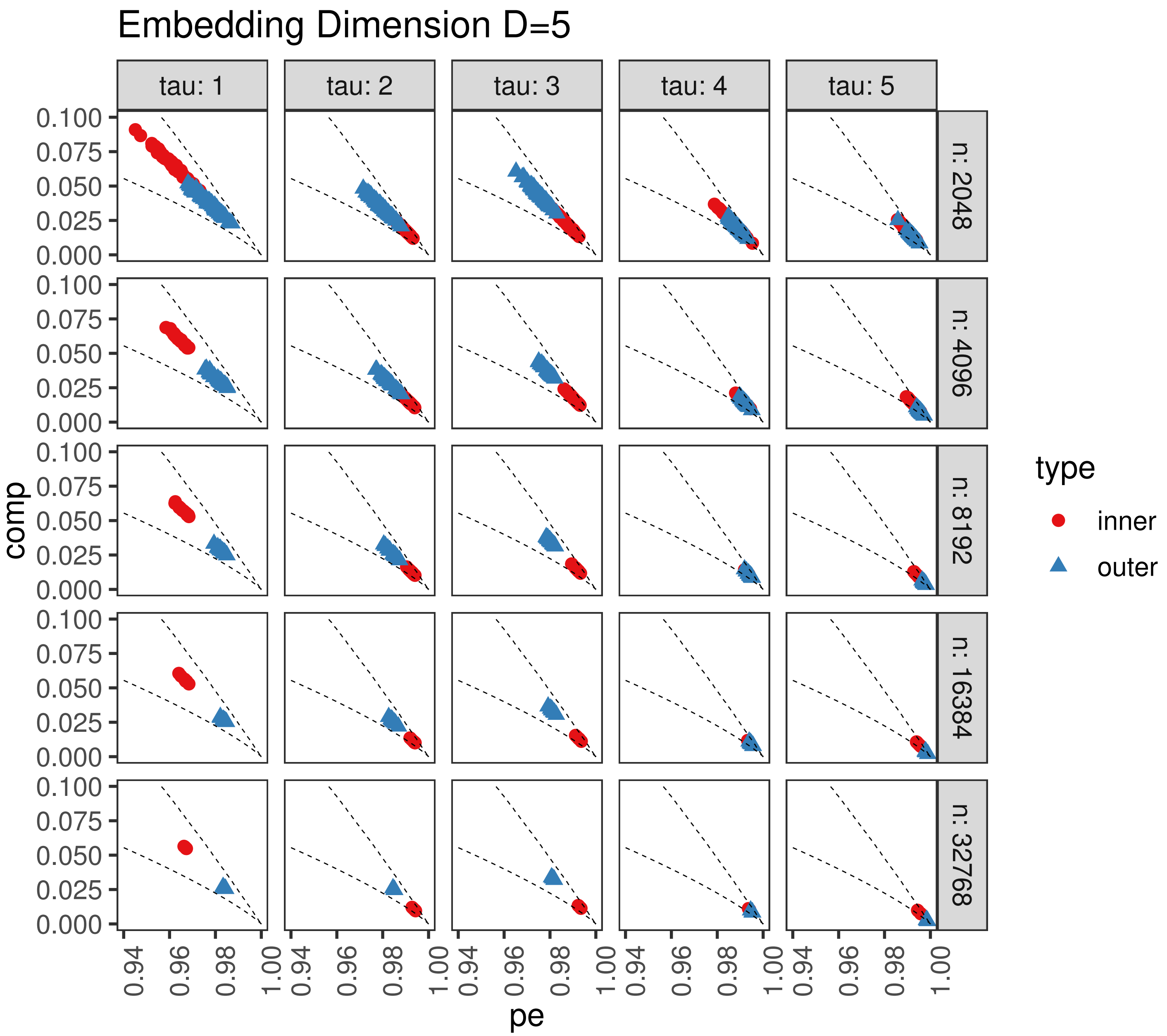

Figure S11: Load = 136.077 kgs (300 lb), Rotational speed = 1500 rpm. The dashed lines represent the lower and upper limit curves. $D = 5$

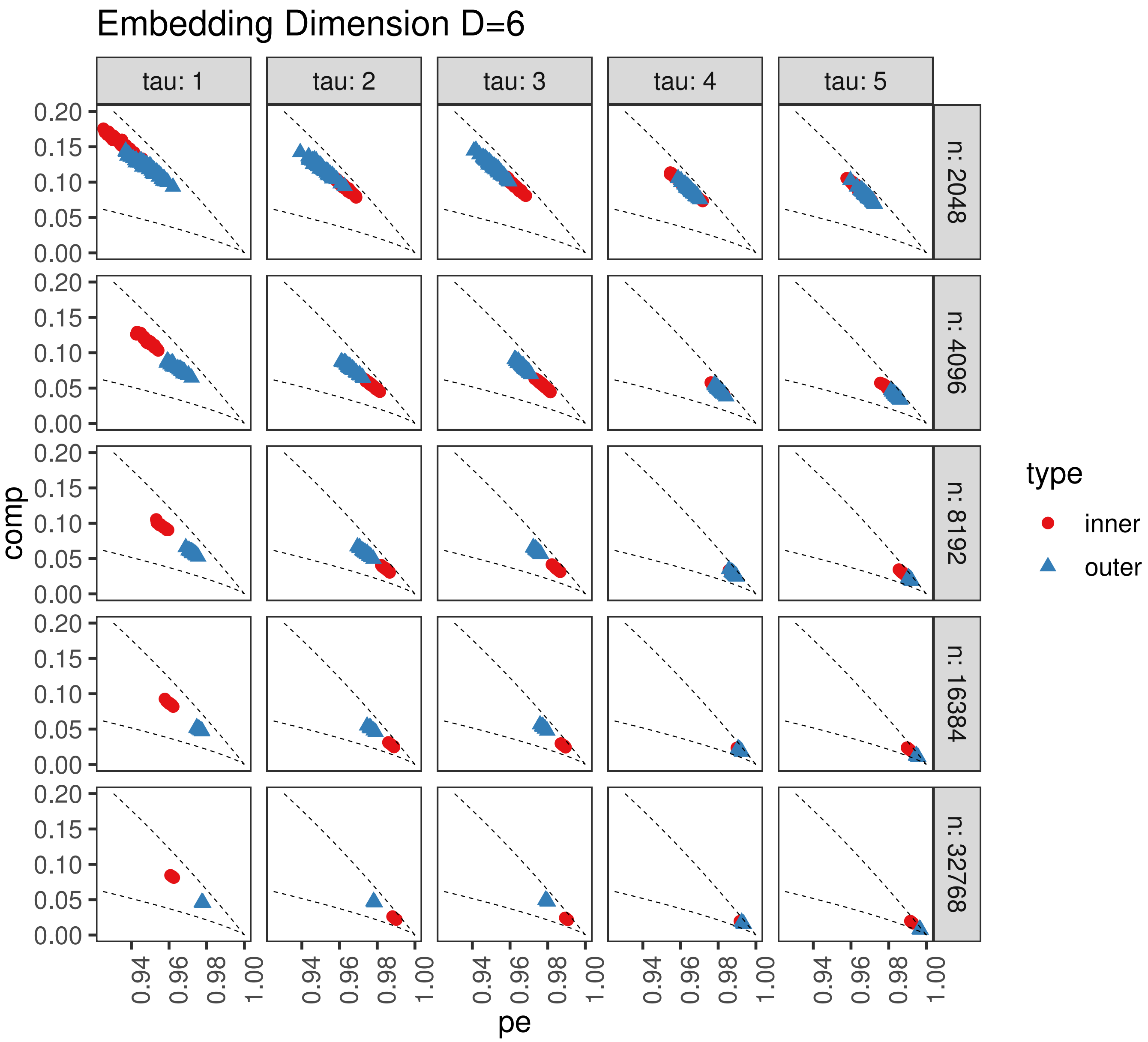

Figure S12: Load = 136.077 kgs (300 lb), Rotational speed = 1500 rpm. The dashed lines represent the lower and upper limit curves. $D = 6$

2.2 SVM Classifier Performance for load conditions 25, 100, and 300 lb

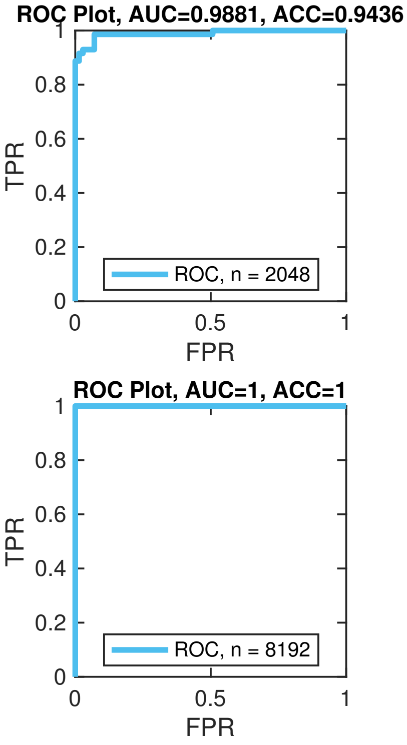

Figure S13: SVM results for Load = 11.3398 kgs (25 lb), Rotational speed = 1500 rpm, $D = 6$, and $\tau = 1$

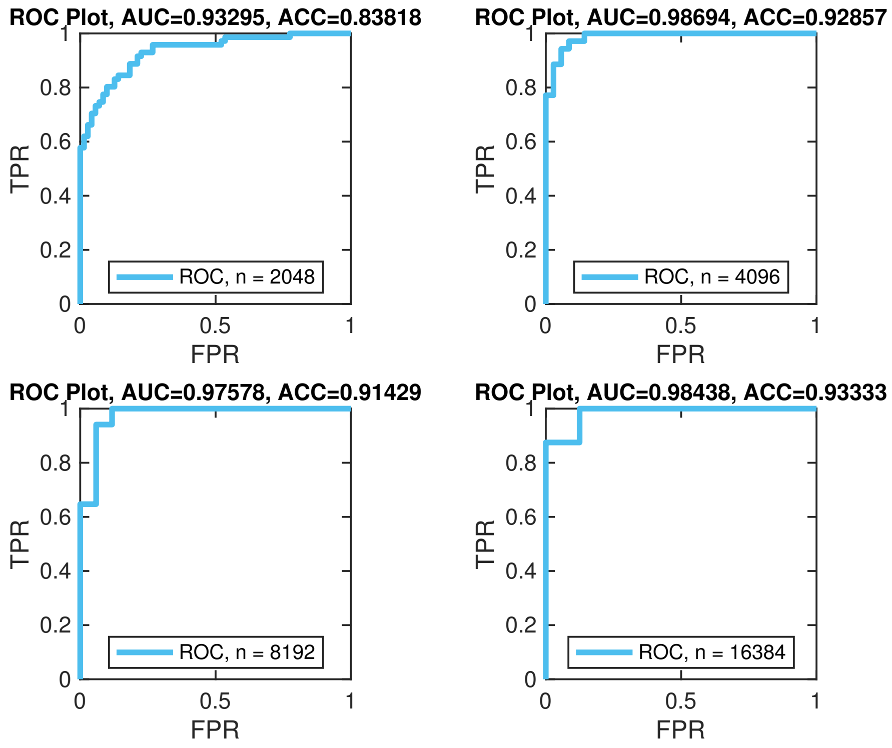

Figure S14: SVM results for Load = 45.3592 kgs (100 lb), Rotational speed = 1500 rpm, $D = 6$, and $\tau = 1$

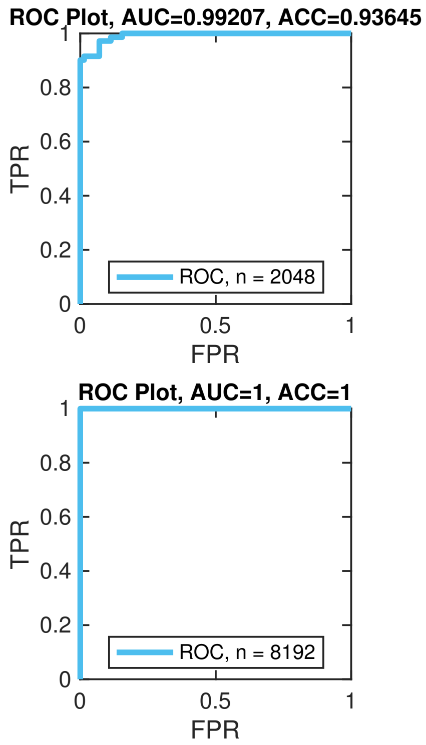

Figure S15: SVM results for Load = 136.077 kgs (300 lb), Rotational speed = 1500 rpm, $D = 6$, and $\tau = 1$

2.3 Classifier Accuracy for all Operating Conditions

We have included the results of the sensitivity analysis and the SVM classification for three load conditions. We observe the same pattern for other load conditions where the classifier performance improves with respect to increase in the signal length. The plots for all conditions are not shown to conserve space. The SVM results for all the load conditions for $D = 6$ and $\tau = 1$ are presented in Table S1. Since the number of data points are very less for signal length $n = 32768$, we did not include in SVM calculations.

Table S1: SVM classification accuracy in percentage for different load conditions and signal length n for bearing fault classification (25 rps (1500 rpm) rotational speed). $D = 6$ and $\tau = 1$

**Load (kgs)**	Accuracy for $n = 2048$	Accuracy for $n = 4096$	Accuracy for $n = 8192$	Accuracy for $n = 16384$
11.3398	94.36%	100%	100%	100%
22.6796	95.07%	100%	100%	100%
45.3592	83.81%	92.85%	91.42%	93.33%
68.0388	86.62%	94.28%	100%	100%
90.7184	87.19%	94.28%	94.28%	100%
113.3980	90.17%	90.0%	97.14%	100%
136.0776	93.64%	100%	100%	100%

3.0 CECP Plots for CWRU Bearing Experiments

Vibration signals were collected for ball fault, inner race fault and good working condition (baseline) bearing. The details of the experiment is given in the main text. Sensor signals were collected at a frequency of 12,000 Hz. The length of each fault-related signals was varied between 120,000 and 130,000 data points while the length of the baseline signals was varied between 200,000 and 500,000 data points.

Operating Conditions

The experimental parameters are outlined in Table S2. For all the parameter variations the fault depth was maintained at 0.2794 mm (0.011 inches).

Table S2: Drive-end bearing and fan-end bearing operating conditions

Fault Diameter (mm)	Motor Load (kW)	Approx. Motor Speed (rps)
0.1778 mm (0.007")	0	29.95 rps (1797 rpm)
	0.7355 kW (1 hp)	29.53 rps (1772 rpm)
	1.471 kW (2 hp)	29.16 rps (1750 rpm)
	2.206 kW (3 hp)	28.83 rps (1730 rpm)
0.3556 mm (0.014")	0	29.95 rps (1797 rpm)
	0.7355 kW (1 hp)	29.53 rps (1772 rpm)
	1.471 kW (2 hp)	29.16 rps (1750 rpm)
	2.206 kW (3 hp)	28.83 rps (1730 rpm)
0.5334 mm (0.021")	0	29.95 rps (1797 rpm)
	0.7355 kW (1 hp)	29.53 rps (1772 rpm)
	1.471 kW (2 hp)	29.16 rps (1750 rpm)
	2.206 kW (3 hp)	28.83 rps (1730 rpm)

In the following sections we include the plots for one case of drive end bearing and one case of fan end bearing. Table ST3 includes the accuracy results for SVM classification for all operating conditions and parameters $D = 6$ and $\tau = 1$

3.1 Sensitivity Analysis

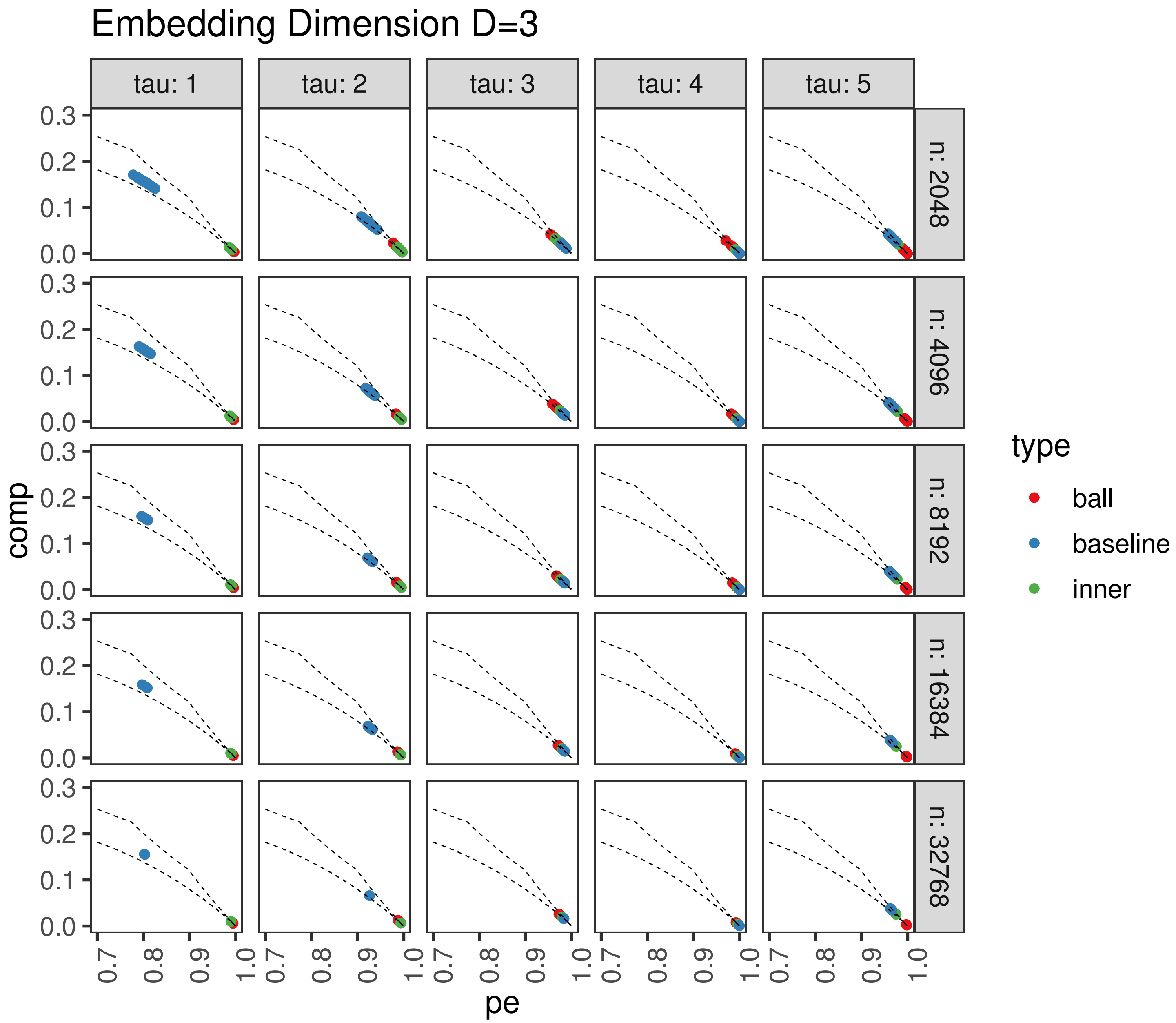

Figure S16: Fan end bearing, Load=0 hp, Fault Diameter=0.007 inches, Rotational Speed = 1797 rpm. The dashed lines represent the lower and upper limit curves. $D = 3$

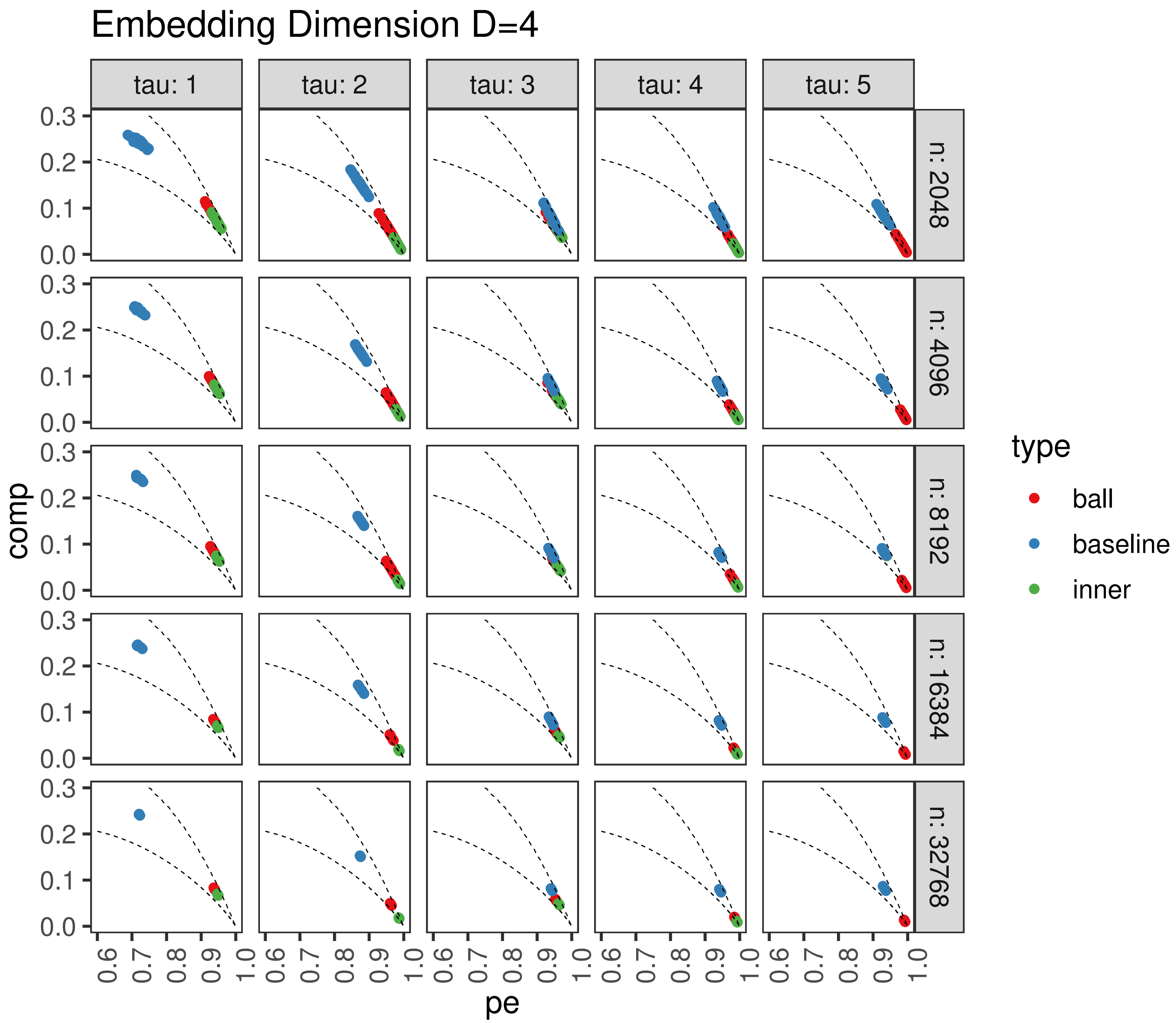

Figure S17: Fan end bearing, Load=0 hp, Fault Diameter=0.007 inches, Rotational Speed = 1797 rpm. The dashed lines represent the lower and upper limit curves. $D = 4$

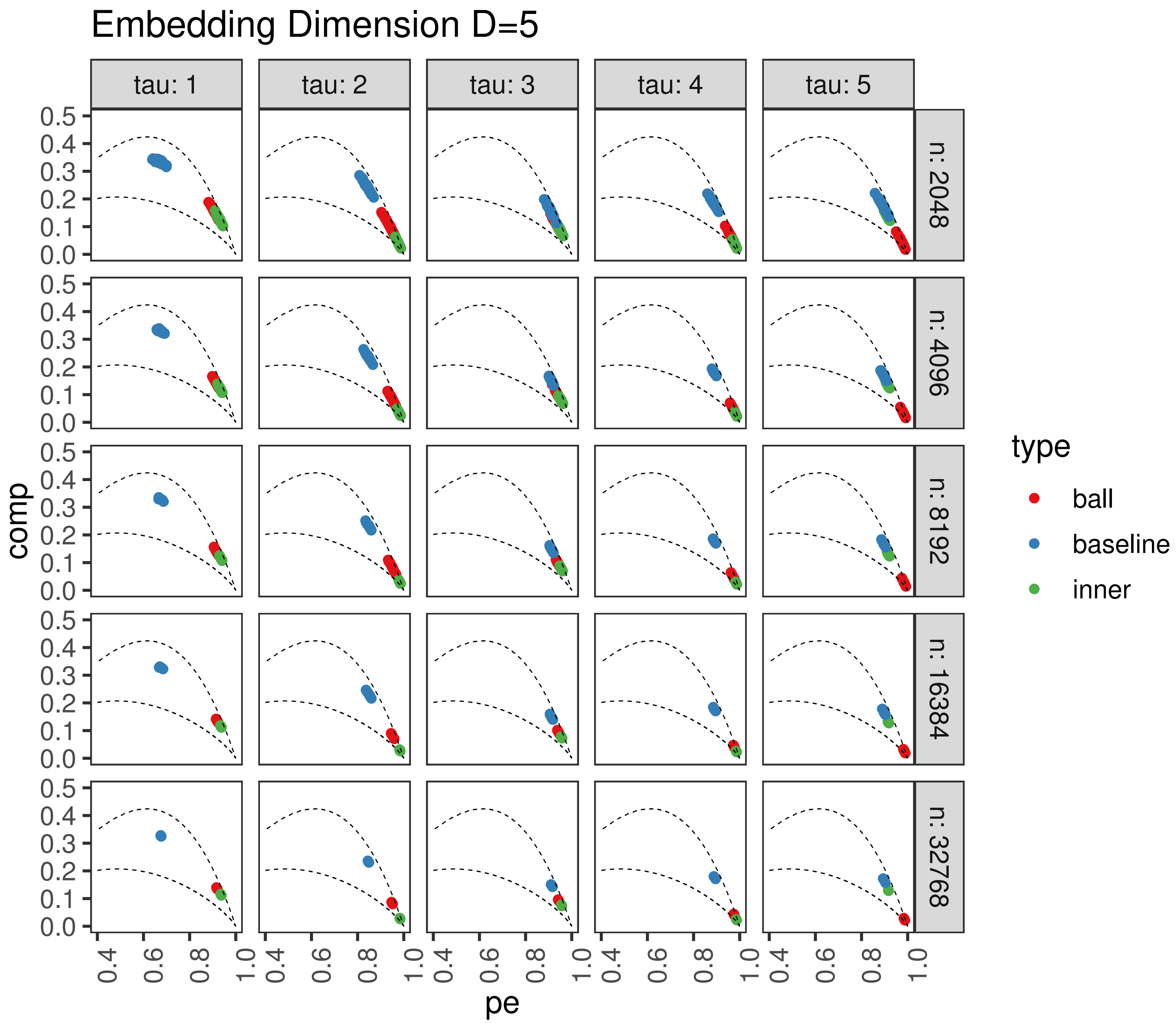

Figure S18: Fan end bearing, Load=0 hp, Fault Diameter=0.007 inches, Rotational Speed = 1797 rpm. The dashed lines represent the lower and upper limit curves. $D = 5$

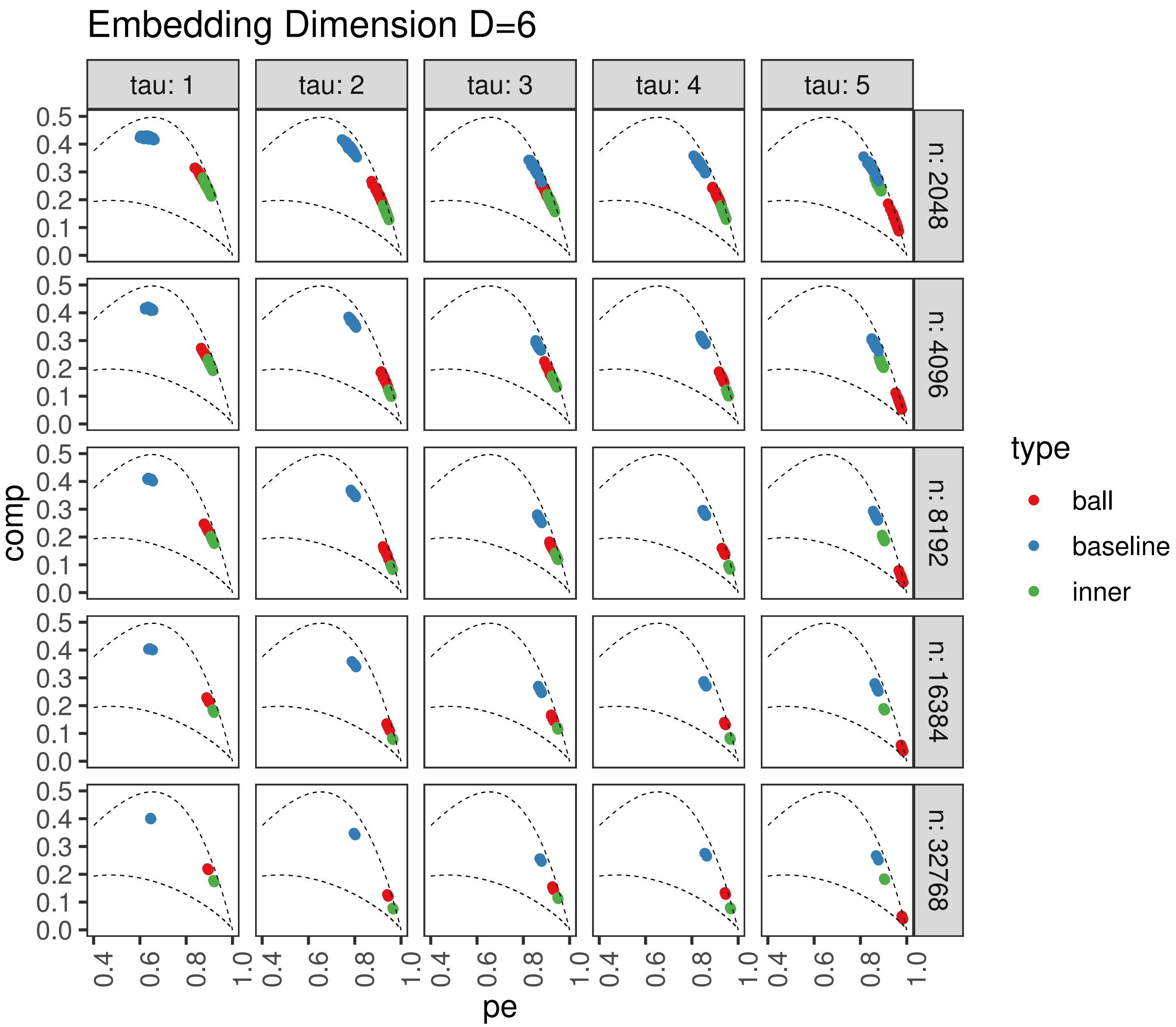

Figure S19: Fan end bearing, Load=0 hp, Fault Diameter=0.007 inches, Rotational Speed = 1797 rpm. The dashed lines represent the lower and upper limit curves. $D = 6$

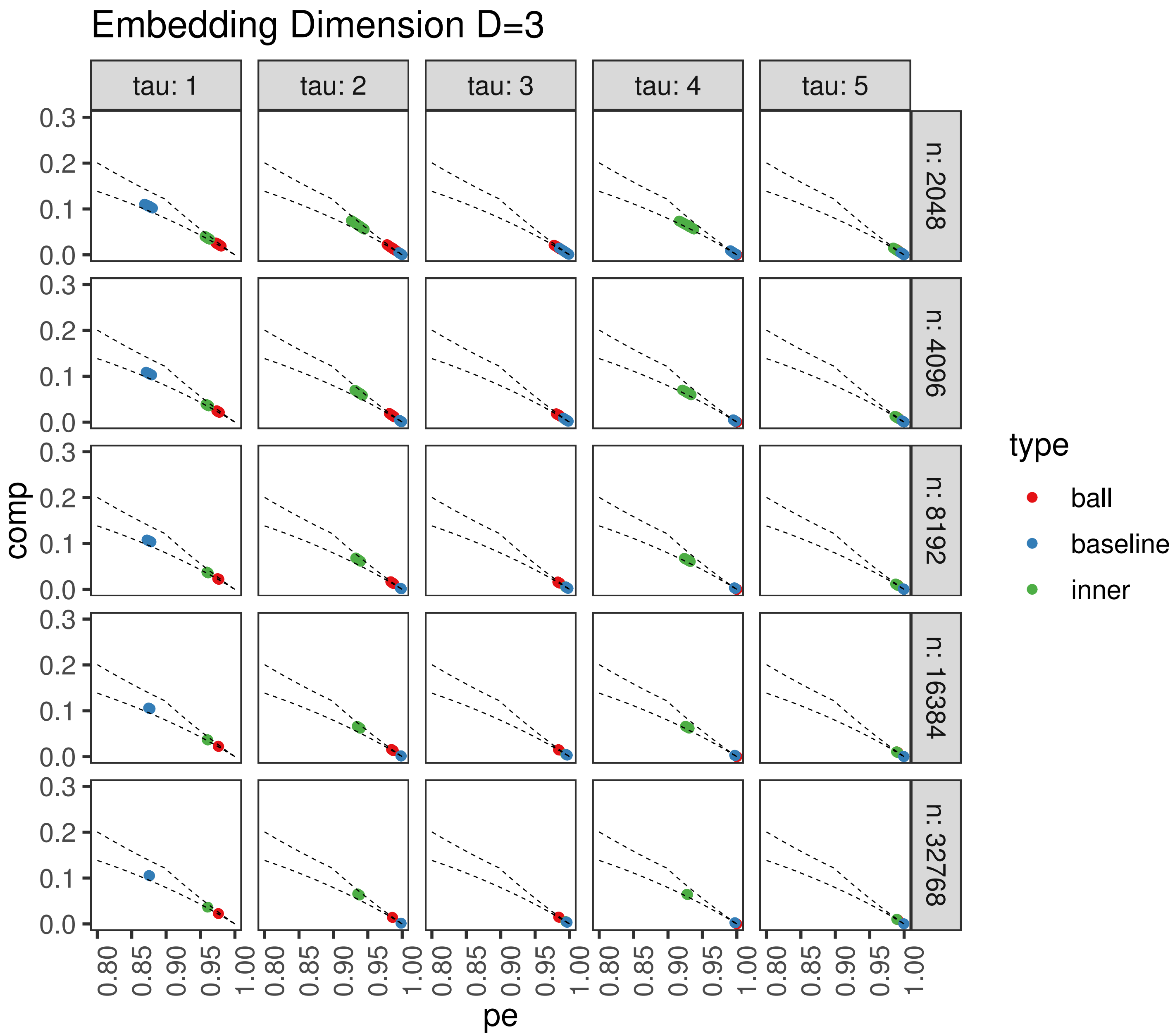

Figure S20: Drive end bearing, Load=3 hp, Fault Diameter=0.021 inches, Rotational Speed = 1730 rpm. The dashed lines represent the lower and upper limit curves. $D = 3$

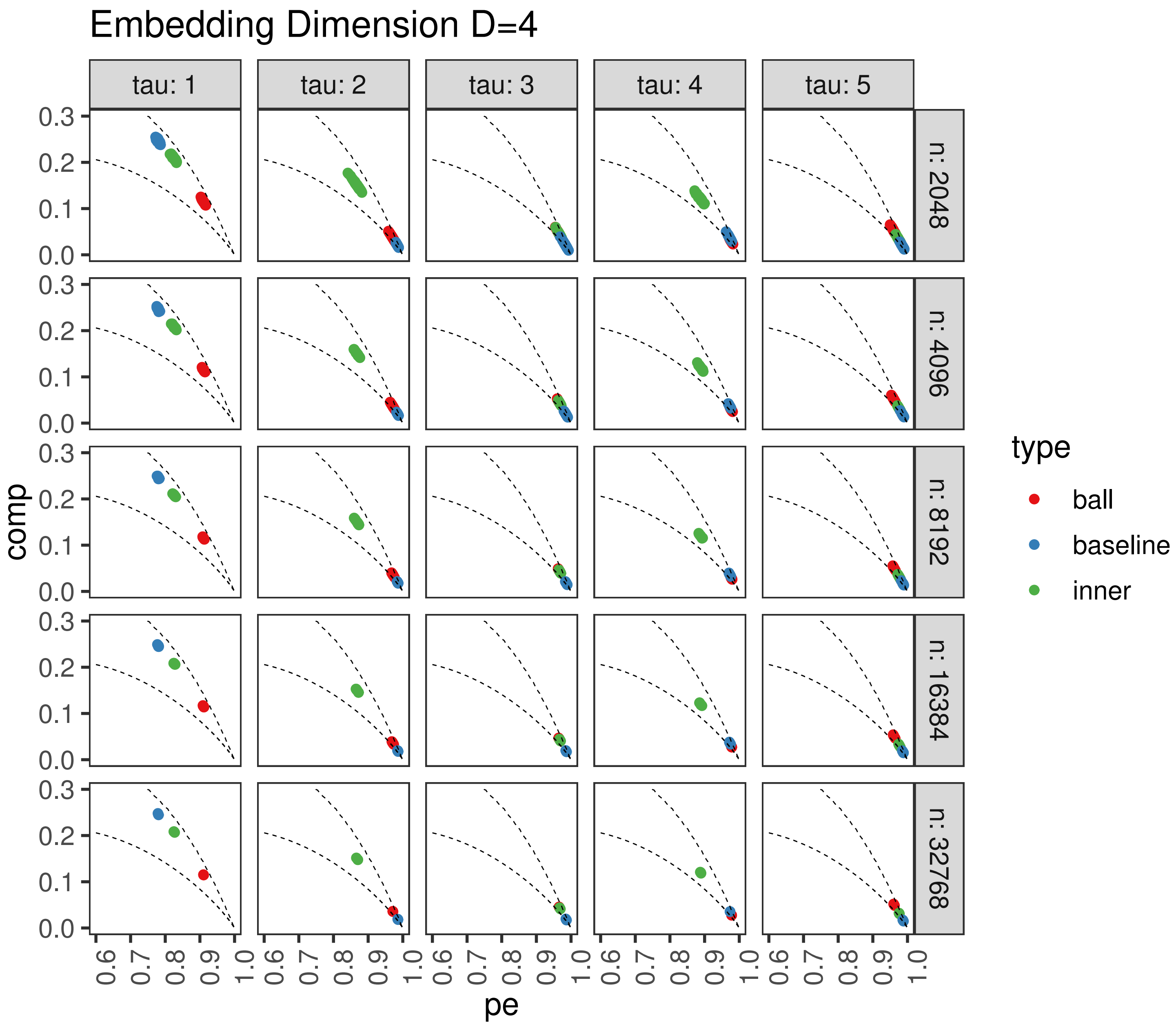

Figure S21: Drive end bearing, Load=3 hp, Fault Diameter=0.021 inches, Rotational Speed = 1730 rpm. The dashed lines represent the lower and upper limit curves. $D = 4$

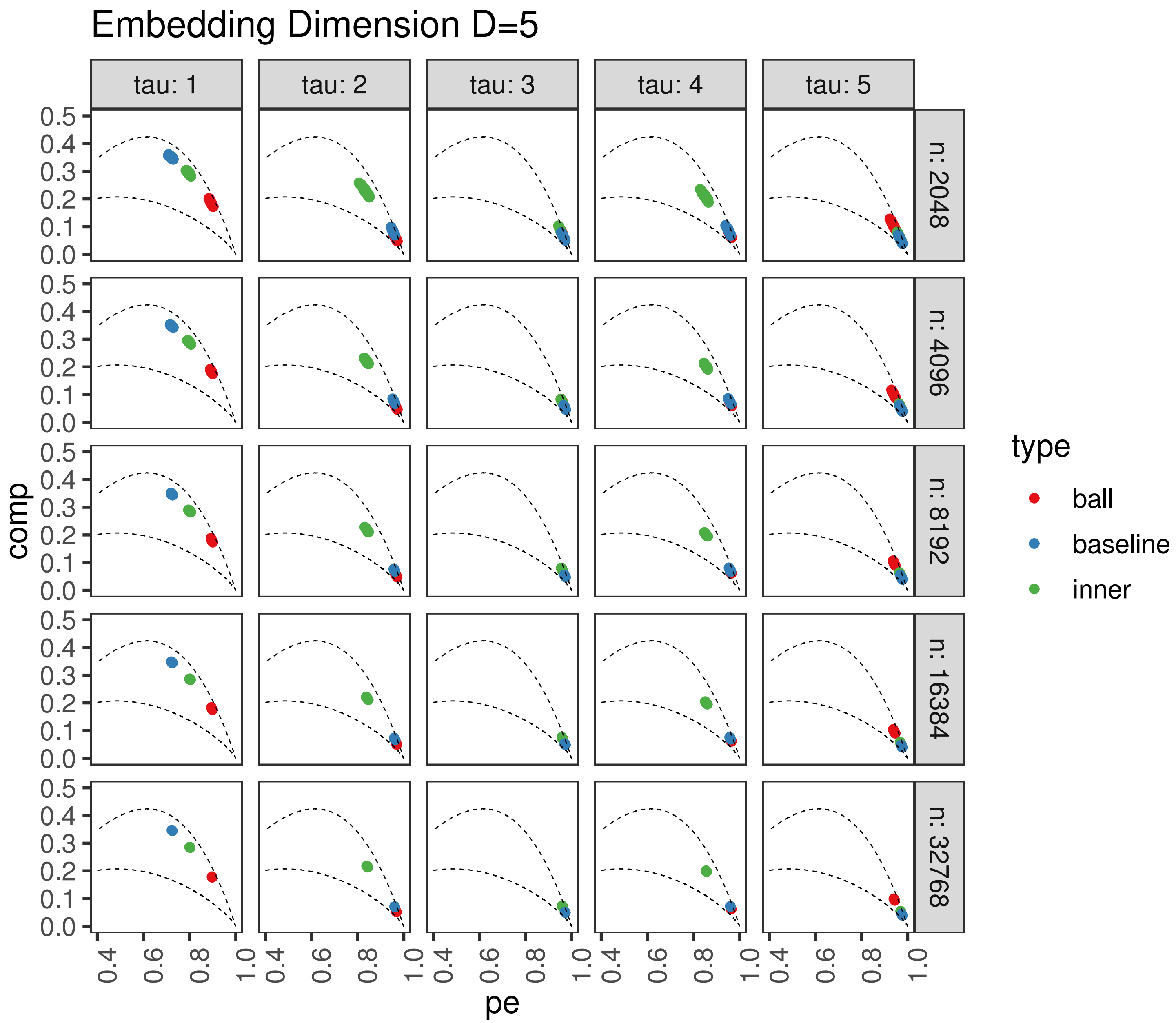

Figure S22: Drive end bearing, Load=3 hp, Fault Diameter=0.021 inches, Rotational Speed = 1730 rpm. The dashed lines represent the lower and upper limit curves. $D = 5$

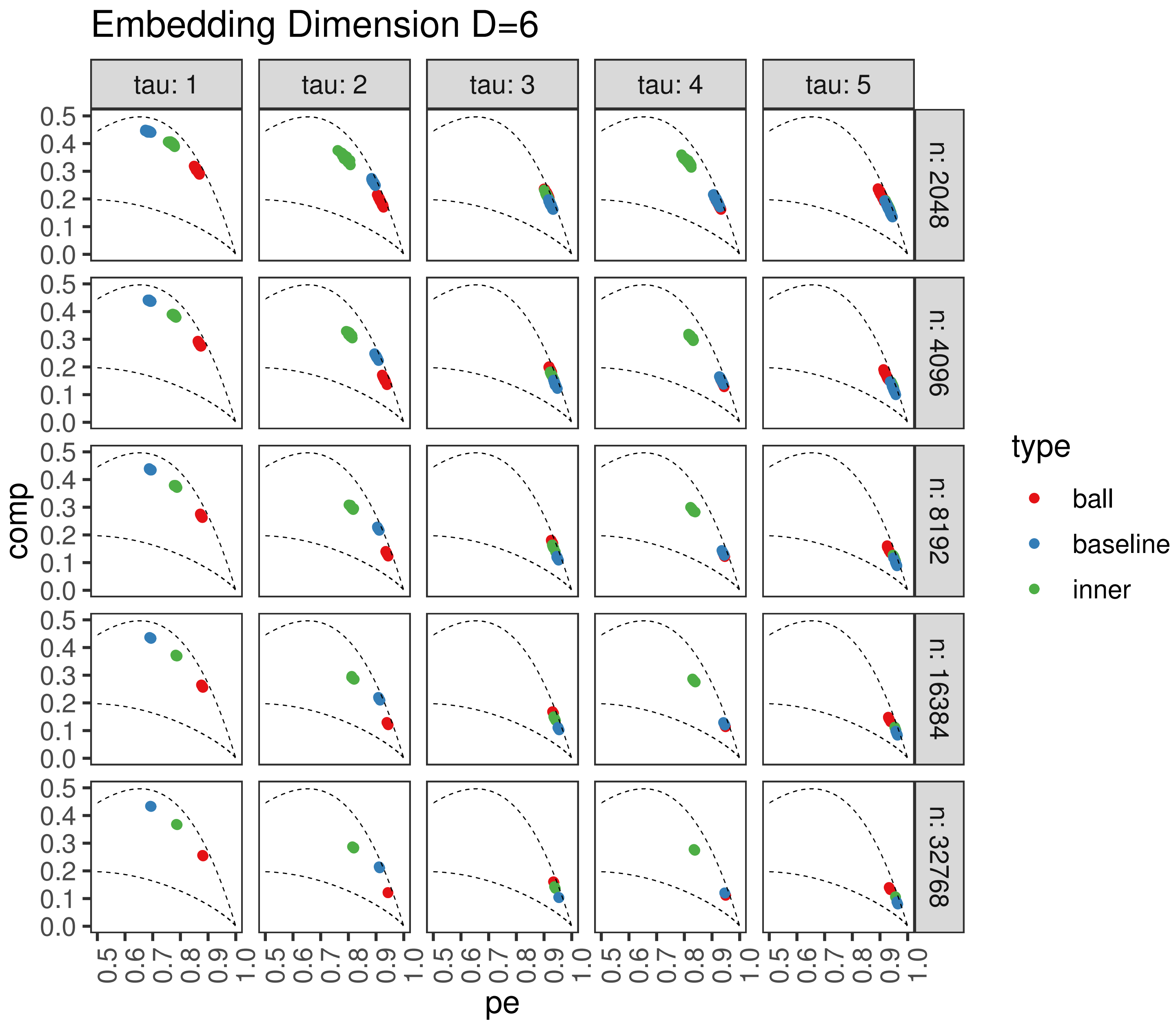

Figure S23: Drive end bearing, Load=3 hp, Fault Diameter=0.021 inches, Rotational Speed = 1730 rpm. The dashed lines represent the lower and upper limit curves. $D = 6$

3.2 SVM Classifier Performance for Selected Drive-end and Fan-end Bearing

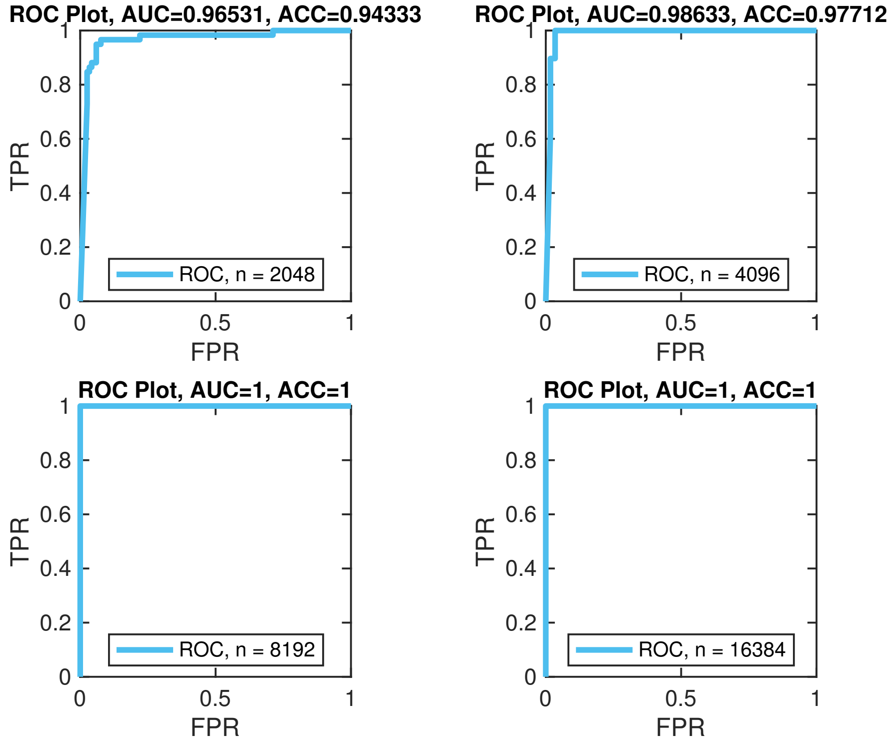

Figure S24: SVM results for fan end bearing, Load=0 hp, Fault Diameter=0.007 inches, Rotational Speed = 1797 rpm, $D = 6$, and $\tau = 1$

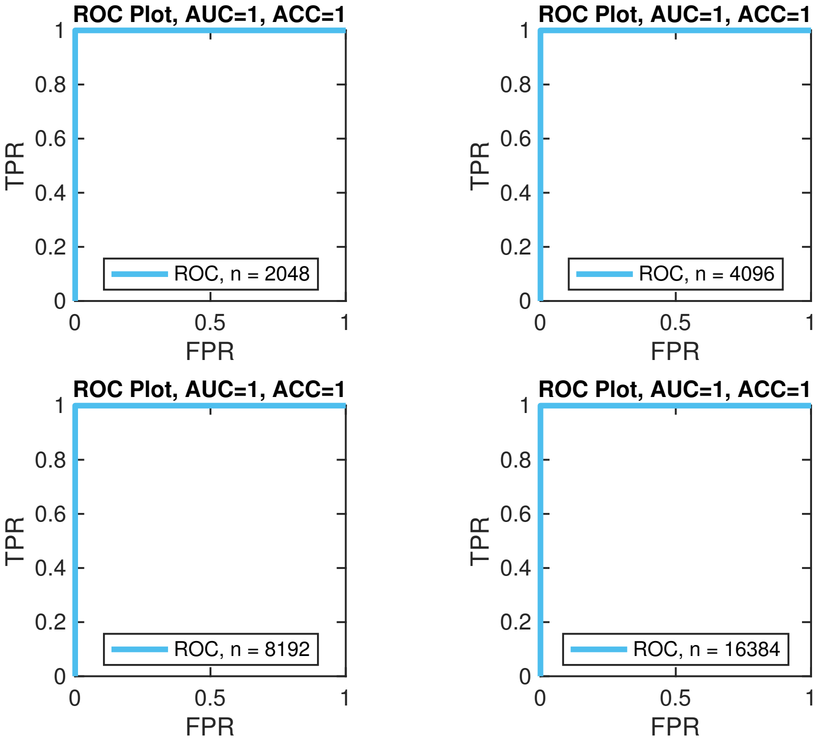

Figure S25: SVM results for drive end bearing, Load=3 hp, Fault Diameter=0.021 inches, Rotational Speed = 1730 rpm, $D = 6$, and $\tau = 1$

3.3 Classifier Accuracy for all Operating Conditions

We have included the results of the sensitivity analysis and the SVM classification for two cases. We observe the same pattern for other operating conditions where the classifier performance improves with respect to increase in the signal length. The plots for all conditions are not shown to conserve space. The SVM results for fan-end and drive-end bearings under all operating conditions, and parameters, $D = 6$ and $\tau = 1$ are presented in Table S3 and S4 respectively.

4.0 CECP Plots for PHM Gear Experiments

The details of the experiment is given in the main text. From the dataset we consider the case titled helical 1 (has no known gear defects were found) as the *baseline* case. We consider helical 2 (has a chipped tooth in helical gear with 24 teeth) as a *chipped tooth* gear category and helical 5 (has a broken tooth in helical gear with 24 teeth) as a *broken tooth* gear category. In all the three cases, we use the vibration signals recorded from accelerometer 2 (placed on the output side). The signals were recorded under two different load conditions (labeled as Low and High) and five different rotational speeds (i.e., 30 rps (1800 rpm), 35 rps (2100 rpm), 40 rps (2400 rpm), 45 rps (2700 rpm), and 50 rps (3000 rpm)). For each of these settings, two signals were recorded for four seconds each. Thus for one fault signal, 533,312 data points were generated for eight-second recording.

In the sections below we show plots of the sensitivity analysis for operating conditions speed 1800 rpm and 3000 rpm under low and high loads. The plots showing the performance of the SVM classifier for the above mentioned cases are also included.

Table S3: SVM classification accuracy results for fan-end bearing. n represents the signal length. $D = 6$ and $\tau = 1$

**Fault Diameter (mm)**	**Motor Load (kW)**	**Approx. Motor Speed (rps)**	$n = 2048$	$n = 4096$	$n = 8192$	$n = 16384$
0.1778 mm (0.007”)	0	29.95 rps (1797 rpm)	94.33	97.71	100	100
	0.7355 kW (1 hp)	29.53 rps (1772 rpm)	70.10	72.41	85.27	91.00
	1.471 kW (2 hp)	29.16 rps (1750 rpm)	98.89	100	100	100
	2.206 kW (3 hp)	28.83 rps (1730 rpm)	98.85	100	100	100
0.3556 mm (0.014”)	0	29.95 rps (1797 rpm)	97.73	98.82	100	100
	0.7355 kW (1 hp)	29.53 rps (1772 rpm)	93.79	94.24	95.27	95
	1.471 kW (2 hp)	29.16 rps (1750 rpm)	100	100	100	100
	2.206 kW (3 hp)	28.83 rps (1730 rpm)	96.61	100	100	100
0.5334 mm (0.021”)	0	29.95 rps (1797 rpm)	97.17	98.82	100	100
	0.7355 kW (1 hp)	29.53 rps (1772 rpm)	89.85	92.94	97.5	96
	1.471 kW (2 hp)	29.16 rps (1750 rpm)	77.36	79.15	92.5	90
	2.206 kW (3 hp)	28.83 rps (1730 rpm)	96.55	100	100	100

Table S4: SVM classification accuracy results for drive-end bearing. n represents the signal length. $D = 6$ and $\tau = 1$

**Fault Diameter (mm)**	**Motor Load (kW)**	**Approx. Motor Speed (rps)**	$n = 2048$	$n = 4096$	$n = 8192$	$n = 16384$
0.1778 mm (0.007")	0	29.95 rps (1797 rpm)	100	100	100	100
	0.7355 kW (1 hp)	29.53 rps (1772 rpm)	100	100	100	100
	1.471 kW (2 hp)	29.16 rps (1750 rpm)	100	100	100	100
	2.206 kW (3 hp)	28.83 rps (1730 rpm)	100	100	100	100
0.3556 mm (0.014")	0	29.95 rps (1797 rpm)	100	100	100	100
	0.7355 kW (1 hp)	29.53 rps (1772 rpm)	100	100	100	100
	1.471 kW (2 hp)	29.16 rps (1750 rpm)	98.87	100	100	100
	2.206 kW (3 hp)	28.83 rps (1730 rpm)	98.88	97.64	97.5	100
0.5334 mm (0.021")	0	29.95 rps (1797 rpm)	100	100	100	100
	0.7355 kW (1 hp)	29.53 rps (1772 rpm)	100	100	100	100
	1.471 kW (2 hp)	29.16 rps (1750 rpm)	99.42	100	100	100
	2.206 kW (3 hp)	28.83 rps (1730 rpm)	100	100	100	100

4.1 Sensitivity Analysis

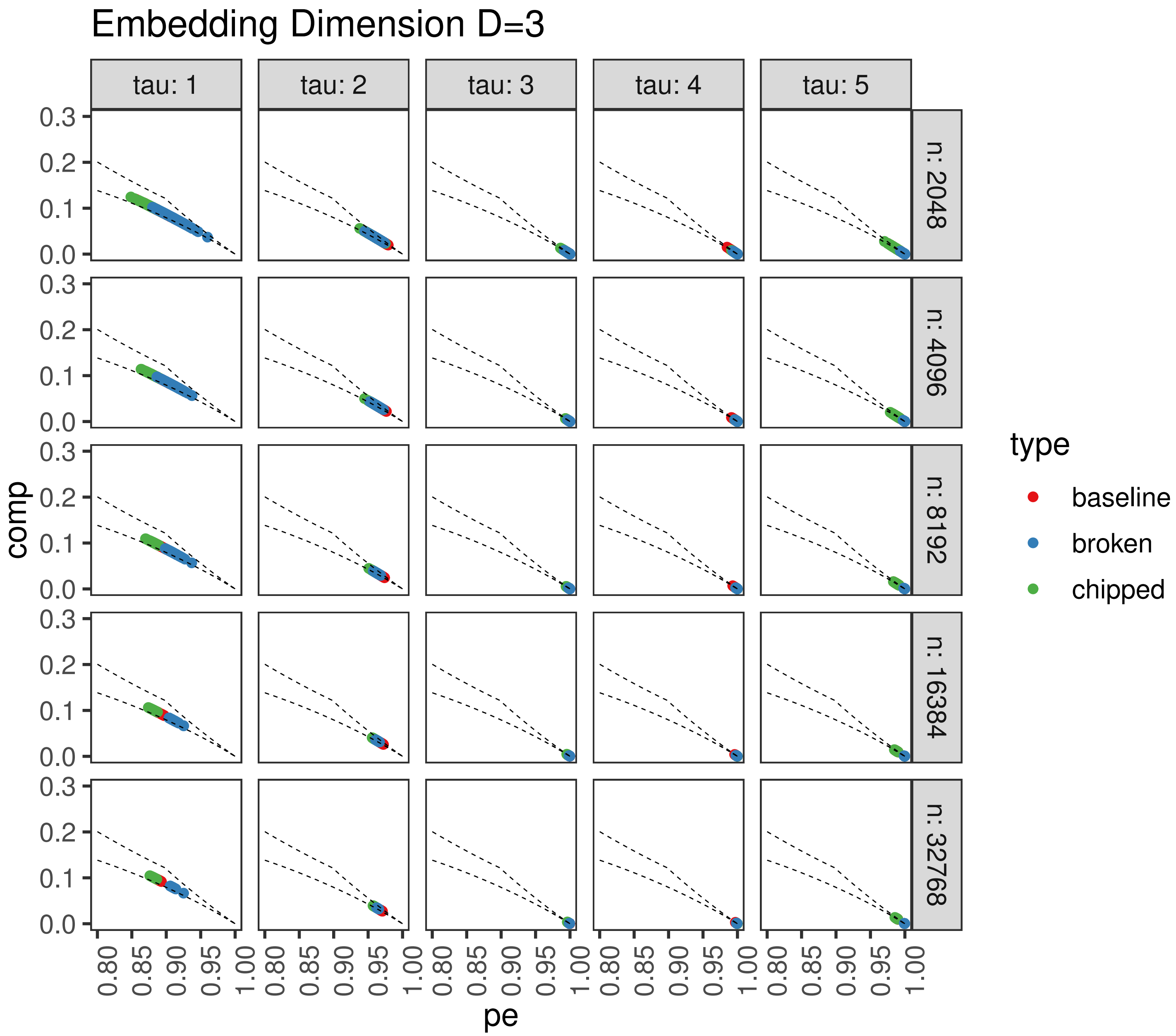

Figure S26: Load = high, Rotational speed = 1800 rpm. The dashed lines represent the lower and upper limit curves. $D = 3$

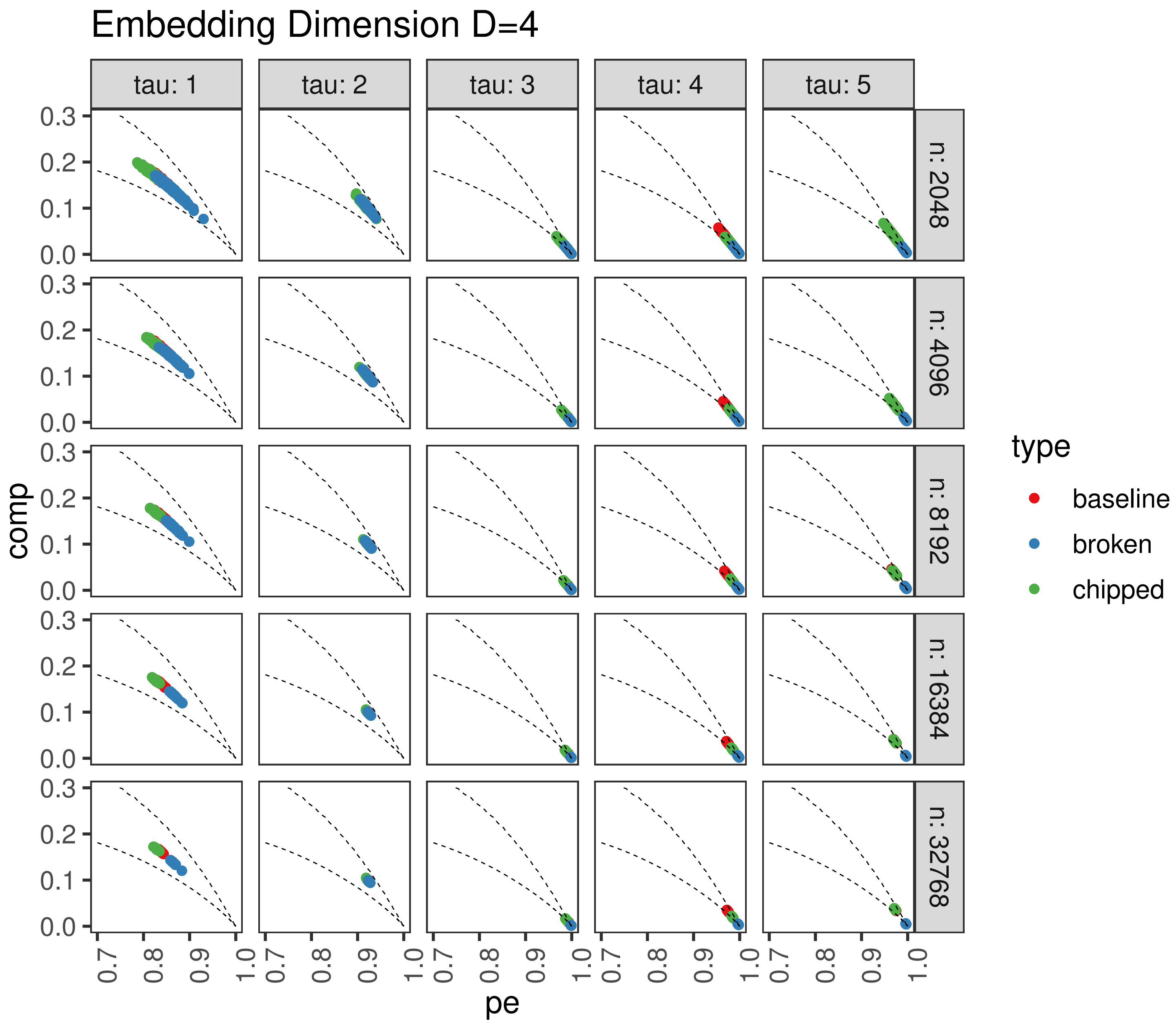

Figure S27: Load = high, Rotational speed = 1800 rpm. The dashed lines represent the lower and upper limit curves. $D = 4$

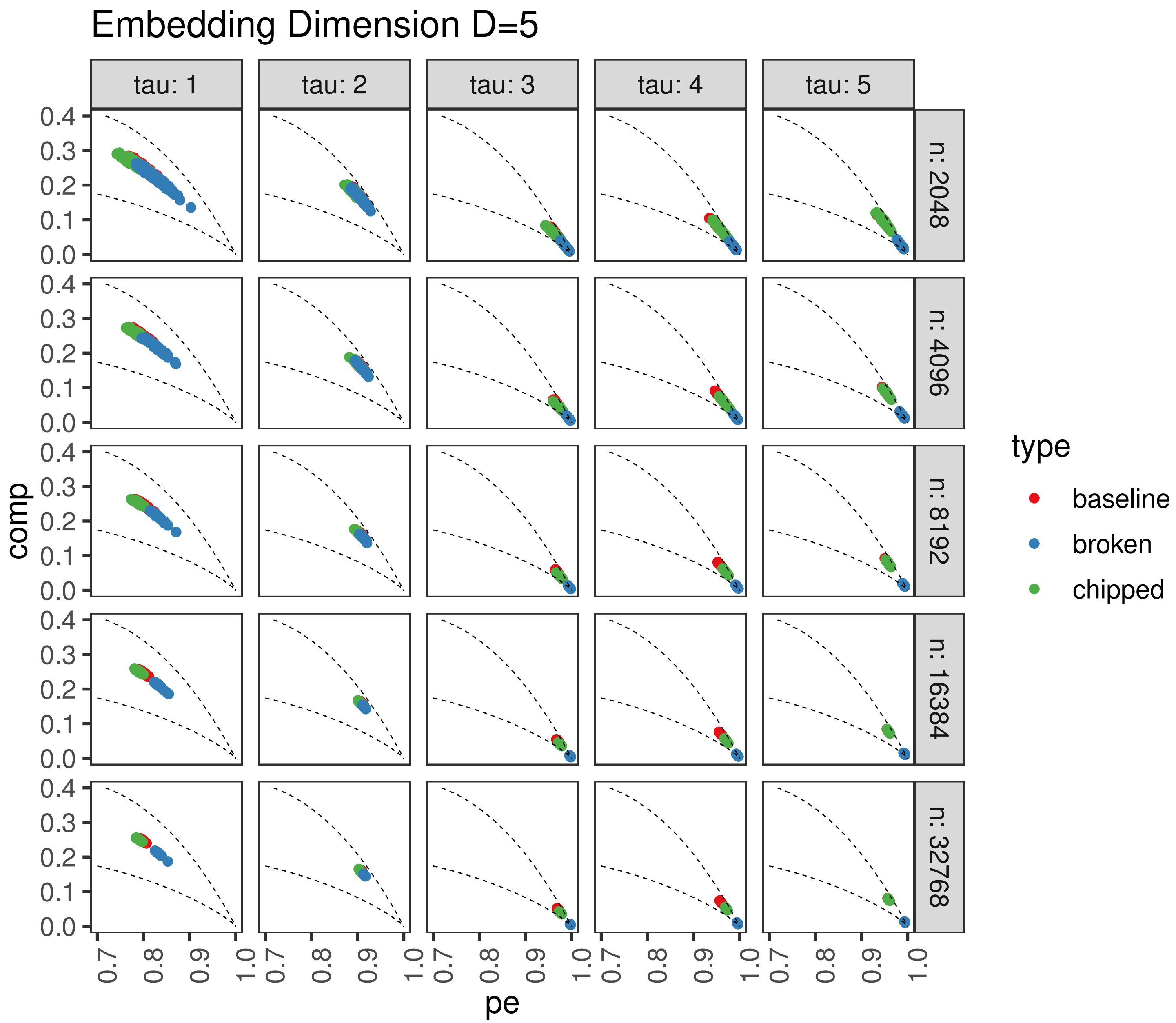

Figure S28: Load = high, Rotational speed = 1800 rpm. The dashed lines represent the lower and upper limit curves. $D = 5$

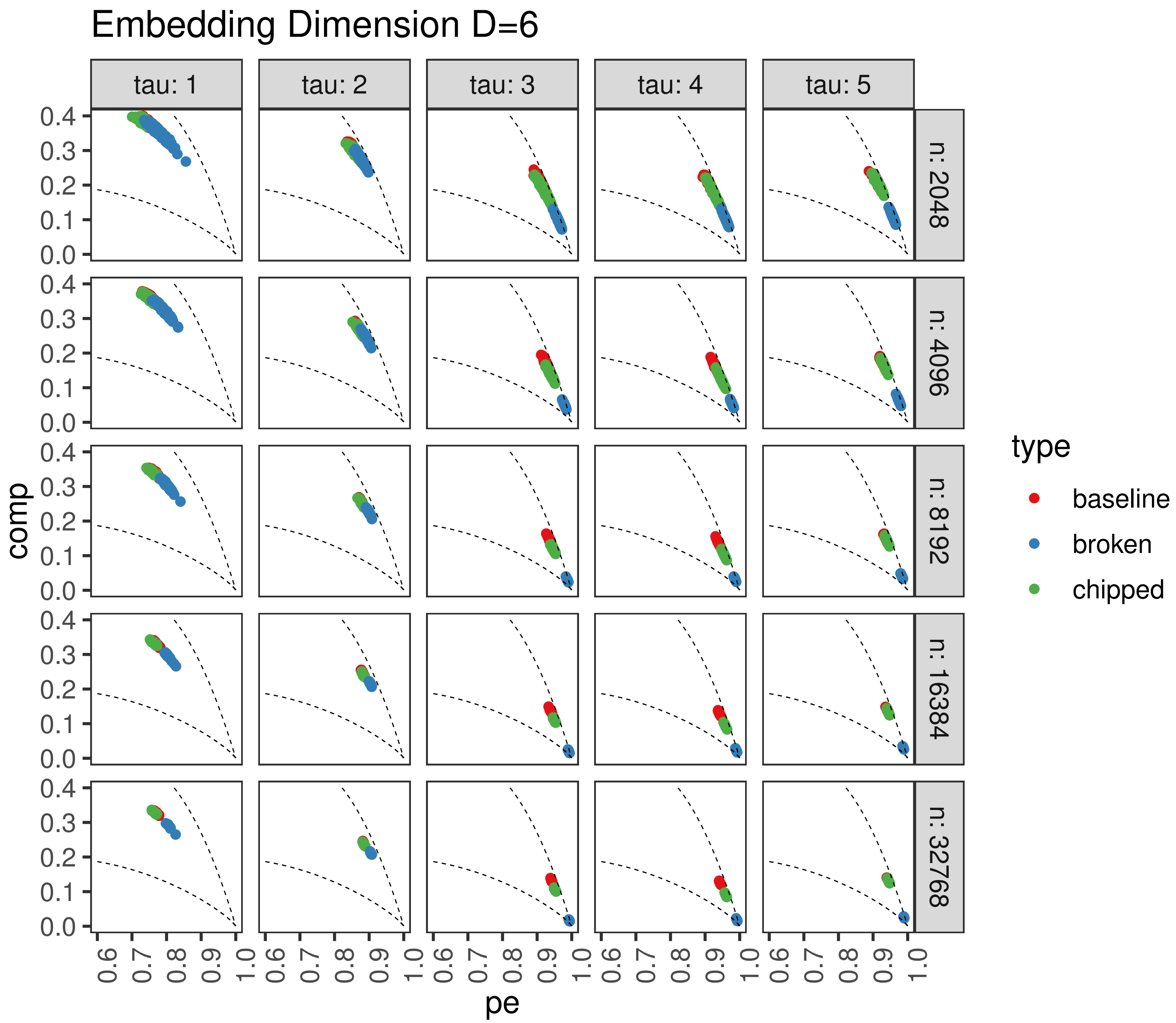

Figure S29: Load = high, Rotational speed = 1800 rpm. The dashed lines represent the lower and upper limit curves. $D = 6$

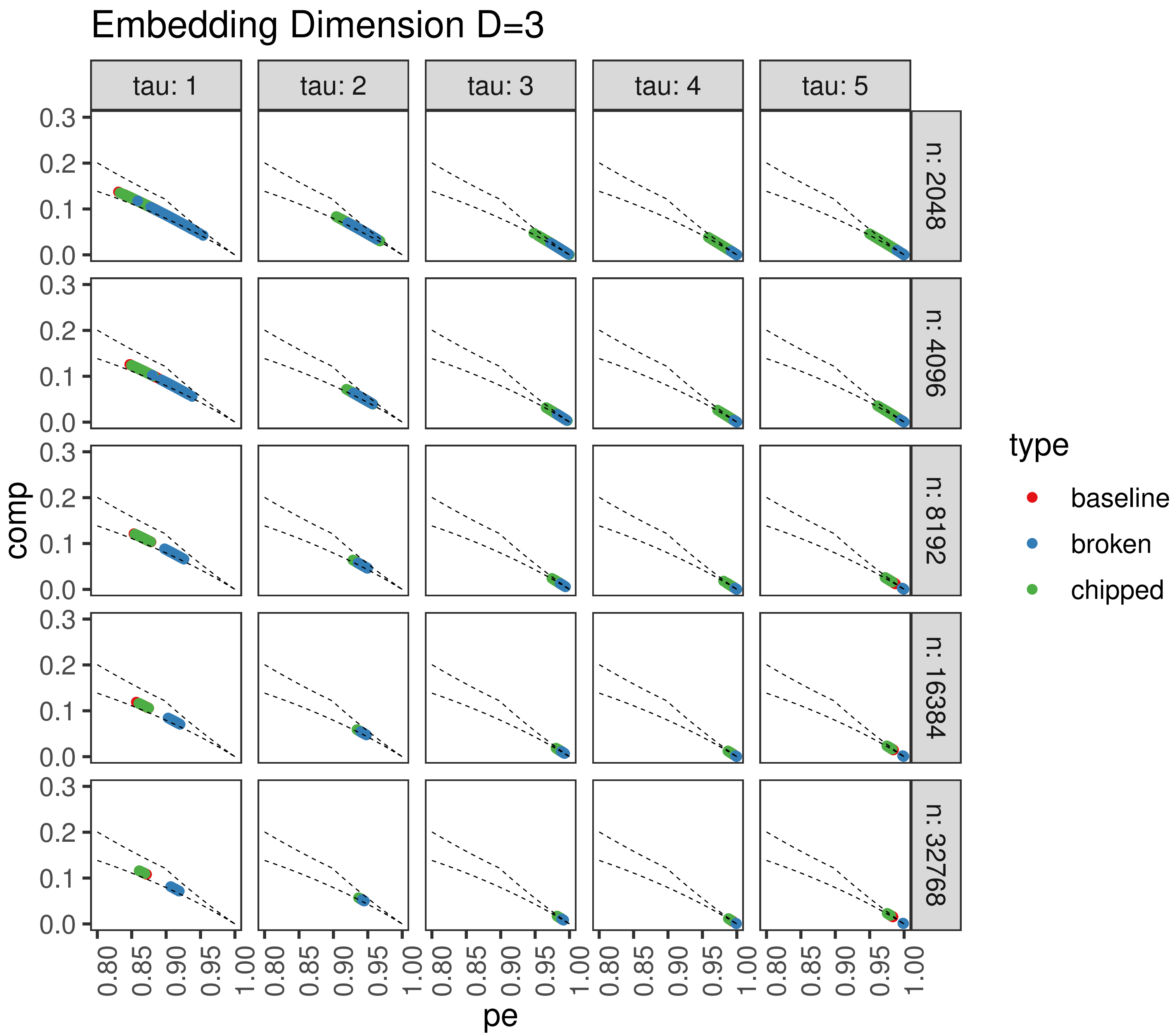

Figure S30: Load = low, Rotational speed = 1800 rpm. The dashed lines represent the lower and upper limit curves. $D = 3$

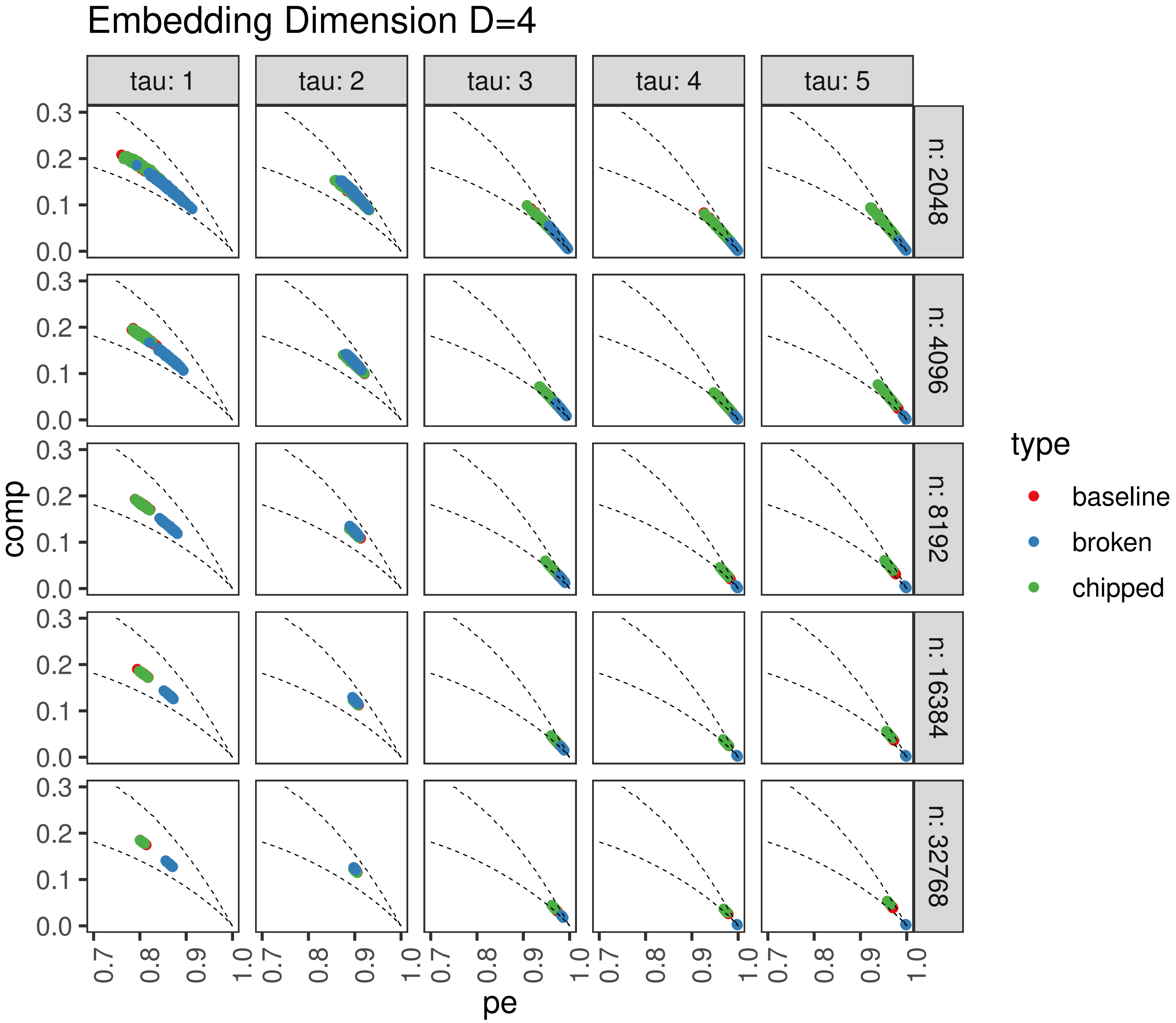

Figure S31: Load = low, Rotational speed = 1800 rpm. The dashed lines represent the lower and upper limit curves. $D = 4$

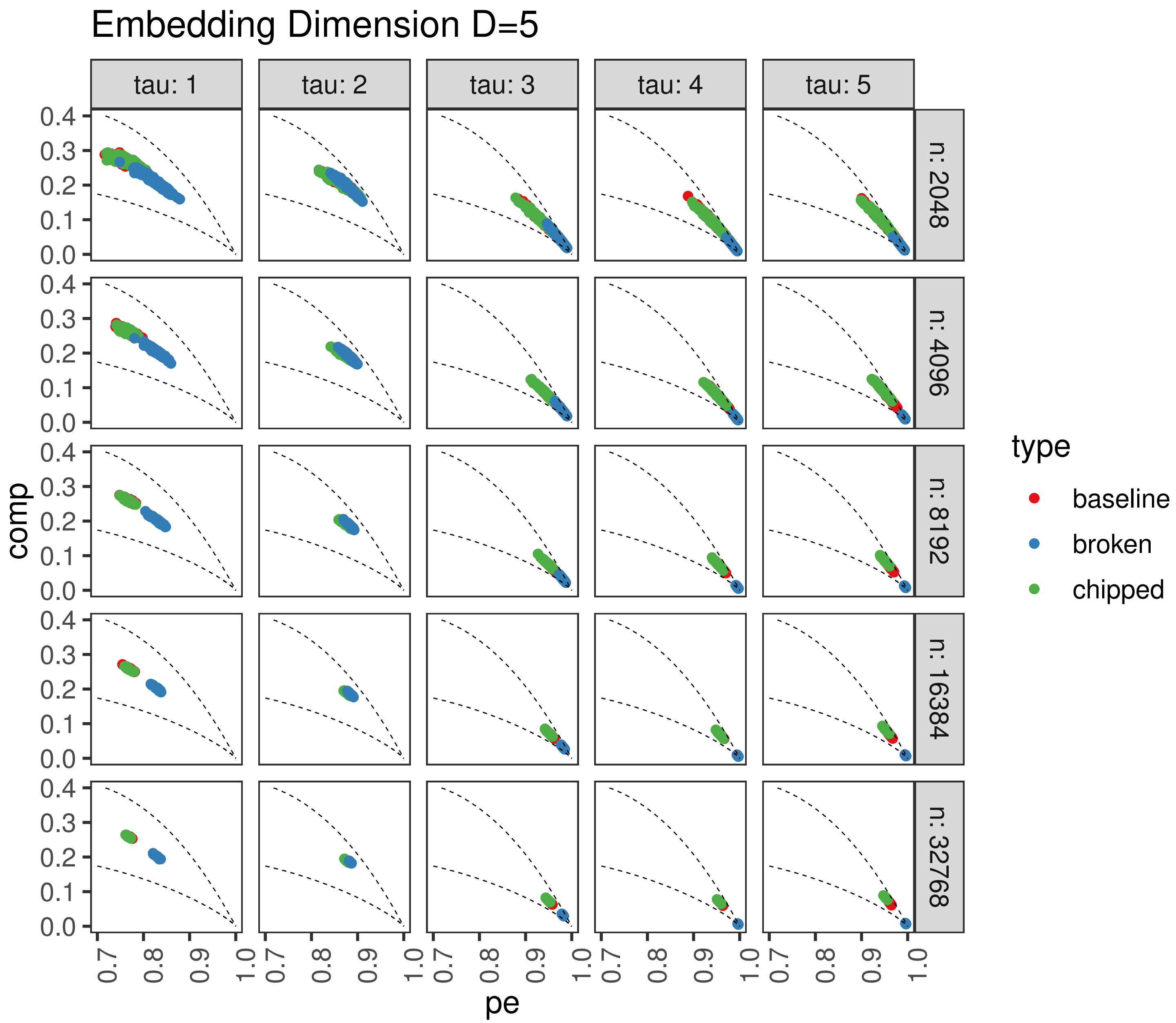

Figure S32: Load = low, Rotational speed = 1800 rpm. The dashed lines represent the lower and upper limit curves. $D = 5$

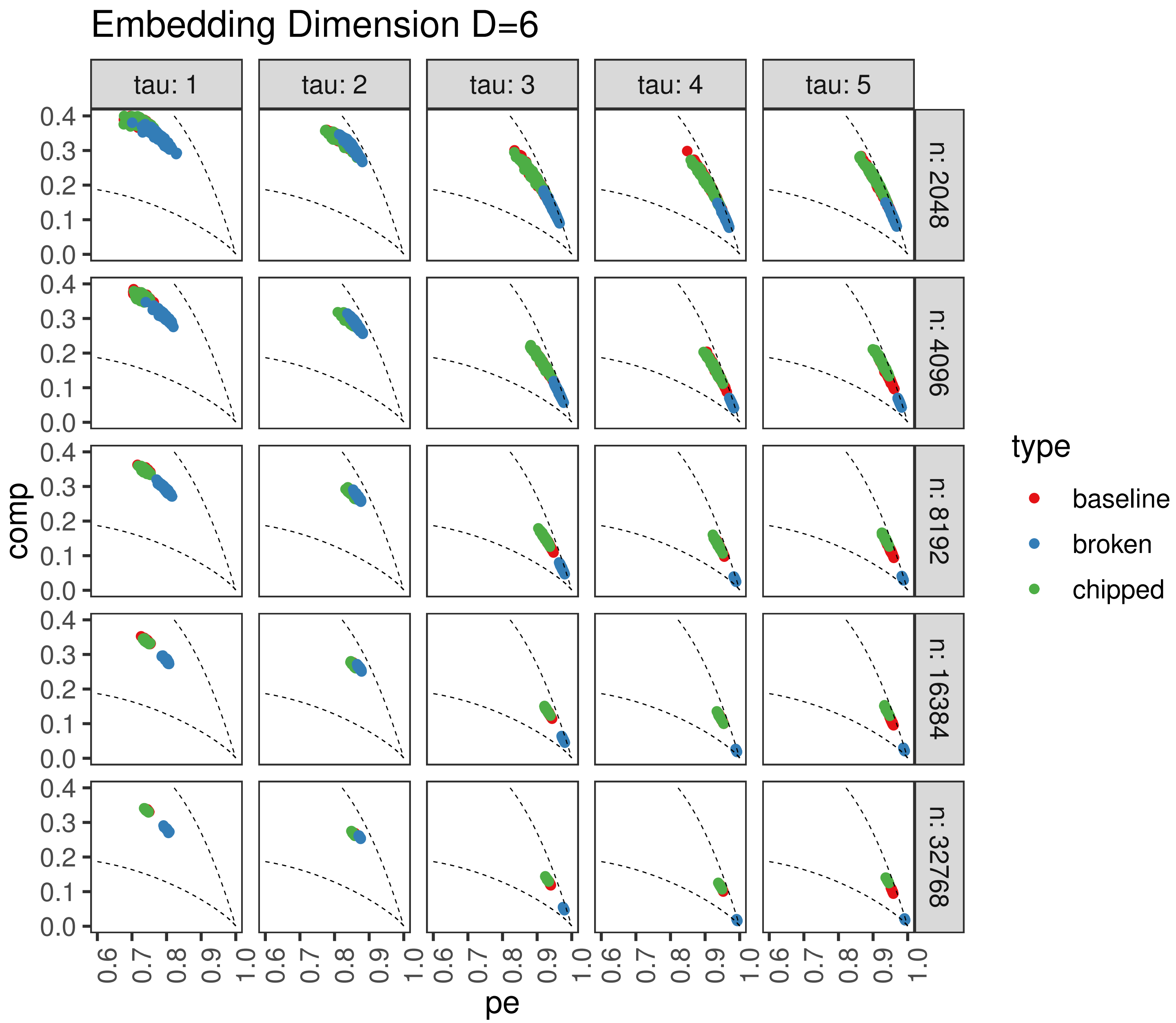

Figure S33: Load = low, Rotational speed = 1800 rpm. The dashed lines represent the lower and upper limit curves. $D = 6$

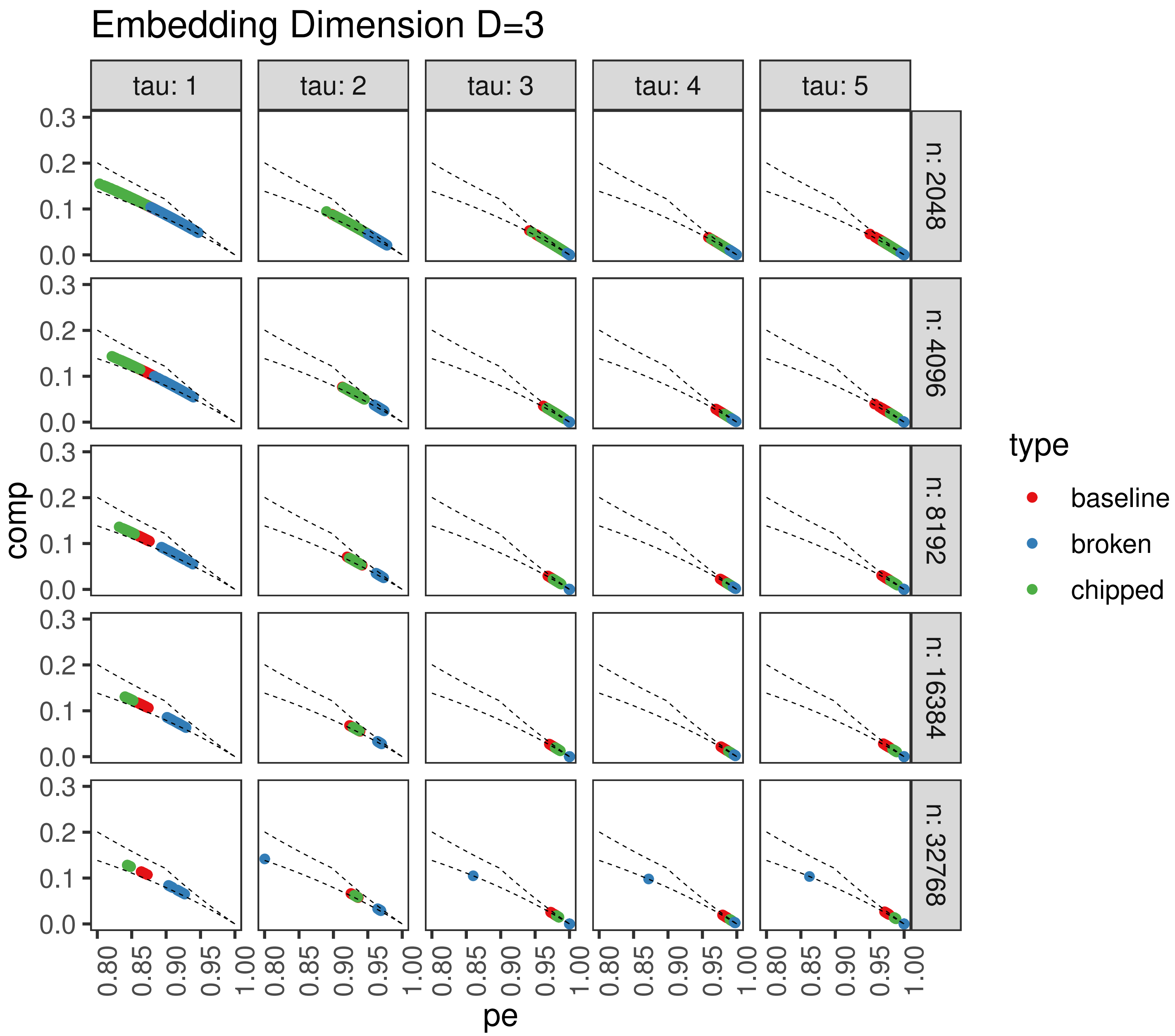

Figure S34: Load = high, Rotational speed = 3000 rpm. The dashed lines represent the lower and upper limit curves. $D = 3$

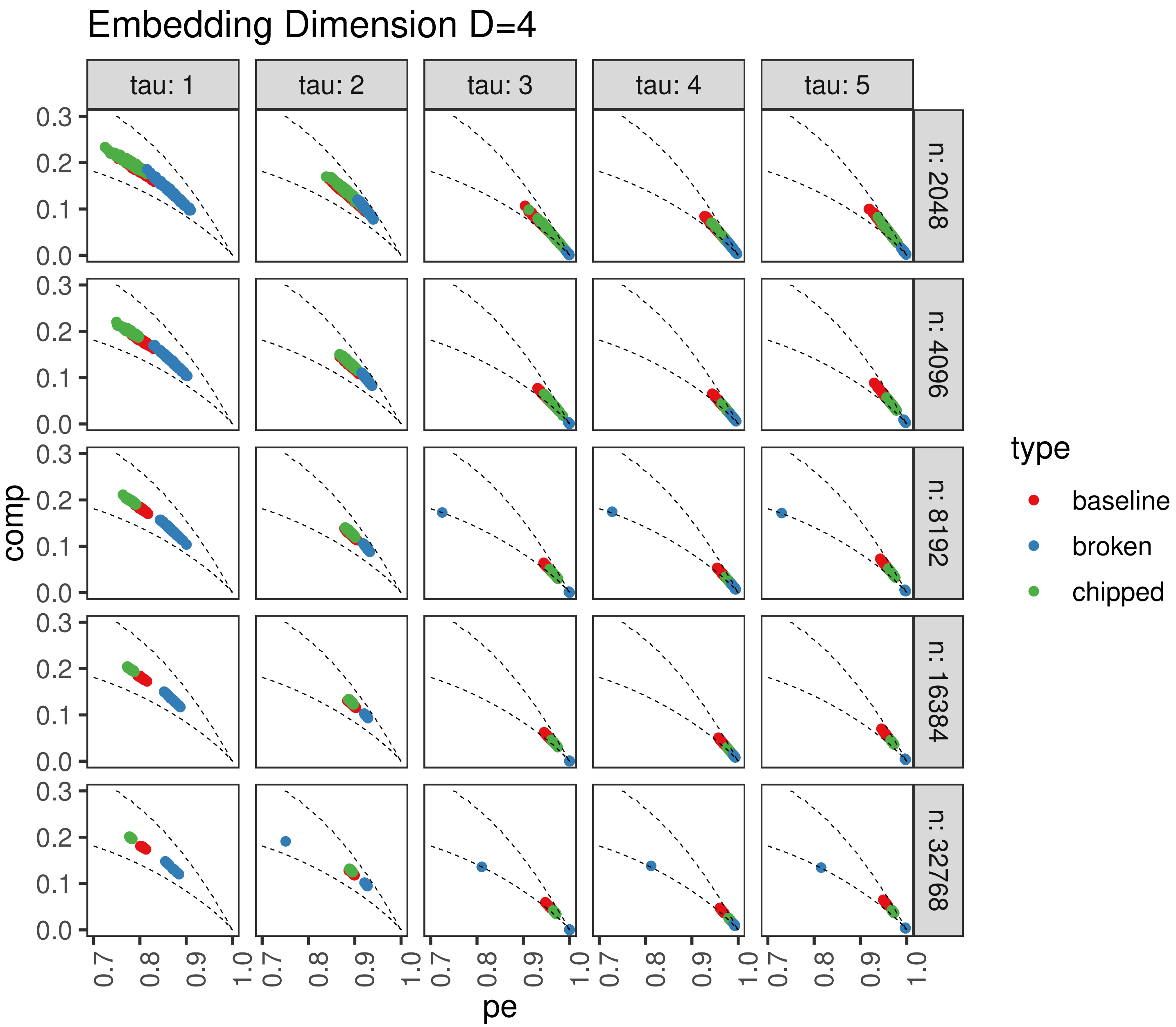

Figure S35: Load = high, Rotational speed = 3000 rpm. The dashed lines represent the lower and upper limit curves. $D = 4$

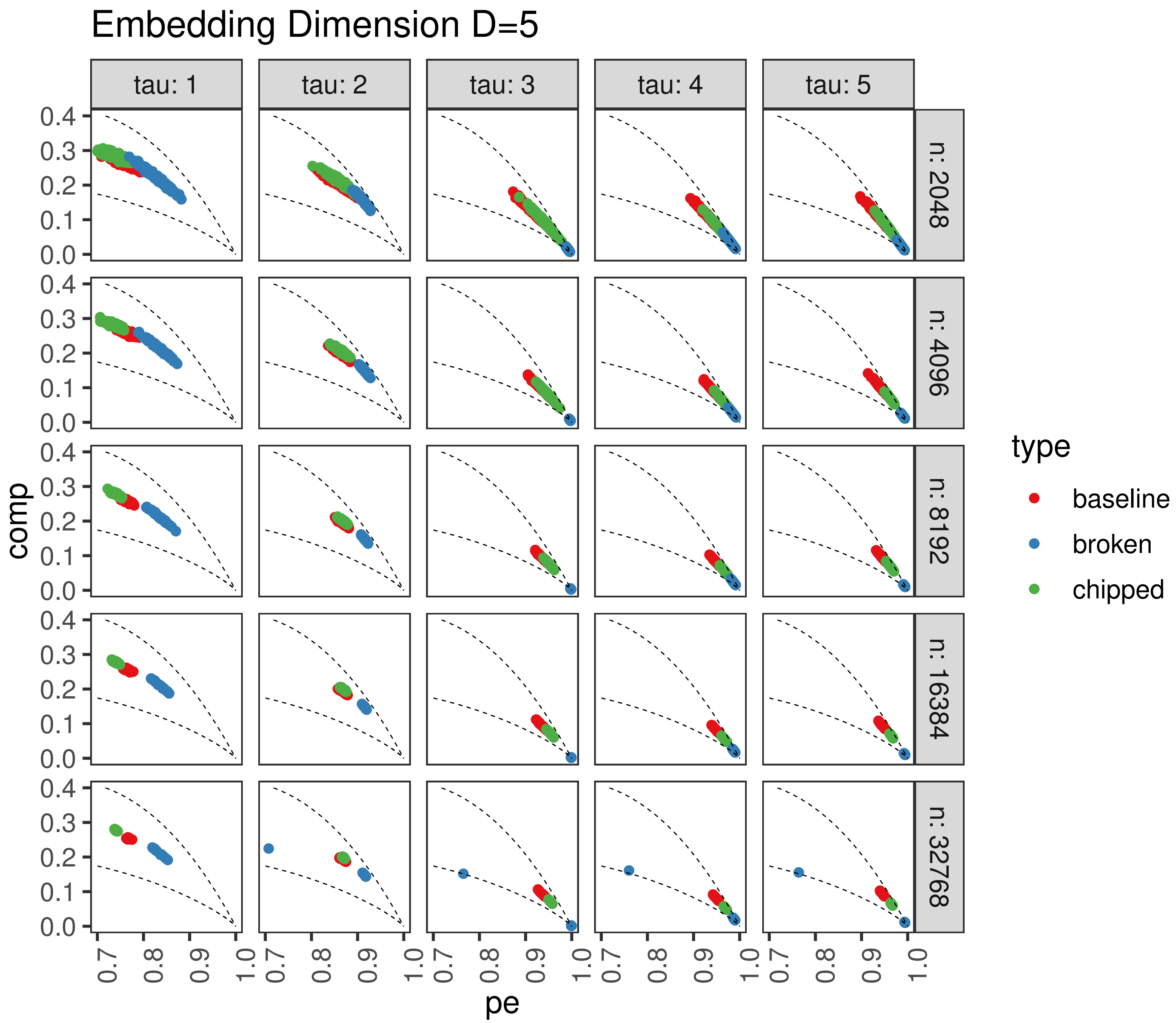

Figure S36: Load = high, Rotational speed = 3000 rpm. The dashed lines represent the lower and upper limit curves. $D = 5$

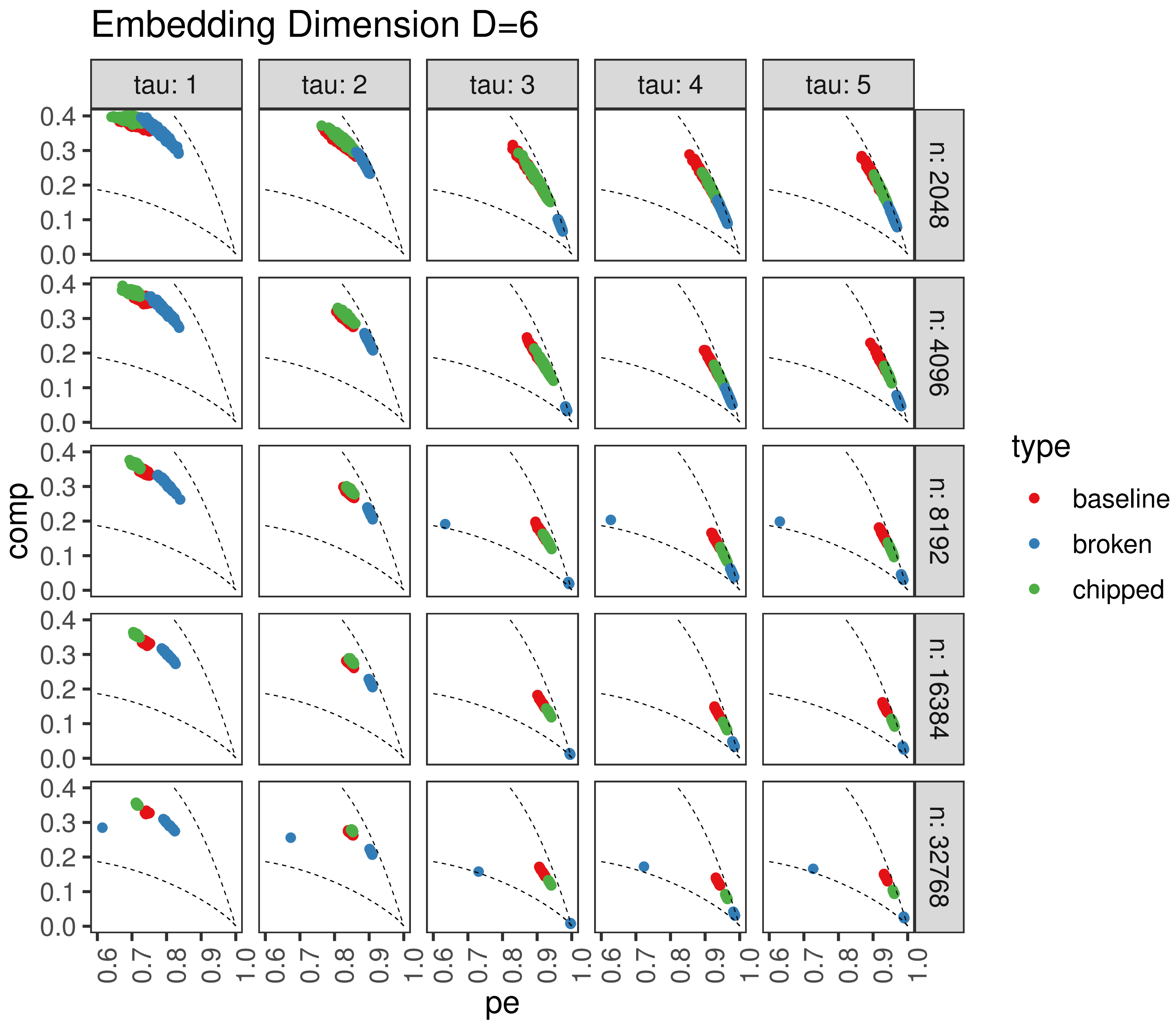

Figure S37: Load = high, Rotational speed = 3000 rpm. The dashed lines represent the lower and upper limit curves. $D = 6$

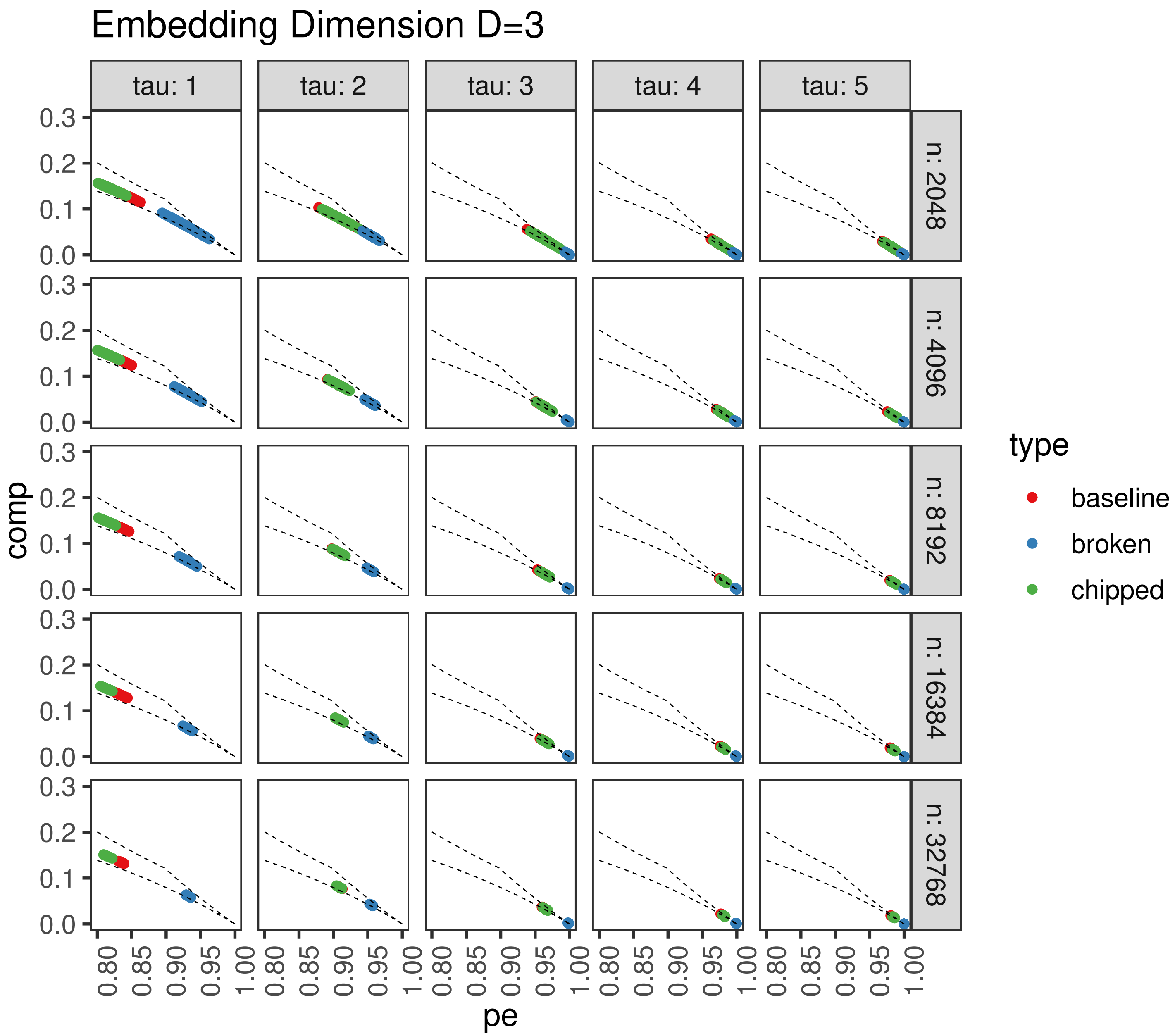

Figure S38: Load = low, Rotational speed = 3000 rpm. The dashed lines represent the lower and upper limit curves. $D = 3$

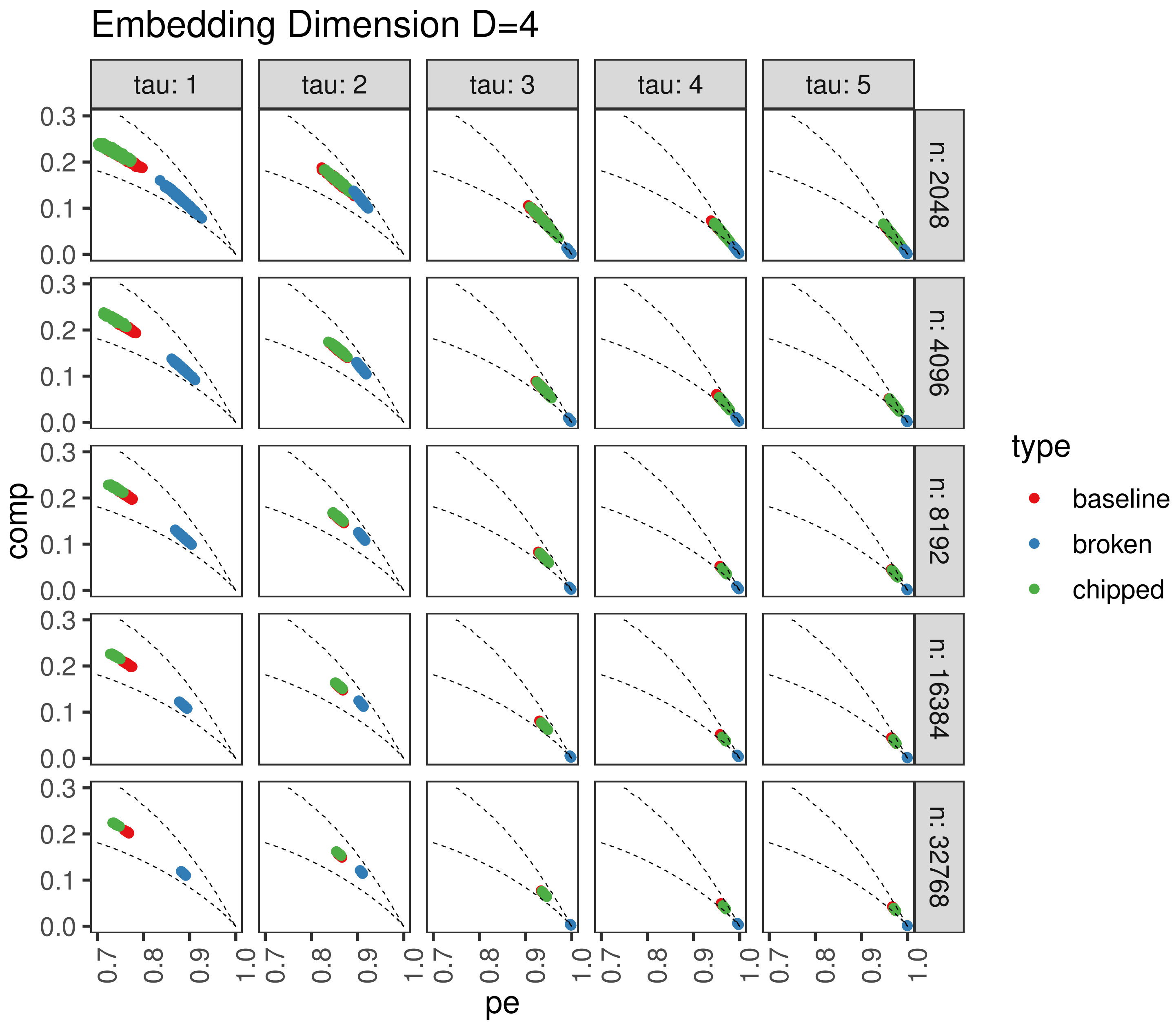

Figure S39: Load = low, Rotational speed = 3000 rpm. The dashed lines represent the lower and upper limit curves. $D = 4$

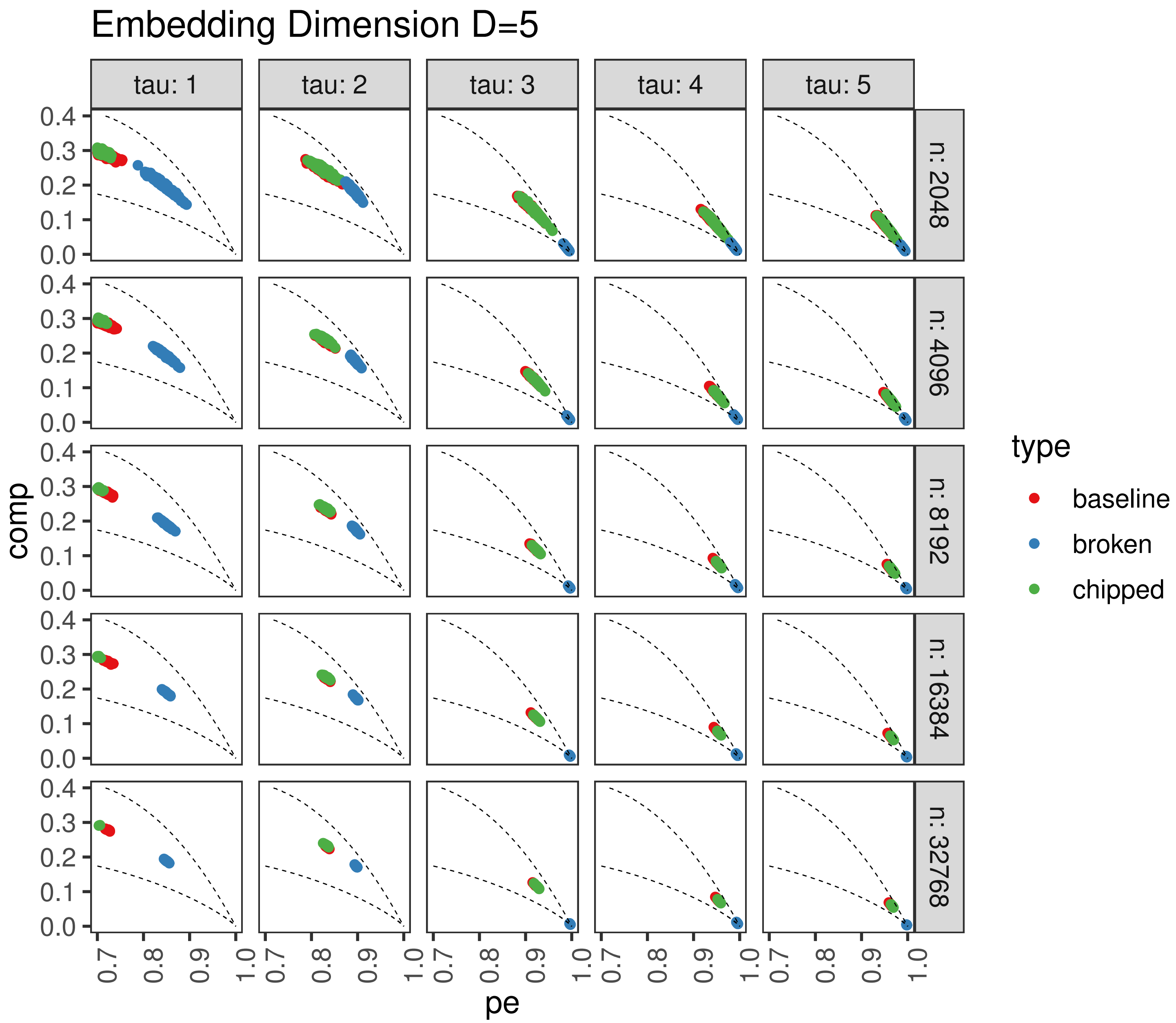

Figure S40: Load = low, Rotational speed = 3000 rpm. The dashed lines represent the lower and upper limit curves. $D = 5$

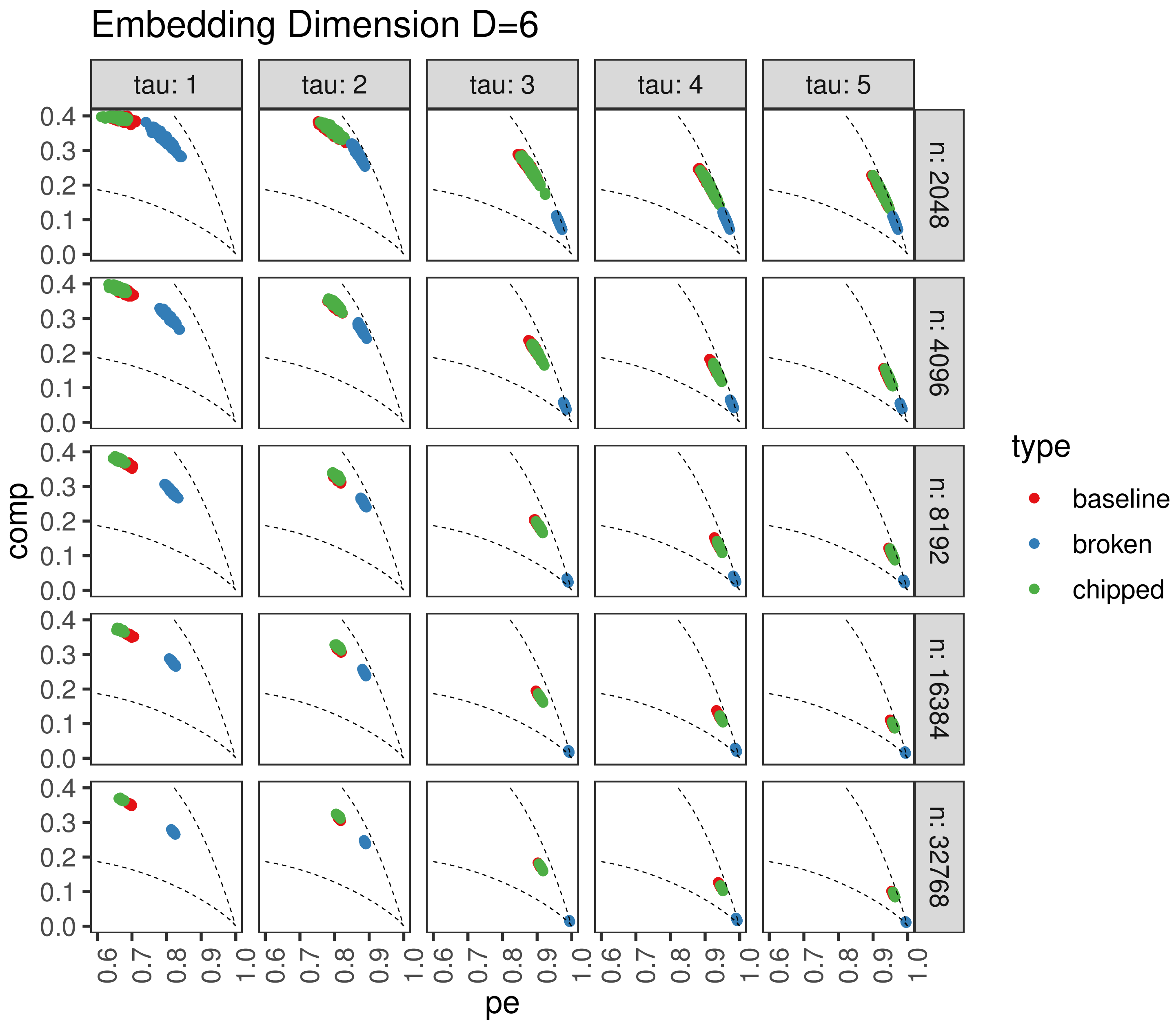

Figure S41: Load = low, Rotational speed = 3000 rpm. The dashed lines represent the lower and upper limit curves. $D = 6$

4.2 SVM Classifier Performance for 1800 rpm and 3000 rpm Case

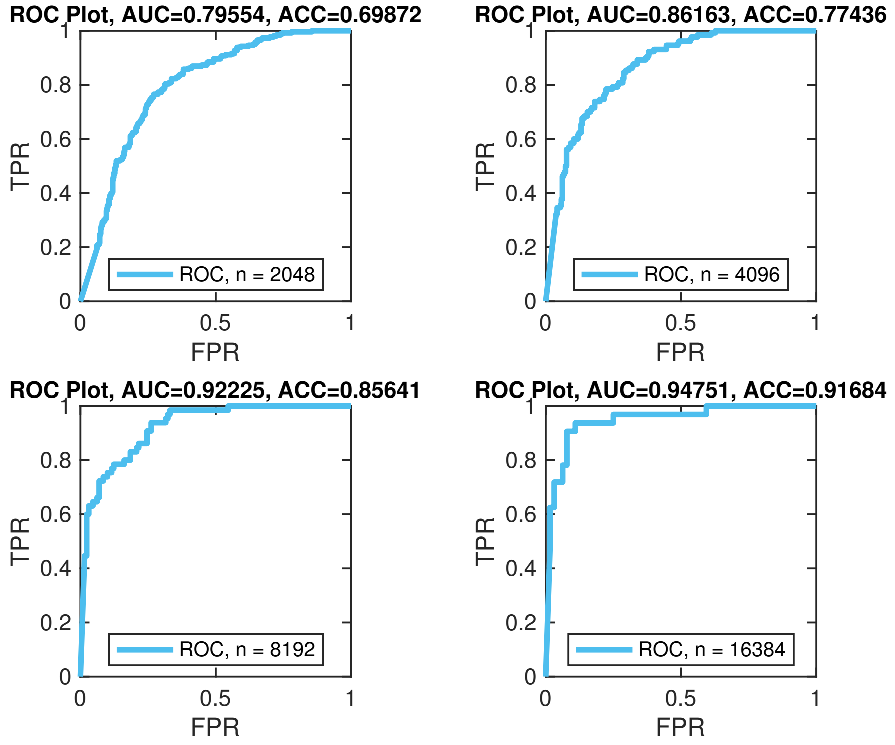

Figure S42: SVM results for Load= high, Rotational Speed = 1800 rpm, $D = 6$, and $\tau = 1$

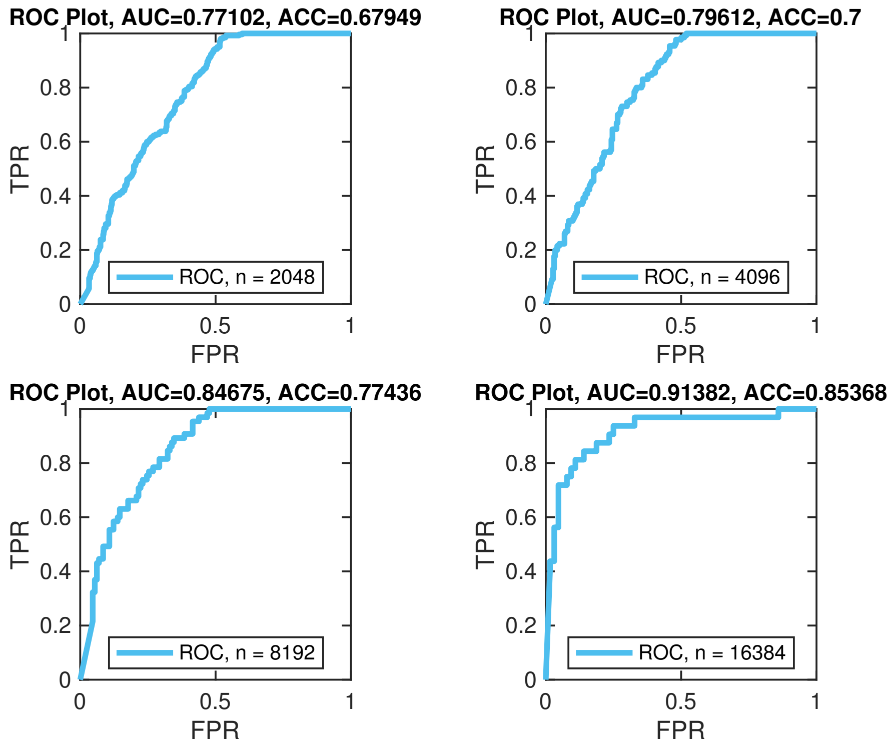

Figure S43: SVM results for Load= low, Rotational Speed = 1800 rpm, $D = 6$, and $\tau = 1$

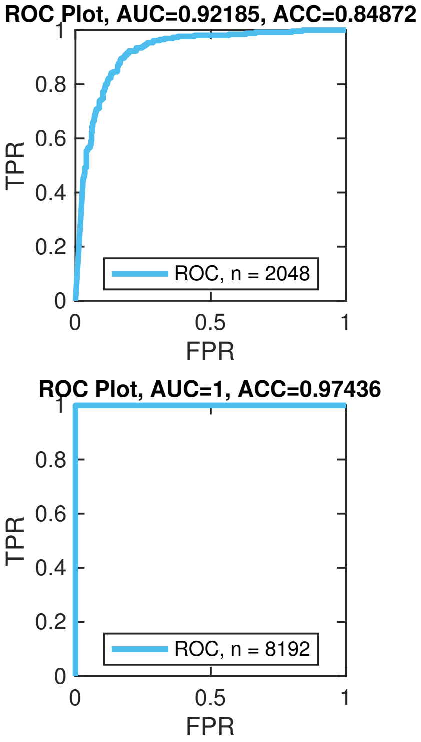

Figure S44: SVM results for Load= high, Rotational Speed = 3000 rpm, D = 6, and $\tau = 1$

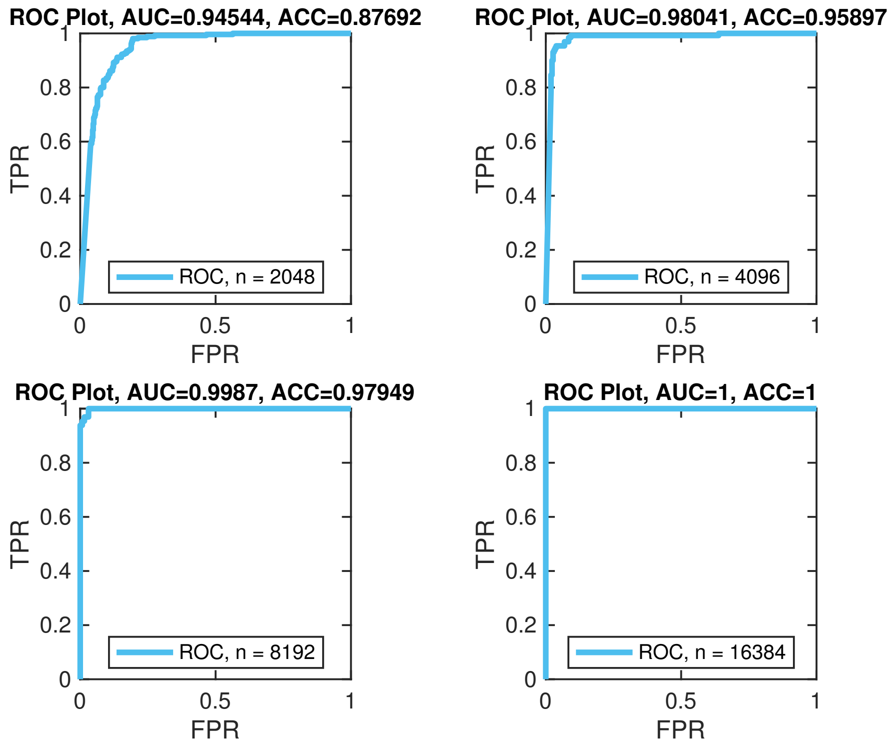

Figure S45: SVM results for Load= low, Rotational Speed = 3000 rpm, $D = 6$, and $\tau = 1$

4.3 Classifier Accuracy for all Operating Conditions

We have included the results of the sensitivity analysis and the SVM classification for two cases. We observe the same pattern for other operating conditions where the classifier performance improves with respect to increase in the signal length. The plots for all conditions are not shown to conserve space. The SVM results all operating conditions, and parameters, $D = 6$ and $\tau = 1$ are presented in Table S5.

Table S5: SVM classification accuracy in percentage for different load conditions, rotational speed conditions, and signal length N

**Load**	**Speed (rps)**	Accuracy for $N = 2048$	Accuracy for $N = 4096$	Accuracy for $N = 8192$	Accuracy for $N = 16384$
High	50 rps (3000 rpm)	84.87	95.64	97.43	98.94
High	45 rps (2700 rpm)	84.7	93.3	99	100
High	40 rps (2400 rpm)	84.9	93.3	100	100
High	35 rps (2100 rpm)	80.25	87.94	94.35	98.94
High	30 rps (1800 rpm)	69.87	77.43	85.64	91.68
Low	50 rps (3000 rpm)	87.69	95.89	97.94	100
Low	45 rps (2700 rpm)	83.1	90.2	95.3	97.8
Low	40 rps (2400 rpm)	81.3	91.7	98.4	100
Low	35 rps (2100 rpm)	73.71	79.48	87.17	98
Low	30 rps (1800 rpm)	67.94	70	77.43	85.36
